# Spatial Heterogeneity in Women’s Financial Inclusion in India: An application of small area estimation

**DOI:** 10.1371/journal.pone.0347585

**Published:** 2026-04-28

**Authors:** Swati Srivastava, Kaushalendra Kumar, Lotus McDougal, Apoorva Nambiar, Ashish Kumar Upadhyay, Katherine Hay, Anita Raj, Abhishek Singh

**Affiliations:** 1 GENDER Project, International Institute for Population Sciences, Mumbai, Maharashtra, India; 2 Department of Public Health & Mortality Studies, International Institute for Population Sciences, Mumbai, Maharashtra, India; 3 Center on Gender Equity and Health, University of California San Diego, La Jolla, California, United States of America; 4 Postdoctoral Researcher, University of Strasbourg, Strabourg, France; 5 Newcomb Institute, Tulane University, New Orleans, Louisiana, United States of America; 6 Tulane School of Public Health and Tropical Medicine, New Orleans, Louisiana, United States of America; Forest Survey of India, INDIA

## Abstract

**Background:**

While India is making notable strides in women’s financial inclusion, closing remaining gaps is hampered by a lack of granular data at the sub-state level. This study uses model-based small-area estimation techniques, linking data of National Family Health Survey 2015−16 and 2019−21, respectively, with the 2011 Indian Census data to generate district-level estimates of three components of women’s financial inclusion: women’s bank/savings account ownership, their knowledge and use of microcredit programmes in 640 districts of India in 2015−16 and 2019−21.

**Results:**

Model-based estimates reveal considerable intra- and inter-state variations in all indicators. In NFHS-5, estimates of bank/savings account ownership varied from 56.1% in Kiphire district of Nagaland to 95.4% in Perambalur district of Tamil Nadu. Knowledge of microcredit programmes varied from 11.8% in Lawngtlai district of Mizoram to a maximum of 84.9% in Thiruvarur district of Tamil Nadu. The use of microcredit programmes was more limited (7.7%). Substantial within state clustering was present in all the three components. Districts with lower prevalences in NFHS-4 registered higher increase between NFHS-4 and NFHS-5 in women’s bank/savings account ownership and their knowledge of microcredit programmes.

**Conclusions:**

Present study reveals considerable intra-state variations in both rates of financial inclusion and changes in financial inclusion levels over time. Our approach provides a new tool for policy makers to identify and close remaining gaps in women’s financial inclusion.

## Introduction

Financial inclusion has often been defined as access to bank accounts, credit, savings, financial services [1,2], and increasingly, to mobile banking services. Financial inclusion is understood to be a critical enabler of 7 out of 17 of Sustainable Development Goals (SDGs) [2–4]. The aspiration behind a financially inclusive society is to build resilient livelihoods among women by enhancing their engagement in financial activities. Women’s access to inclusive and equitable financial services has many benefits at individual as well as household levels. Financially independent women have higher negotiating power in their households, which is associated with more favourable health outcomes of their children, reduced vulnerability to domestic violence, and higher socio-economic status [5–7]. Use of financial services also offers women a means of creating an effective savings strategy, creating a financial safeguard for themselves and their families against exogenous income shocks caused by pandemics and natural disasters [8].

In 2014, Government of India launched the Pradhan Mantri Jan Dhan Yojana (PMJDY) to support every adult to have a bank account inclusive of mobile banking [9]. The aim of the PMJDY was to reach to the poor, women, and other socially marginalized groups in India. Recent reports indeed suggest that women’s ownership of a bank/savings account accelerated after the launch of PMJDY, leading to a reduction in the gender gap. Estimates from the 2019−21 National Family Health Survey (NFHS-5) indicate that more than three-fourths of ever-married women age 15−49 (78%) own a bank account, a significant jump from just over half (53%) in 2015−16 [10,11]. These estimates, however, vary substantially across India. There are wide interstate disparities in women’s ownership of a bank/savings account, ranging from 64% in Nagaland to 93% in Puducherry.

Given that the largest gaps in financial inclusion tend to be among the poor, rural or other marginalized groups [10,12], microcredit as distinct from credits of all other types is an important means of enhancing economic opportunities among women, often operationalized through self-help groups (SHGs) in India [5,13,14]. While SHGs initially focused on building women’s saving skills, they have evolved to create pathways towards economic empowerment by providing exposure to income-generating activities and connecting women with accessible financial services [15]. In recent years, India has witnessed a growth in women’s access to financial services, memberships to SHGs and microcredit programmes, in part due to improved governmental investments in these efforts. The government of India has promoted the creation of women’s SHGs within the National Rural Livelihoods Mission under Deendayal Antyodaya Yojana. According to the 2022–2023 Indian Economic Survey, there are approximately 120 million SHGs across India, serving 142 million households and overwhelmingly (88%) comprised of women. The 2022–2023 Indian Economic Survey noted that SHGs can play a vital role, particularly for rural women, for achieving financial inclusion, livelihood diversification, and skill development [16]. In this connection, the recent data shows huge variations in women’s awareness and use of microcredit programmes across India. Women’s awareness of microcredit programmes ranged from 14% in Mizoram to 80% in Puducherry [11].

While some research has explored the prevalence of several measures of financial inclusion in India, this work lacks detailed gender disaggregated analyses, and does not provide comprehensive estimates of women’s financial inclusion at the lower administrative levels, such as districts, in India [17,18]. To understand women’s financial inclusion in India in a more policy-relevant format, it is essential to have these estimates at the district level. In this study we look at two critical components of financial inclusion: bank accounts and loans. Specifically, we look at: ownership of a bank/savings account and knowledge and use of microcredit programme. As a set, these cover components of financial inclusion that include both basic and more advanced financial services and products, as well as financial awareness which is also a critical dimension of financial inclusion. In addition, we provide the estimates of women’s financial inclusion across the 640 districts of India. This work is novel as, only one study, using NFHS-4 and NFHS-5, has examined progress in women’s ownership of bank/savings account across the districts of India [19].

The NFHS, among others, has emerged as a key source of data providing estimates of women’s ownership of a bank/savings account and knowledge and use of microcredit programmes. However, the design of this survey offers representative estimates of these indicators only at the national and state level, with sample sizes limitations restricting reliable district-level estimates of women’s financial inclusion. Small-area estimation (SAE) technique has emerged as a methodologically sound way to derive these estimates at the district level [20,21]. This study fills the gap in the existing literature by using area-level SAE techniques to derive reliable estimates of women’s ownership of a bank/savings account and knowledge and use of microcredit programme for the 640 districts of India. We used these derived estimates to examine district-level spatial heterogeneity of each of the three indicators to determine if state estimates are masking potential micro-level hotspots in India. Additionally, we examine the changes in spatial heterogeneity of ownership of a bank/savings account and knowledge and use of microcredit programme between NFHS-4 and NFHS-5.

The findings of our study may contribute to the debate on women’s financial inclusion in several important ways. First, by generating estimates at subnational levels, our study identifies geographies with unusually low access to financial services. These areas can then be appropriately resourced and targeted. Identifying such hotspots may help close remaining gaps through focused interventions. Our study also highlights geographies where changes between NFHS-4 and NFHS-5 were particularly pronounced. Lessons from these areas can inform the design of targeted interventions in geographies where progress between the two survey rounds has been limited.

## Materials and methods

### Data

Our study is based on analysis of secondary data: two rounds of the NFHS, namely NFHS-4 (2015−16) and NFHS-5 (2019−21), and the 2011 Indian Population and Housing Census. Although the number of districts in India increased from 640 to 707 between NFHS-4 and NFHS-5, this analysis is necessarily limited to the 640 districts included in the 2011 Indian census. We therefore reclassified the 707 districts surveyed in NFHS-5 into 640 districts as per NFHS-4 and the most recent Indian census. The details regarding the reclassification of districts is shown in the Appendix S1Table, which also includes statistics of boundary changes between NFHS-4 and NFHS-5.

The NFHS is a large-scale, multi-round survey conducted in a representative sample of households throughout India. NFHS-4 interviewed 699,686 women age 15−49 between 20 January 2015 and 4 December 2016. In NFHS-5, 724,115 women between ages 15−49 were interviewed. Data collection of NFHS-5 was conducted in two phases due to the COVID-19 pandemic, with Phase 1 occurring between 17 June 2019 and 30 January 2020 and Phase 2 between 2 January 2020 and 30 April 2021. The individual women’s response rate was 97% in both NFHS-4 and NFHS-5 [10,11]. As these cross-sectional surveys were observational in nature, there was no blinding of participants.

The 2011 Indian Population and Housing Census was conducted between February 9–28, 2011, and included information on a number of socio-economic characteristics, housing characteristics, fertility, and migration [22].

### Study participants

In NFHS-4 and NFHS-5, a total of 724,115 and 699,686 women age 15−49 were interviewed across 28 Indian states and 8 Union Territories (for state/UT map see appendix S1 Fig). While only 122,351 and 108,416 women were randomly selected to receive the women’s financial inclusion module containing questions related to ownership of a bank/savings account and knowledge and use of microcredit programme in NFHS-4 and NFHS-5, respectively. In women’s financial inclusion module in NFHS-4, the average number of women per district was 191, ranging from 54 to 483, whereas in NFHS-5 it was 169, ranging from 14 to 631.

### Outcome variables

The three primary study outcome variables are district-level prevalence of women 1) who have access to a bank/savings account that they themselves use, 2) who are aware about microcredit program and 3) who have taken a loan from microcredit institution irrespective of their level of knowledge. Details about these outcome variables can be found in the Appendix S1 File.

### Auxiliary information

In SAE analysis, two types of variables are required, the outcome and auxiliary variables. Outcome variables measure the outcome of interest, and are usually derived from surveys. Auxiliary variables capture specific sociodemographic factors, and are required from the entire population. Typically, these auxiliary variables are available in the Census or administrative records [23]. We included the following district-level information from the 2011 Indian Population and Housing Census as auxiliary variables: scheduled caste or tribes, Muslim religion, female workforce participation rates and gender gap in workforce participation, female literacy level and gender gaps in literacy, female headed household, household size, female age at marriage, spousal age gap, male-to-female child ratio among women aged 30–49 years, media exposure (TV and radio), urban residence, socio-economic status, and the state of residence. The choice of auxiliary variables for our models is guided by the social, economic, and demographic determinants of financial services identified in earlier literature [24–26]. We included proportion of villages having self-help group and proportion of villages having any commercial or cooperative bank as additional auxiliary variables [26–28]. Details of auxiliary variables are provided in the Appendix S2 File.

### Statistical analysis

NFHS-4 and NFHS-5 were not designed to provide estimates of women’s ownership of a bank/savings account and knowledge and use of microcredit programmes for the districts of India. Due to this the sample sizes in NFHS-4 and NFHS-5 are not enough to provide reliable estimates of these indicators for the 640 districts of India. SAE is a promising technique for addressing the issue of small sample sizes in household surveys, such as NFHS-4 and NFHS-5 [20,21,29]. SAE techniques are classified into two broad types: unit-level and area-level models. Unit-level models are used when auxiliary data are available at the individual level, whereas area-level models are used when auxiliary variables are only available at aggregate (e.g., district) level [23,30,31].

We adopted the area-level SAE approach, as auxiliary data were only available at district-level. We first derived district- level estimates of women’s ownership of a bank/savings account and knowledge and use of microcredit programme directly from the NFHS-4 and NFHS-5, accounting for survey weights; these estimates are henceforth called as direct survey-based estimates. These direct survey-based estimates were then linked to auxiliary variables using Generalized Linear Mixed Models (GLMM) with logit link functions to derive the model-based district-level estimates for the 640 districts of India [23,30,32]. The GLMM accounted for area-specific random effects, which provided strength to the model-based district-level estimates of the women’s ownership of a bank/savings account and knowledge and use of microcredit programme [33].

Two types of diagnostic measures (model diagnostic and diagnostic for the small-area estimates) are used in order to assess the validity of the fitted GLMM models and the reliability of the model-based district-level estimates of women’s ownership of a bank/savings account and knowledge and use of microcredit programme. We used model diagnostic and diagnostic for the small-area estimates for assessing the validity and reliability of our estimates. The details of the two diagnostic measures are given in the Appendix S3 File. A p < 0.05 was considered significant. Survey weights given in NFHS-4 and NFHS-5 were used in the estimations.

We mapped our estimates to identify clusters of districts of high and low women’s financial inclusion. Spatial heterogeneity analysis may help identify and target cluster of districts with low coverage of financial services even in higher prevalence state contexts. We used Moran’s I, univariate Local Indicator of Spatial Association (LISA) and bivariate LISA to obtain geographical clustering of women’s access to financial services in India.

Moran’s I is the Pearson coefficient measure of spatial autocorrelation. Spatial autocorrelation measures the degree to which data points are similar or dissimilar to their spatial neighbors. Moran-I is given by


$$Moran\text{'}s\text{\textbackslash{}hspace\{0.17em}\}I=C\text{\textbackslash{}hspace\{0.17em}\;\}X\text{\textbackslash{}hspace\{0.17em}\;\}\frac{\sum_{ij}w_{ij}z_{i}z_{j}}{\sum_{i}\overset{\text{2}}{\underset{i}{z}}}$$

Z_i_: standardized variable of interest;

W_ij_: weight matrix;

Negative (positive) values indicate negative (positive) spatial autocorrelation. Positive autocorrelation indicates that points with similar attribute values are closely distributed in space whereas negative spatial autocorrelation indicates that closely associated points are more dissimilar. Values range from −1 (indicating perfect dispersion) to +1 (perfect correlation). A zero value indicates a random spatial pattern.

Univariate LISA, on the other hand, measures the correlation of neighborhood values around a specific spatial location. It determines the extent of spatial non-stationery and clustering present in the data. It is given by


$$I_{i}=\text{\textbackslash{}hspace\{0.17em}\;\}z_{i}\sum_{j}w_{ij}z_{j}$$

Univariate LISA shows high-high clustering (high prevalent districts surrounded by high prevalent neighborhood), low-low clustering (low prevalent districts surrounded by low prevalent neighborhood), and spatial outliers (low–high and high-low clusters). The high-high clusters are also called hot spots, while the low-low clusters are called cold spots. The districts marked as not significant are those which are surrounded by districts with different patterns of women’s financial inclusion.

Bivariate LISA measures the local correlation between a variable and weighted average of another variable in the neighborhood.


$$I_{i}=\text{\textbackslash{}hspace\{0.17em}\;\}n_{i}\sum_{j}w_{ij}z_{j}$$

Similar to univariate LISA, bivariate LISA identifies high-high, low-low, high-low, and low-high clusters. We used bivariate LISA to examine the relationship between women’s access to financial services in NFHS-4 and changes in women’s access to financial services between NFHS-4 and NFHS-5 across districts of India.

Bivariate LISA provides insights into how two variables co-vary across geography. It identifies districts where the level of one variable (e.g., women’s financial inclusion in NFHS-4) is spatially associated with the neighboring values of another variable (e.g., subsequent change between NFHS-4 and NFHS-5). This helps determine whether improvements in financial inclusion are concentrated in districts that were already performing well or whether lagging districts are catching up. Such information is valuable for guiding targeted policy interventions.

We used Queen’s contiguity weight matrix to estimate both univariate and bivariate LISA. The LISA were estimated using GeoDa version 1.12.1.161. SAE was carried out in STATA 16.

## Results

Among the SAE sample, a total of 64.1% (n = 77610) women in NFHS-4 and 67.8% (n = 73128) women in NFHS-5 lived in rural areas. With respect to social group, 28.5% (n = 34534) women in NFHS-4 and 30.9% (n = 33324) women in NFHS-5 belonged to the scheduled caste or tribe; and 80.1% (n = 97299) women in NFHS-4 and 80.7% (n = 87129) women in NFHS-5 were Hindu. A total of 16.0% (n = 19430) women in NFHS-4 and 18.4% (n = 19903) women in NFHS-5 were from the bottom 20% of wealth quintile; whereas 26.2% (n = 31712) women in NFHS-4 and 22.5% (n = 24322) women in NFHS-5 had no formal schooling. With respect to the financial inclusion parameters, in NFHS-4, 53.0% (95% CI: 52.4, 53.6) of women had ownership of a bank/savings account, 40.8% (40.1-40.8) were aware of microcredit programmes, and only 7.7% (7.4-8.1) had utilized microcredit programmes. Whereas, in NFHS-5, these percentages increased to 78.6% (95% CI: 78.1-79.1), 51.3% (50.5-52.0), and 11.1% (10.7-11.4), respectively (Table 1).

**Table 1:** Sample characteristics and prevalence of women’s ownership of a bank/savings account and knowledge and use of microcredit programme by selected characteristics in India, NFHS-4 (2015−16) and NFHS-5 (2019−21).

	NFHS-4					NFHS-5				
	N (Weighted)	% (Weighed)	Ownership of a bank/savings account	Knowledge of microcredit programme	Use of microcredit programme	N (Weighted)	% (Weighed)	Ownership of a bank/savings account	Knowledge of microcredit programme	Use of microcredit programme
			% (95% CI)	% (95% CI)	% (95% CI)			% (95% CI)	% (95% CI)	% (95% CI)
**India**	1,21,120	100.0	53.0(52.4, 53.6)	40.8(40.1, 41.6)	7.7 (7.4, 8.1)	1,08,014	100.0	78.6(78.1, 79.1)	51.3(50.5,52.0)	11.1(10.7,11.4)
**Residence**										
Urban	43510	35.92	61.0(59.7,62.2)	45.2(43.6,46.8)	7.6(7.0,8.3)	34,839	32.25	80.9(80.0,81.9)	51.4(49.9,52.8)	9.4(8.7,10.1)
Rural	77610	64.08	48.5(47.9,49.1)	38.4(37.6,39.2)	7.8(7.5,8.1)	73,175	67.75	77.4(76.9,78.0)	51.2(50.4,52.1)	11.9(11.5,12.3)
**Caste**										
Schedule caste	23524	19.42	54.4(53.1,55.7)	41.1(39.5,42.7)	9.2(8.4,9.9)	23,427	21.7	79.4(78.6,80.3)	52.0(50.5,53.6)	13.0(12.2,13.7)
Schedule tribes	11010	9.09	44.5(43.1,45.9)	36.5(34.8,38.2)	7.2(6.2,8.2)	9,909	9.18	75.0(73.7,76.3)	47.1(45.4,48.8)	10.8(9.9,11.6)
Other backward classes	53500	44.17	53.5(52.8,54.3)	41.0(40.1,41.9)	8.3(7.8,8.8)	47,251	43.73	79.7(79.1,80.3)	53.2(52.3,54.1)	11.6(11.1,12.1)
Others	33086	27.32	53.9(52.7,55.2)	41.9(40.5,43.3)	6.0(5.4,6.6)	27,427	25.4	77.2(76.2,78.2)	48.8(47.4,50.2)	8.7(8.0,9.3)
**Religion**										
Hindu	97299	80.33	54.0(53.4,54.6)	41.3(40.5,42.1)	8.1(7.7,8.4)	87,179	80.72	79.3(78.8,79.8)	52.3(51.5,53.1)	11.7(11.3,12.1)
Muslim	17295	14.28	44.5(42.9,46.0)	35.4(33.6,37.2)	5.6(4.7,6.5)	15,138	14.0	73.4(72.2,74.7)	45.6(43.6,47.6)	7.9(7.2,8.6)
Christian	3064	2.53	66.9(64.4,69.5)	52.2(49.0,55.4)	12.5(9.8,15.2)	2,495	2.31	81.7(79.2,84.1)	56.4(53.2,59.7)	14.0(11.6,16.5)
Sikh	1702	1.41	59.5(56.4,62.7)	44.1(39.5,48.8)	2.5(1.7,3.3)	1,908	1.77	82.4(79.5,85.2)	43.4(39.1,47.8)	7.6(6.1,9.1)
Others	1760	1.45	50.7(44.6,56.8)	45.9(40.5,51.4)	6.2(3.6,8.9)	1,294	1.2	77.8(74.3,81.3)	52.8(47.7,58.0)	9.2(7.0,11.5)
**Wealth index**										
Poorest	19430	16.04	34.1(33.1,35.0)	29.0(27.8,30.1)	5.4(4.9,5.9)	19,904	18.44	71.7(70.6,72.7)	47.7(46.3,49.1)	10.9(10.3,11.6)
Poorer	22843	18.86	43.8(42.8,44.7)	36.3(35.2,37.4)	7.5(7.0,8.0)	22,049	20.42	76.6(75.7,77.5)	50.0(48.7,51.2)	12.7(12.0,13.5)
Middle	25232	20.83	53.0(52.0,54.0)	42.2(41.0,43.3)	9.9(9.3,10.5)	22,252	20.6	79.4(78.5,80.2)	53.2(51.9,54.5)	13.2(12.5,13.8)
Richer	26358	21.76	59.4(58.4,60.5)	45.1(43.8,46.5)	9.7(9.0,10.5)	22,376	20.7	80.4(79.5,81.3)	53.4(52.1,54.7)	11.2(10.5,11.9)
Richest	27256	22.5	67.9(66.6,69.3)	47.7(46.0,49.5)	5.7(5.0,6.4)	21,432	19.85	84.3(83.3,85.3)	51.7(50.2,53.2)	7.3(6.6,8.0)
**Education**										
No education	31712	26.18	43.2(42.4,44.0)	33.3(32.3,34.2)	8.9(8.3,9.4)	24,352	22.53	75.1(74.3,75.9)	47.5(46.4,48.6)	13.8(13.2,14.4)
Primary	14866	12.27	44.8(43.5,46.1)	38.3(37.0,39.6)	9.9(9.1,10.6)	12,664	11.72	75.0(73.9,76.1)	51.3(49.9,52.7)	15.4(14.4,16.3)
Secondary	58316	48.15	53.5(52.7,54.2)	42.7(41.8,43.6)	7.4(7.0,7.8)	54,095	50.09	77.6(76.9,78.2)	51.3(50.4,52.2)	10.3(9.9,10.8)
Higher	16227	13.4	77.9(76.7,79.1)	51.3(49.6,52.9)	4.8(4.0,5.6)	16,903	15.66	89.4(88.7,90.2)	56.6(55.2,58.0)	6.4(5.7,7.1)

Sources: Author’s calculation based on the NFHS-4 and NFHS-5 dataset.

District-level residuals of women’s ownership of a bank/savings account and knowledge and use of microcredit programmes are randomly distributed, confirming the normality assumption in both the survey rounds. This suggests that the model-based estimates of women’s ownership of a bank/savings account and knowledge and use of microcredit programmes are robust, with means closer to their expected values (appendix S2 Fig). We compared the closeness of the 45^0^ line (y = x) to the fitted regression line for the model-based and direct survey-based estimates in order to examine the level of consistency between the two. The line of best fit was not significantly different from the line y = x at five % level for the model-based estimates, indicating the consistency between the model-based and direct survey-based estimates (appendix S3 Fig). The coefficient of variation (CVs) of the direct survey-based estimates are much larger than the model-based estimates (appendix S4 Fig). Additionally, the fluctuations in the CVs of the direct survey-based estimates are considerably larger compared to those of the model-based estimates. This diagnostic indicates that the model-based estimates are more precise than the direct survey-based estimates. Appendix S5 Fig shows the 95% CIs of the direct survey-based estimates and the model-based estimates. The direct survey-based estimates have much wider 95% CIs compared to the model-based estimates, suggesting that the standard errors of the direct survey-based estimates are large and unreliable. The model-based estimates are more robust than the direct survey-based estimates. The diagnostics clearly show the power of the SAE techniques for producing unbiased, consistent, and reliable estimates of women’s ownership of a bank/savings account and knowledge and use of microcredit programme in the 640 districts of India.

**Fig 1:**
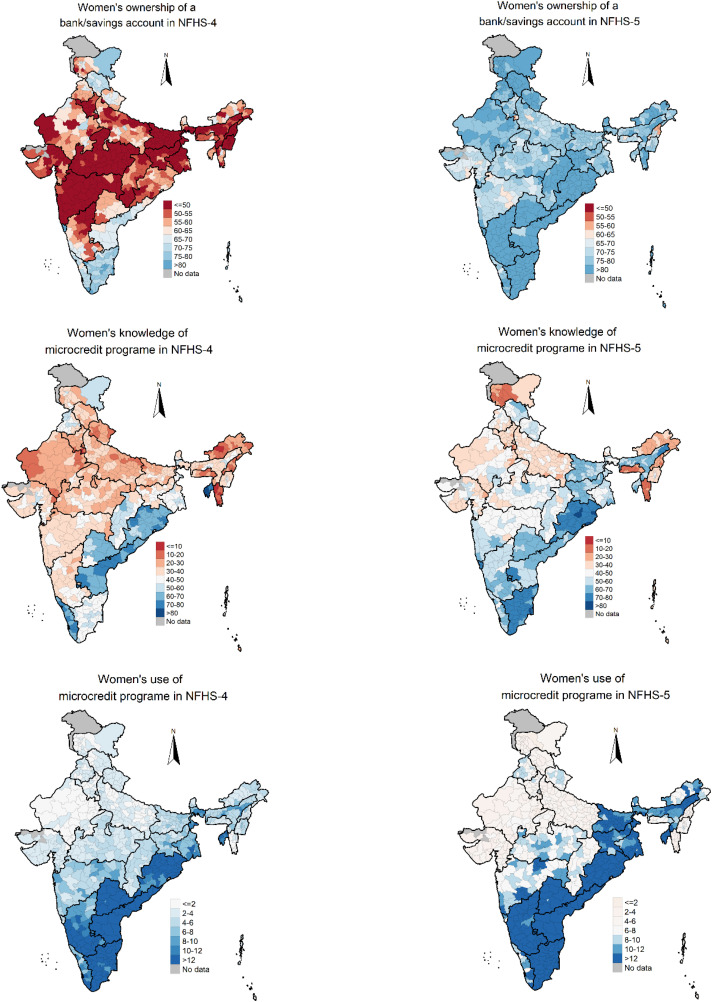
Model-based estimates of women’s ownership of a bank/savings account and knowledge and use of microcredit programme in India, NFHS-4 and NFHS-5. **Sources:** The shape file downloaded from Spatial Data Repository – Boundaries, The Demographic and Health Surveys Program, ICF International, funded by the United States Agency for International Development (USAID). Available from “https://spatialdata.dhsprogram.com/boundaries/” https://spatialdata.dhsprogram.com/boundaries/#view=table&countryId=IA [Accessed 22 February, 2023]..

The model-based estimates of women’s ownership of a bank/savings account and knowledge and use of microcredit programmes varied considerably across the districts of India (Fig 1). In NFHS-5, estimates of ownership of a bank/savings account varied from a minimum of 56.1% (95% CI: 53.4%, 58.7%) in Kiphire district of Nagaland to a maximum of 95.4% (95% CI: 95.0%, 95.7%) in Perambalur district of Tamil Nadu. The estimated prevalence of knowledge of microcredit programme varied from as low as 11.8% (95% CI: 10.5%, 13.1%) in Lawngtlai district of Mizoram to a maximum of 84.9% (95% CI: 84.4%, 85.2%) in Thiruvarur district of Tamil Nadu. In Chandigarh, less than one % i.e., 0.7% (95% CI: 0.6%, 0.8%) of women used microcredit programme, whereas the maximum use was found in Krishna district (41.8% (95% CI: 41.5%, 42.1%)) of Andhra Pradesh.

We found considerable intra-state variations in women’s ownership of a bank/savings account and knowledge and use of microcredit programme. For example, in Nagaland, the state with the lowest coverage of women’s ownership of a bank/savings account (63.7% (95% CI: 60.7-66.6)), the district-level estimates varied from 56.1% (95% CI: 53.4-58.7) in Kiphire to 73.2% (72.0-74.3) in Kohima. The prevalence of women’s ownership of a bank/savings account in Gujarat was 70.2% (95% CI: 68.5-71.8), which was close to Nagaland. The lowest and highest prevalence in Gujarat were estimated for Kheda (61.3%,95% CI: 60.9-61.7) and Navsari (81.7%,95% CI: 81.3-82.2) districts, respectively. The highest percentage of women’s ownership of a bank/savings account was observed in Tamil Nadu (92.2%, 95% CI: 90.9-93.3), with prevalence ranging from 89.5% (95% CI: 89.3-89.7) in Salem to 95.4% (95% CI: 95.0-95.7) in Perambalur.

Intra-state heterogeneity was also observed in the prevalence of women’s knowledge of microcredit programme. For instance, in Mizoram, with a prevalence of 13.8% (95% CI: 11.4-16.6), the prevalence of women’s knowledge of microcredit programme ranged between 11.8% (10.5-13.1) in Lawngtlai and 20.2% (18.1-22.3) in Serchhip. Jammu and Kashmir followed Mizoram, with a prevalence of 20.6% (95% CI: 19.1-22.2), where the districts prevalence ranged between 14.2% (95% CI: 13.5-14.9) in Punch to 31.5% (29.8-33.3) in Ladakh. Among states, the prevalence of women’s knowledge of microcredit programme was highest in Odisha at 74.4% (95% CI: 72.9-75.9), ranging from 66.8% (66.3-67.3) in Koraput to 82.6% (82.2-83.1) in Angul. The use of microcredit programmes in Mizoram, the state with the lowest coverage, was estimated at 2.1% (95% CI: 1.3-3.3), ranging between 1.3% (0.8-1.8) in Lawngtlai and 4.8% (3.7.6-6.0) in Serchhip. The highest prevalence of microcredit programme usage was observed in Andhra Pradesh at 29.7% (95% CI: 27.5-32.1), with district prevalence ranging between 20.9% (20.6-21.2) in SPSR Nellore to 41.8% (41.5-42.1) in Krishna.

While Nagaland had the lowest prevalence of women’s ownership of a bank/savings account, it had the highest variation across the districts, measured in terms of CVs for the model-based estimates (0.105) (see appendix S2 Table). Haryana (0.095) and Gujarat (0.068) closely followed Nagaland in terms of variation in women’s ownership of a bank/savings account across the districts. The lowest variation in women’s ownership of a bank/savings account was observed in Goa (0.014) followed by Tamil Nadu (0.035). In terms of knowledge of microcredit programs, Jammu and Kashmir had the highest inter-district variation (0.212), followed by Haryana (0.173), Mizoram (0.171) and Manipur (0.162). The lowest inter-district variation in knowledge of microcredit programmes was in Tamil Nadu (0.046), followed by Odisha (0.053) and Bihar (0.057). In case of use of microcredit programmes, the highest inter-district variation was found in Jammu and Kashmir (0.392), followed by Mizoram (0.371) and Rajasthan (0.347). The lowest inter-district variation in the use of microcredit programmes was found in Tripura (0.083).

**Fig 2:**
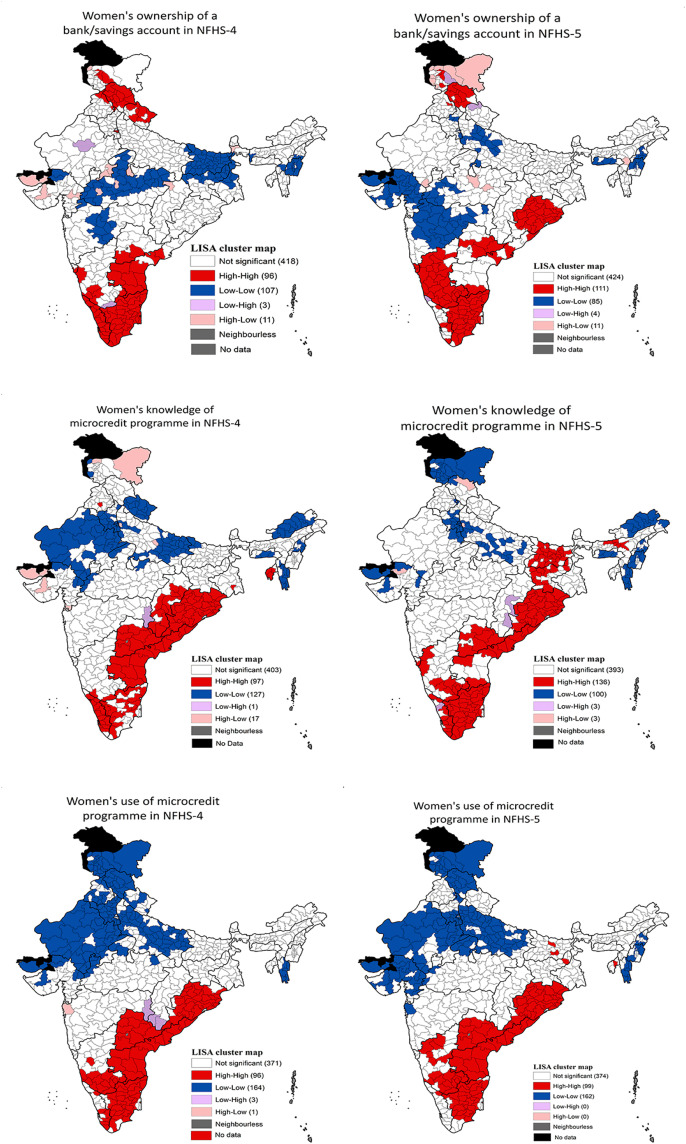
Univariate LISA maps depicting women’s ownership of a bank/savings account and knowledge and use of microcredit programme in India, NFHS-4 and NFHS-5. **Sources:** The shape file downloaded from Spatial Data Repository – Boundaries, The Demographic and Health Surveys Program, ICF International, funded by the United States Agency for International Development (USAID). Available from “https://spatialdata.dhsprogram.com/boundaries/”https://spatialdata.dhsprogram.com/boundaries/#view=table&countryId=IA [Accessed 22 February, 2023].

Univariate LISA maps shown in Fig 2 depict the spatial heterogeneity in women’s ownership of a bank/savings account and women’s knowledge and use of microcredit programmes in NFHS-4 and NFHS-5. In NFHS-5, high-high spatial clusters of women’s ownership of a bank/savings account were observed primarily in Tamil Nadu, Karnataka, Odisha, Himachal Pradesh, Goa and Andhra Pradesh. A few districts of Kerala, Jammu and Kashmir, Punjab, and Puducherry also formed high-high spatial clusters of women’s ownership of a bank/savings account. In contrast, cold-spots of women’s ownership of a bank/savings account were located primarily in Gujarat, Maharashtra, Uttar Pradesh, NCT of Delhi, Madhya Pradesh, Haryana, Manipur, Meghalaya and Nagaland.

High-high spatial clusters of knowledge of microcredit programme, in NFHS-5, were found in Andhra Pradesh, Odisha, Tamil Nadu, Bihar, Jharkhand, Karnataka, Kerala, and a few districts of Assam and Puducherry. In contrast, low-low spatial clusters of knowledge of microcredit programme were located in 100 districts primarily from Arunachal Pradesh, Haryana, Jammu and Kashmir, NCT of Delhi, Mizoram, and a few districts of Gujarat, Himachal Pradesh, Manipur, Meghalaya, Nagaland, Punjab and Rajasthan. High-high clustering of use of microcredit programme was found primarily in districts of Andhra Pradesh, Karnataka, Odisha, Tamil Nadu and a few districts of Bihar, Jharkhand, Kerala, Puducherry and Tripura. On the other hand, a total of 162 districts showed low-low spatial clustering; these districts primarily belonged to Uttar Pradesh, Rajasthan, Gujarat, Haryana, Himachal Pradesh, Jammu and Kashmir, NCT of Delhi, and a few districts of Madhya Pradesh, Maharashtra, Manipur, Mizoram, Nagaland and Punjab.

Change analysis shows that in 2015−16, women’s ownership of a bank/savings account was less than 50% in 267 districts, whereas in 2019−21, no districts had estimates lower than 50% (see appendix S3 Table). The number of districts having at least four-fifth of women (80%) owning a bank/savings account increased from 11 districts in 2015−16 to 281 districts in 2019−21. In NFHS-4, percentage of women knowing about microcredit programme was less than 50% in 521 districts, whereas in NFHS-5, only 354 districts have estimates lower than 50%. Most districts saw low microcredit programme utilization; 520 districts in NFHS-4 and 388 districts in NFHS-5 had less than 10% of women who used these microcredit programmes. The number of districts where more than 20% women used microcredit programmes increased from 25 districts in NFHS-4 to 77 districts in NFHS-5.

**Fig 3:**
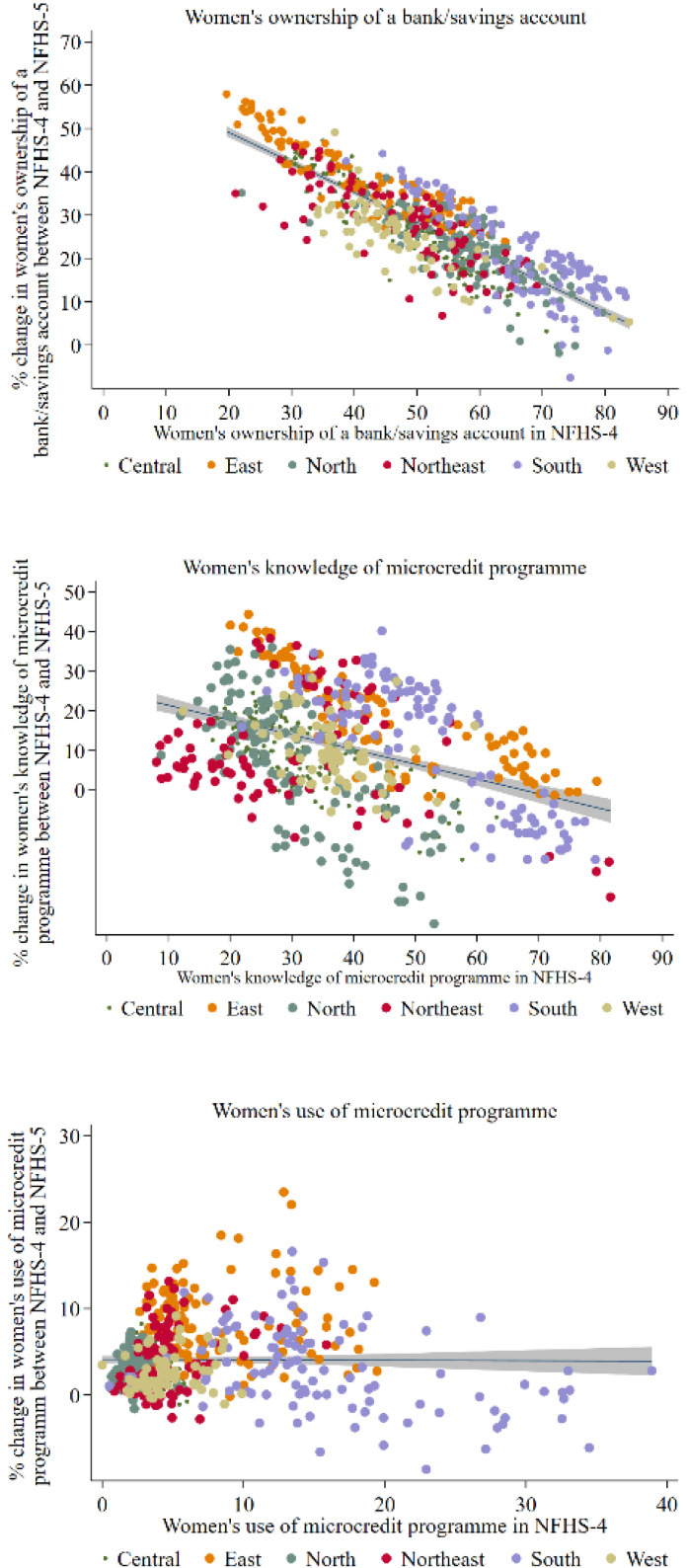
Association between prevalence of indicators in NFHS-4 and changes in between NFHS-4 and NFHS-5 in India. Sources: Author’s calculation based on the NFHS-4 and NFHS-5 datasets.

Fig 3 shows the association of three indicators of women’s financial inclusion in NFHS-4 with the percent point change in those indicators between NFHS-4 and NFHS-5. Districts that had lower coverage of a bank/savings account among women in NFHS-4 registered higher increase between NFHS-4 and NFHS-5. Similarly, districts having lower knowledge of microcredit programme among women registered higher increase between NFHS-4 and NFHS-5. However, there was no association between use of microcredit programme in NFHS-4 and the change between NFHS-4 and NFHS-5.

**Fig 4:**
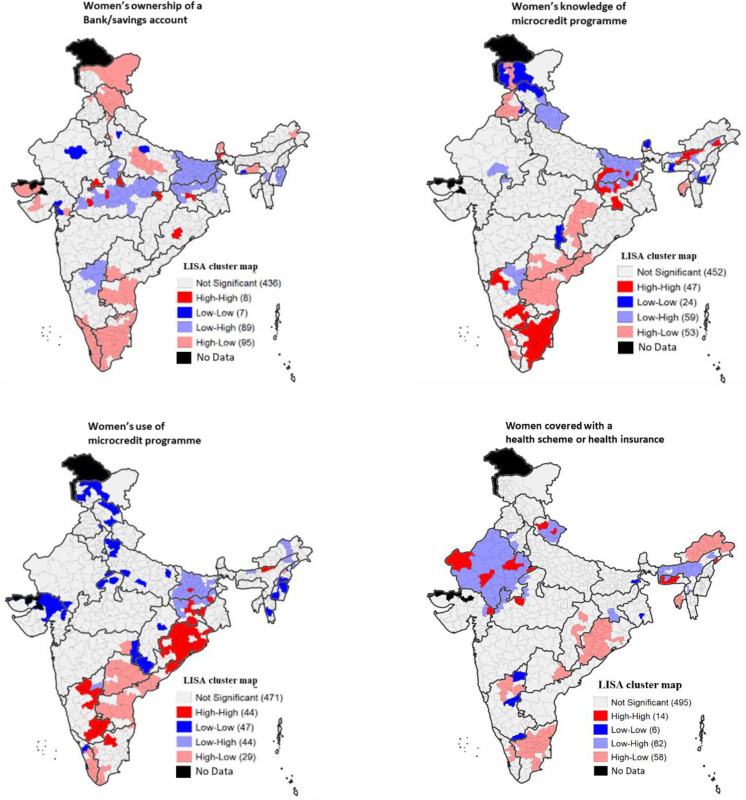
Bivariate LISA maps showing association of financial inclusion indicators in NFHS-4 with changes between NFHS-4 and NFHS-5 in districts of India. **Sources:** The shape file downloaded from Spatial Data Repository – Boundaries, The Demographic and Health Surveys Program, ICF International, funded by the United States Agency for International Development (USAID). Available from “https://spatialdata.dhsprogram.com/boundaries/”https://spatialdata.dhsprogram.com/boundaries/#view=table&countryId=IA [Accessed 22 February, 2023].

Districts from Bihar and Jharkhand, northern, Vindhya, central and Malwa divisions from Madhya Pradesh, inland northern Karnataka and plains of Manipur formed clusters that were characterized by low women’s ownership of a bank/savings account in NFHS-4 but high increase between NFHS-4 and NFHS-5 (Fig 4). In contrast, clusters with high bank/savings account ownership prevalence in NFHS-4 and low increase between NFHS-4 and NFHS-5 (High-Low) were largely located in Jhelam valley of Jammu and Kashmir, Himachal Pradesh, Kerala, Tamil Nadu, central and eastern divisions of Uttar Pradesh, dry areas and Saurashtra divisions of Gujarat, inland north western division of Telangana, and inland southern and coastal southern divisions of Andhra Pradesh (See Registrar General and Census Commissioner of India (2017) for more information on natural with-in state divisions in India).

When it comes to the knowledge of microcredit programmes, low-high (i.e., low prevalence in NFHS-4 and high increase between NFHS-4 and NFHS-5) clusters were found in Jhelam valley of Jammu and Kashmir, Uttarakhand, Bihar, Hazaribagh plateau of Jharkhand, and inland northern Karnataka. High-low clusters were found in southern Punjab, Mahanadi basin of Chhattisgarh, Tripura, Telangana, Andhra Pradesh, and southern Kerala. Central parts of Bihar, Jharkhand, central Brahmaputra plains of Assam, inland northern and inland southern Karnataka, and Tamil Nadu formed high-high clusters.

Use of microcredit programme was low in Bihar in NFHS-4 but increase was high between NFHS-4 and NFHS-5. This was also the case in Jharkhand, plains eastern of Assam, and Arunachal Pradesh. High-high clusters were found in Odisha, inland northern and inland southern Karnataka, few districts of Jharkhand and Assam and inland Tamil Nadu.

**Fig 5:**
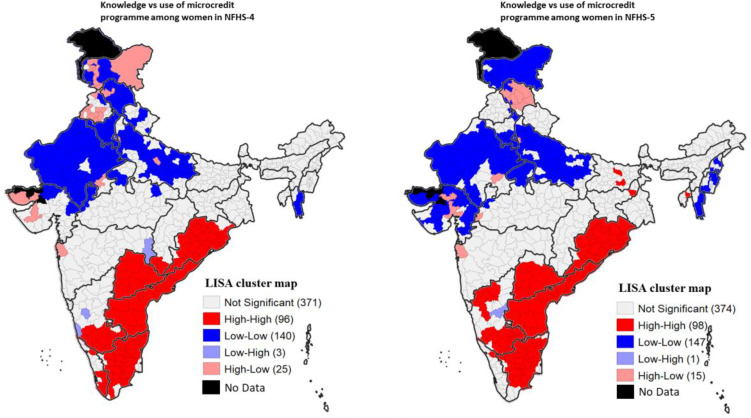
Bivariate LISA maps showing association between women’s knowledge and use of microcredit programme in districts of India, NFHS-4 and NFHS-5. **Sources:** The shape file downloaded from Spatial Data Repository – Boundaries, The Demographic and Health Surveys Program, ICF International, funded by the United States Agency for International Development (USAID). Available from “https://spatialdata.dhsprogram.com/boundaries/”https://spatialdata.dhsprogram.com/boundaries/#view=table&countryId=IA [Accessed 22 February, 2023].

Fig 5 shows bivariate association between knowledge and use of microcredit programme at the district level. In NFHS-4, low knowledge of microcredit programme was associated with low use of microcredit primarily in districts of Jammu and Kashmir, Himachal Pradesh, Uttarakhand, Haryana, Uttar Pradesh, Mizoram, and Gujarat. On the other hand, high knowledge was associated with high use primarily in districts of Odisha, Telangana, Andhra Pradesh, inland eastern and inland southern Karnataka, southern Kerala, and Tamil Nadu. The picture remained largely the same in NFHS-5. In NFHS-4, high knowledge was associated with low use primarily in districts of Punjab, Gujarat, and Leh (Ladakh). Whereas in NFHS-5, high knowledge was associated with low use primarily in the districts of Himachal Pradesh and Gujarat.

## Discussion

Our study is the first to provide robust estimates of key indicators of women’s financial inclusion, specifically ownership of a bank/savings account and knowledge and use of microcredit programmes, for the 640 districts of India. We used area-level SAE models to generate these estimates at two points in time (2015−16 and 2019−21), calculations which would be unreliable if directly estimated from NFHS surveys due to small sample sizes. Several diagnostic tests demonstrated the stability of SAE for estimating district-level estimates of ownership of a bank/savings account and awareness and use of microcredit programme in India. This study shows the utility of SAE by linking data from the Indian census with survey data to identify the potential hotspots with a large number of women who are unbanked or do not know or use microcredit programmes. Researchers and policy makers in countries where data is not readily available at the local level can take examples from this study to estimate similar key financial inclusion metrics.

These two successive rounds of NFHS confirm rapid progress in women’s ownership of a bank/savings account in India. Districts having lower percentage of women owning a bank/savings account in NFHS-4 experienced larger increases between the two surveys. While PMJDY may be a leading driver of progress, other central and state government schemes that mandate holding a bank/savings account may also be supporting these observed findings [34–36]. The Government of India has implemented Direct Benefit Transfer (DBT) in 313 schemes, many targeting women, across 53 Ministries [37]. The DBT programs created further value for owning and using a bank or savings account.

With India gaining traction on women’s bank/savings account ownership, there is now need to promote usage of these accounts and access to credit – with microcredit being a particularly important credit option for the poorest individuals. Only 51% and 11% of women in India knew of a microcredit programme and used such a programme, respectively. There were huge variations in the knowledge and use of microcredit programme across the districts of India. While women’s knowledge of these programmes ranged between 12% and 85% across districts, the use ranged between 1% and 42%. These figures indicate that a significantly high proportion of women neither have knowledge of a microcredit programme, nor did they use one; observed gaps were higher in the districts of the northern and eastern (except Odisha) parts of India. This finding is important given higher gender inequality in the northern and eastern parts of India compared with the southern region [38]; regions with greater gender inequalities broadly may also see more restrictions on women’s participation in SHGs and banking. The low use of financial services in the northern states could also be because of the slow-growth or non-existent microfinance institutions in these regions [39]. The lower utilization of microfinancing programs in hilly and north-eastern regions may also be attributed to several other factors that have often been overlooked, including the population size, challenging terrain, lower population density, and inadequate infrastructure [40].

We were able to locate the districts that had lower coverage of a bank/savings account or knowledge of microcredit programmes in NFHS-4 but the increase in coverage was higher between NFHS-4 and NFHS-5; these districts were mostly located in the northern and eastern parts of India. Importantly, the districts with lower use of microcredit programme in NFHS-4 but higher increase in use between NFHS-4 and NFHS-5 were located in Bihar and Jharkhand (both from the eastern part) and Assam and Arunachal Pradesh (both from the north-eastern part). This finding holds significance given that Bihar, Jharkhand and Assam lag behind other states in terms of fertility, mortality, and other development indicators [41,42]. Moreover, these states ranked lower in terms of gender inequality (Singh et al., 2022b). The impressive increase in Bihar and Jharkhand may be related to the JEEVIKA and AAJEEVIKA programmes, which focusses on connecting women from poor rural families with SHGs. Additionally, JEEVIKA focuses on providing these women access to financial services and markets, enabling their financial inclusion [43]. Other poor performing states, such as Rajasthan, Uttar Pradesh, Madhya Pradesh, and Chhattisgarh, may learn from the experiences of Bihar and Jharkhand as to what works when it comes to women’s financial inclusion.

Spatial inequality across the three indicators of women’s financial inclusion closely mirrors the broader gender inequality patterns in India. For instance, all three indicators were higher in districts from southern India compared to those in northern, central, and western India. Districts in the northern, central, and western regions exhibit higher levels of gender inequality than those in the southern region [38]. While we were unable to explore these intersections in greater depth, these intersections are important to examine in future research now that district-level estimation of these gender inequalities has been conducted. A recent article by Mathen et al. (2024) indeed shows how spatial inequality in nighttime satellite data can be used to predict economic activities and regional inequality in India [44].

Our direct survey estimates were significantly different (i.e., they were either higher or lower) from model-based small-area estimates for districts with small survey sample sizes. However, the two estimates tended to be similar with increasing survey sample sizes. For example, in NFHS-5, the direct-survey estimates and model-based small-area estimates for ownership of bank/savings account for Bhopal district with only 49 women age 15−49 were 63.1% (95% CI: 45.2-78.0) and 77.7% (95% CI: 77.3-78.0), respectively. The 95% confidence interval (CI) of the direct-survey estimate is wide, indicating that the estimate is unstable. In comparison, in Karimnagar district, with a survey sample of 568 women aged 15−49, the direct-survey estimate for ownership of a bank/savings account was 85.7% (95% CI: 82.4-88.4), while the model-based small-area estimate was 87.1% (95% CI: 86.9-87.3). Some of the differences between the direct-survey estimates and the model-based small-area estimates may be attributed to the selection of auxiliary variables and the relationship of these variables with the outcome of interest. To address this, we assessed the goodness of fit of our estimated models. Our models depicted good fit. For example, the coefficient of determination (R^2^) values for ownership of bank/savings account, knowledge and use of microcredit programme in NFHS-4 were 0.79, 0.72 and 0.81, respectively. The R^2^ values for ownership of bank/savings account, knowledge and use of microcredit programme in NFHS-5 were 0.64, 0.72 and 0.80, respectively. Internal validation of the district-level small-area estimates for ownership of bank/savings account, knowledge and use of microcredit programme compared with direct-survey estimates indicated a high Pearson’s correlation coefficients in both NFHS-4 and NFHS-5. For example, the correlation coefficients for ownership of bank/savings account, knowledge and use of microcredit programme in NFHS-4 were 0.89, 0.85 and 0.92, respectively. the correlation coefficients for ownership of bank/savings account, knowledge and use of microcredit programme in NFHS-4 were 0.80, 0.85 and 0.91, respectively. Moreover, model diagnostic and diagnostic for the small-area estimates revealed that our small-area estimates are consistent, valid and reliable.

A key limitation of the study is that the information on ownership of a bank/savings account, and knowledge and use of microcredit programme in NFHS-4 and NFHS-5 were collected only from women age 15–49. So, our estimates of the afore-mentioned indicators will be lower than the prevalence of these indicators among all women. Second, the information on ownership of a bank/savings account and knowledge and use of microcredit programme is based on women’s self-report, and are thus vulnerable to reporting bias. Third, we could not examine whether women used the bank/savings accounts in the recent past due to unavailability of such information in the two survey rounds. The sixth round of NFHS, which is in the field now, includes a question on whether the woman herself put money in or take money out of the account in the last 12 months. This information may help future researchers to examine what proportion of women who owned an account actually used it in the recent past. Fourth, SAE estimation results may get affected by the choice of auxiliary variables. We tried a number of combinations of available auxiliary variables for generating our estimates. We got the best model fit with the set of auxiliary variables used in the original analyses. Additionally, due to the unavailability of a recent census, we borrowed auxiliary variables from the 2011 Indian Census. This should not be a problem given that both NFHS-4 and NFHS-5 depended on the 2011 Indian Population and Housing Census for their sampling frame. Past Indian studies have borrowed auxiliary variables from the 2011 Indian Census for deriving estimates of gender inequality indicators for the 640 districts of India using NFHS-4 [20,23]. As the 2021 Indian Population and Housing Census could not be conducted due to COVID-19 pandemic, there is no way to examine the impact of selecting auxiliary variables from the 2011 Indian Census on our estimates. Fifth, data collection in NFHS-5 spanned 2019–2021 and was interrupted by COVID-19 lockdowns. As such, approximately 70% of the fieldwork in NFHS-5 was completed before the COVID-19 pandemic hit India and the rest was completed after the COVID-19 related lockdowns were lifted. This may have impacted the increase in women’s ownership of a bank/savings account and use of microcredit programmes. There was no reliable means of estimating the impact of COVID-19 on ownership of a bank/savings account, and use of microcredit programme at the district-level. Despite this limitation, our analysis will help increase targeting of the unbanked women which is increasingly important in the era of DBT. Sixth, the cross-sectional design of the study also limited our ability to derive any causal inferences. Seventh, our estimates refer to the periods 2015–16 and 2019–21; therefore, any changes in women’s financial inclusion indicators occurring after 2021 are not captured in our analysis. The NFHS-6, conducted in 2023–24 across 731 districts of India, is expected to shed light on post-2021 trends and the effects of post-pandemic policy changes on women’s financial inclusion. Despite these limitations, our study provides compelling district-level estimates of indicators of women’s financial inclusion in India. Our study also opens doors for similar analysis in other low- and middle- income countries where surveys are conducted at regular intervals. Additionally, our study shows how data from a household survey may be combined with a national census to derive estimates at local levels.

## Conclusions

Using small-area estimation, this study provides reliable district-level estimates of women’s financial inclusion in India and reveals substantial intrastate variation in both levels and changes over time. Spatial analyses further show distinct clustering patterns, indicating persistent geographic inequalities. These findings highlight the need for more granular monitoring of financial inclusion progress beyond state averages.. These findings have direct relevance for national financial inclusion efforts, including the PMJDY and related programmes that aim to expand women’s access to formal financial services in India. Identifying districts that remain underserved despite national progress can support more efficient targeting and improved implementation of these initiatives. In addition to the direct policy and program applications for women’s financial inclusion, our study opens doors to the exploration of the relationship between women’s financial inclusion and other important health, social and development factors at district level. Future research should consider the application of SAE to estimate the prevalence at regular intervals for monitoring the effectiveness of the policies and programmes interventions meant to enhance women’s financial inclusion and thereby achieving greater gender equality.

## Supporting information

S1 File Estimation of outcome variables. (DOCX)

S2 File Definition of auxiliary variables. (DOCX)

S3 File Diagnostic measures. (DOCX)

S1 Fig Map showing states and union territories of India. (DOCX)

S2 Fig Model diagnostic plot showing the distribution of the district level residuals for women’s ownership of bank/savings account and knowledge and use of microcredit programme in India, NFHS-4 (2015−16) and NFHS-5 (2019−21). (DOCX)

S3 Fig Plots comparing the ordinary least square regression line (dash line) and y = x (solid line), India, NFHS-4 (2015−16) and NFHS-5 (2019−21). (DOCX)

S4 Fig District wise coefficient of variation for women’s ownership of a bank/savings account and knowledge and use of microcredit programme in India, NFHS-4 (2015−16) and NFHS-5 (2019−21). (DOCX)

S5 Fig District wise 95% CI for women’s ownership of a bank/savings account and knowledge and use of microcredit programme in India, NFHS-4 (2015−16) and NFHS-5 (2019−21). (DOCX)

S1 Table Changes in the district boundaries from NFHS-4 (2015−16) to NFHS-5 (2019−21). (DOCX)

S2 Table Within state variation in estimate of women’s ownership of a bank/savings account and knowledge and use of microcredit programme in direct survey-based and model-based estimates in India,NFHS-4 (2015−16) and NFHS-5 (2019−21). (DOCX)

S3 Table Model-based estimates of women’s ownership of a bank/savings account and knowledge and use of microcredit programme in districts of India, NFHS-4 (2015−16) and NFHS-5 (2019−21). (DOCX)

## Abbreviation:

CI: confidence intervals
CV: coefficient of variation
DBT: Direct Benefit Transfer
GLMM: Generalized Linear Mixed Models
LISA: Local Indicator of Spatial Association
NFHS: National Family Health Survey
PMJDY: Pradhan Mantri Jan Dhan Yojana
SAE: Small-area estimation
SES: Socio-economic status
SHG: Self-help group
