# Supplementary material for: Spatial Heterogeneity in Women’s Financial Inclusion in India: An application of small area estimation
Source: PLoS One. 2026 Apr 28;21(4):e0347585. doi: 10.1371/journal.pone.0347585 (PMC13123943; doi:10.1371/journal.pone.0347585)
Supplement: S1 File — (DOCX) [file pone.0347585.s001.docx]

**S1 Text**

**Estimation of outcome variables**

The NFHS-4 and 5 asked the following question to women who were administered state module to capture ownership of a bank/savings account:

1. Do you have a bank or savings account that you yourself use?

Women were coded as ‘owning a bank account’ if they answered ‘yes’ to the above question.

Similarly, the NFHS asked the following questions to women who were administered state module to capture knowledge and use of microcredit programme:

1. Do you know of any programmes in this area that give loans to women to start or expand a business of their own?

Women were coded as ‘being aware of a microcredit programme’ if they answered ‘yes’ to the above question.

1. Have you yourself ever taken a loan, in cash or in kind, from any of these programmes, to start or expand a business?

Women were coded as ‘used microcredit programme’ if they answered ‘yes’ to the above question.

We finally aggregated these three indicators at districts-level to estimate the district-level prevalence of ownership to bank account, knowledge and use of microcredit programme among women.
