# Supplementary material for: Spatial Heterogeneity in Women’s Financial Inclusion in India: An application of small area estimation
Source: PLoS One. 2026 Apr 28;21(4):e0347585. doi: 10.1371/journal.pone.0347585 (PMC13123943; doi:10.1371/journal.pone.0347585)
Supplement: S2 File — (DOCX) [file pone.0347585.s002.docx]

**S2 Text**

**Definition of auxiliary variables**

Auxiliary variables were derived from the 2011 Indian Census (latest available Indian Census). The details of the auxiliary variables are given below:

1. Female workforce participation: The percentage of women age 15-49 who worked as main- or marginal- worker
2. Gender gap relative to male workforce participation: The difference between percentage of male workforce participation and female workforce participation
3. Female literacy: Percentage of women age 15-49 who were literate
4. Gender gap relative to male literacy: The difference between percentage of male and female literates
5. Female headed household: Percentage of households headed by a female member
6. Female mean age at marriage
7. Spousal age gap
8. Birth of a male to female child among women age 30-49 years
9. Mean household size in a district
10. Media exposure (TV/Radio)
11. Urban residence: Percentage of population residing in urban areas
12. Scheduled castes/tribes: Percentage of households belonging to the scheduled castes/tribes
13. Muslim: Percentage of households belonging to Muslim religion
14. Socio-economic status
15. Proportion of village having self-help group in district
16. Proportion of village having any commercial/cooperative bank district

**Estimation of district-level socio-economic status**

To construct district-specific socio-economic status, we used consumer durables (bicycle, motorcycle, car, telephone) and housing characteristics (type of wall, roof, floor, availability of electricity, sources of drinking water, toilet facility, availability of separate kitchen, clean fuel for cooking) [1]. Scores from these items were derived using principal components analysis at district-level, and further categorized into deciles, from lowest to highest; the lowest 10% of districts out of 640 districts are categorized as being in the lowest SES, second 10% of the districts being in the second lowest SES, and the highest 10% of districts are in the highest SES.

**References**

1. Filmer D, Pritchett LH. Estimating wealth effects without expenditure data—or tears: an application to educational enrollments in states of India. Demography. 2001; 38:115-32. doi: <https://doi.org/10.1353/dem.2001.0003>
