# Supplementary material for: Spatial Heterogeneity in Women’s Financial Inclusion in India: An application of small area estimation
Source: PLoS One. 2026 Apr 28;21(4):e0347585. doi: 10.1371/journal.pone.0347585 (PMC13123943; doi:10.1371/journal.pone.0347585)
Supplement: S3 File — (DOCX) [file pone.0347585.s003.docx]

**S3 Text**

**Diagnostic measures**

The model diagnostic is used to verify the assumptions of the underlying model. Under the GLMM framework, the random area specific effects are assumed to follow a normal distribution with mean zero and a constant variance. If the model assumptions are upheld, then the area (district) level residuals are expected to be randomly distributed and not significantly different from the line y=0 [1].

The diagnostics for small-area estimates are used to validate the reliability of model-based small area estimates obtained from GLMM models. These diagnostics include a) bias diagnostic, b) CV, and c) 95% confidence intervals (CIs) of model-based estimates [1]. The bias diagnostic is used to examine the deviation of the model-based district-level estimates from the direct survey estimates to validate the reliability of the model-based district-level estimates. The CVs are used to assess the improvement in the precision of the model-based estimates over the direct survey-based estimates. Estimates with low CV are considered more reliable. The 95% CIs of the model-based estimates and direct survey-based estimates are compared to validate the robustness of the model-based estimates.

**References**

1. Pfeffermann D, Sverchkov M. Small-area estimation under informative probability sampling of areas and within the selected areas. Journal of the American Statistical Association. 2007; 102 (480):1427-1439. doi: [10.1198/016214507000001094](https://doi.org/10.1198/016214507000001094)
