# Supplementary material for: Spatial Heterogeneity in Women’s Financial Inclusion in India: An application of small area estimation
Source: PLoS One. 2026 Apr 28;21(4):e0347585. doi: 10.1371/journal.pone.0347585 (PMC13123943; doi:10.1371/journal.pone.0347585)
Supplement: S1 Fig — (DOCX) [file pone.0347585.s004.docx]

| **S1 Fig** Map showing states and union territories of India  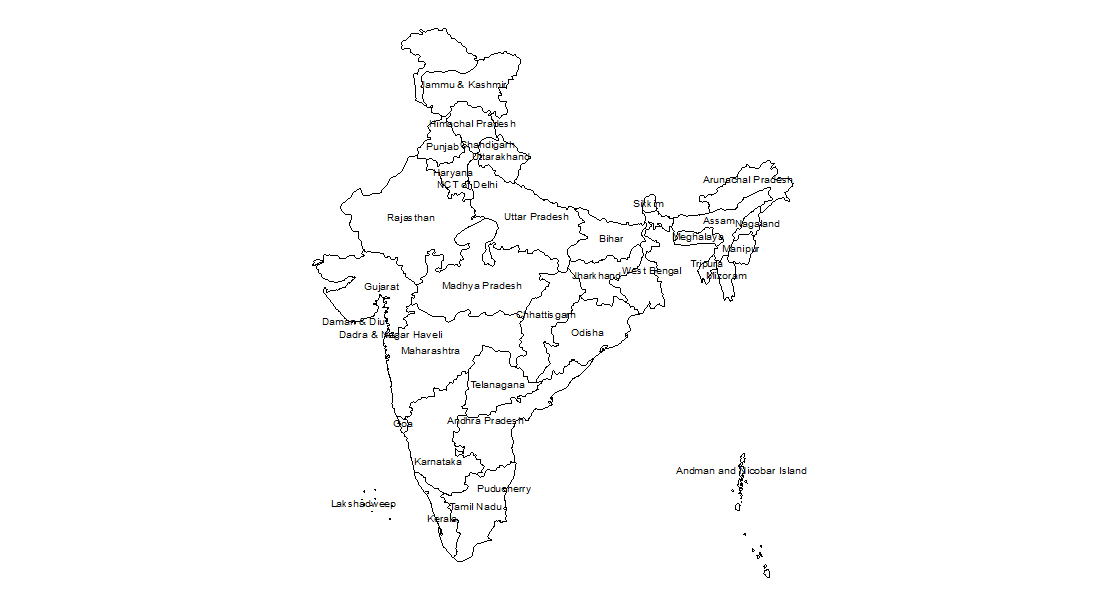 |
| --- |

**Sources:** The shape file downloaded from Spatial Data Repository – Boundaries, The Demographic and Health Surveys Program, ICF International, funded by the United States Agency for International Development (USAID). Available from <https://spatialdata.dhsprogram.com/boundaries/#view=table&countryId=IA> [Accessed 22 February, 2023].
