# Supplementary material for: Spatial Heterogeneity in Women’s Financial Inclusion in India: An application of small area estimation
Source: PLoS One. 2026 Apr 28;21(4):e0347585. doi: 10.1371/journal.pone.0347585 (PMC13123943; doi:10.1371/journal.pone.0347585)
Supplement: S2 Fig — (DOCX) [file pone.0347585.s005.docx]

| **S2 Fig** Model diagnostic plot showing the distribution of the district level residuals for women’s ownership of bank/savings account and knowledge and use of microcredit programme in India, NFHS-4 (2015-16) and NFHS-5 (2019-21) | | |
| --- | --- | --- |
| 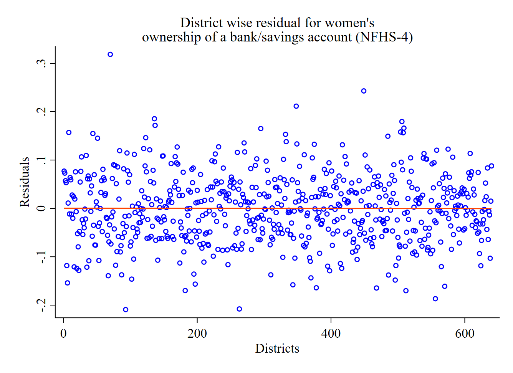 | 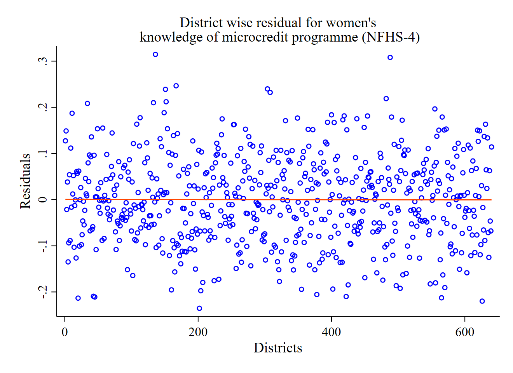 | 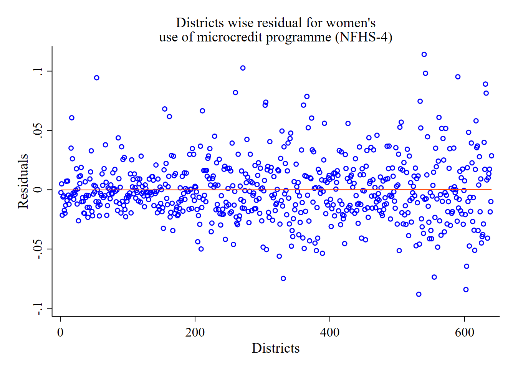 |
| 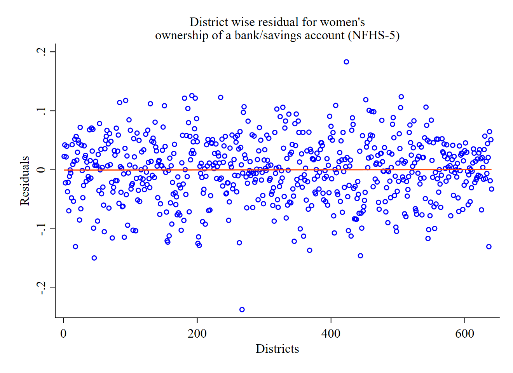 | 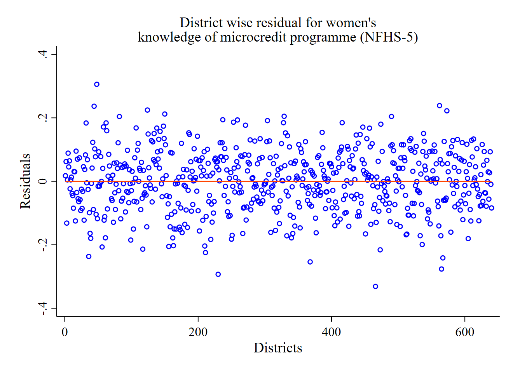 | 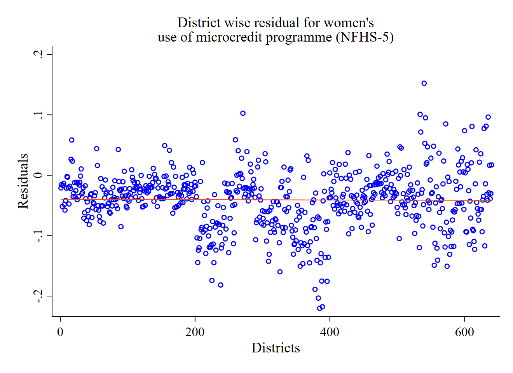 |
| Sources: Author’s calculation from SAE technique. | | |
