# Supplementary material for: Spatial Heterogeneity in Women’s Financial Inclusion in India: An application of small area estimation
Source: PLoS One. 2026 Apr 28;21(4):e0347585. doi: 10.1371/journal.pone.0347585 (PMC13123943; doi:10.1371/journal.pone.0347585)
Supplement: S1 Table — (DOCX) [file pone.0347585.s009.docx]

| **S1 Table** Changes in the district boundaries from NFHS-4 (2015-16) to NFHS-5 (2019-21) | |
| --- | --- |
| **Categories** | **Number of districts** |
| Number of districts in NFHS-4 | 640 |
| Number of districts in NFHS-5 | 707 |
| Districts with unchanged boundaries in NFHS-5 | 585 |
| Districts created by partition of single district in NFHS-5 | 116 |
| Districts created by using parts of the multiple districts in NFHS-5 | 6 |
| Sources: Author’s calculation based on the Census of India-2011, NFHS-4 and NFHS-5 datasets. | |
