# Supplementary material for: Spatial Heterogeneity in Women’s Financial Inclusion in India: An application of small area estimation
Source: PLoS One. 2026 Apr 28;21(4):e0347585. doi: 10.1371/journal.pone.0347585 (PMC13123943; doi:10.1371/journal.pone.0347585)
Supplement: S2 Table — (DOCX) [file pone.0347585.s010.docx]

| **S2 Table**  Within state variation in estimate of women's ownership of a bank/savings account and knowledge and use of microcredit programme in direct survey-based and model-based estimates in India,NFHS-4 (2015-16) and NFHS-5 (2019-21) | | | | | | | | | | | | | |
| --- | --- | --- | --- | --- | --- | --- | --- | --- | --- | --- | --- | --- | --- |
| State | No of districts | Ownership of a bank/savings account | | | | Knowledge of microcredit programme | | | | Use of microcredit programme | | | |
|  |  | NFHS-4 | | NFHS-5 | | NFHS-4 | | NFHS-5 | | NFHS-4 | | NFHS-5 | |
|  |  | Direct survey-based CV | Model-based CV | Direct survey-based CV | Model-based CV | Direct survey-based CV | Model-based CV | Direct survey-based CV | Model-based CV | Direct survey-based CV | Model-based CV | Direct survey-based CV | Model-based CV |
| Andaman & Nicobar Islands | 3 | 0.210 | 0.081 | 0.054 | 0.018 | 0.554 | 0.398 | 0.275 | 0.041 | 1.280 | 0.072 | 0.699 | 0.133 |
| Andhra Pradesh | 13 | 0.117 | 0.044 | 0.098 | 0.042 | 0.092 | 0.065 | 0.140 | 0.098 | 0.206 | 0.150 | 0.261 | 0.200 |
| Arunachal Pradesh | 16 | 0.181 | 0.118 | 0.081 | 0.055 | 0.506 | 0.250 | 0.353 | 0.127 | 0.827 | 0.238 | 0.499 | 0.309 |
| Assam | 27 | 0.177 | 0.179 | 0.051 | 0.050 | 0.328 | 0.208 | 0.146 | 0.076 | 0.639 | 0.360 | 0.371 | 0.238 |
| Bihar | 38 | 0.303 | 0.150 | 0.057 | 0.036 | 0.379 | 0.179 | 0.182 | 0.057 | 0.581 | 0.214 | 0.245 | 0.196 |
| Chandigarh | 1 | 0.000 | 0.000 | 0.000 | 0.000 | 0.000 | 0.000 | 0.000 | 0.000 | 0.000 | 0.000 | 0.000 | 0.000 |
| Chhattisgarh | 18 | 0.151 | 0.117 | 0.075 | 0.040 | 0.294 | 0.131 | 0.242 | 0.135 | 0.492 | 0.218 | 0.527 | 0.221 |
| Dadra & Nagar Haveli Daman Diu | 3 | 0.169 | 0.071 | 0.100 | 0.043 | 0.506 | 0.126 | 0.238 | 0.151 | 0.744 | 0.547 | 0.932 | 0.279 |
| Goa | 2 | 0.154 | 0.128 | 0.006 | 0.014 | 0.373 | 0.196 | 0.137 | 0.015 | 0.581 | 0.257 | 0.346 | 0.280 |
| Gujarat | 26 | 0.311 | 0.169 | 0.103 | 0.068 | 0.376 | 0.238 | 0.322 | 0.151 | 0.857 | 0.376 | 0.550 | 0.289 |
| Haryana | 21 | 0.131 | 0.053 | 0.123 | 0.095 | 0.279 | 0.146 | 0.315 | 0.173 | 0.632 | 0.249 | 0.749 | 0.254 |
| Himanchal Pradesh | 12 | 0.208 | 0.123 | 0.070 | 0.027 | 0.355 | 0.200 | 0.205 | 0.091 | 1.222 | 0.307 | 0.614 | 0.161 |
| Jammu & Kashmir | 21 | 0.178 | 0.143 | 0.072 | 0.039 | 0.244 | 0.123 | 0.287 | 0.212 | 0.630 | 0.249 | 1.117 | 0.392 |
| Jharkhand | 24 | 0.215 | 0.145 | 0.092 | 0.033 | 0.354 | 0.140 | 0.134 | 0.071 | 0.459 | 0.288 | 0.390 | 0.220 |
| Karnataka | 30 | 0.097 | 0.066 | 0.063 | 0.036 | 0.143 | 0.068 | 0.242 | 0.084 | 0.396 | 0.334 | 0.375 | 0.242 |
| Kerala | 14 | 0.000 | 0.000 | 0.060 | 0.044 | 0.000 | 0.000 | 0.147 | 0.090 | 0.000 | 0.000 | 0.320 | 0.228 |
| Lakshadweep | 1 | 0.238 | 0.166 | 0.000 | 0.000 | 0.312 | 0.182 | 0.000 | 0.000 | 0.596 | 0.252 | 0.000 | 0.000 |
| Madhya Pradesh | 50 | 0.272 | 0.112 | 0.100 | 0.046 | 0.304 | 0.117 | 0.273 | 0.120 | 0.533 | 0.336 | 0.505 | 0.242 |
| Maharashtra | 35 | 0.145 | 0.113 | 0.106 | 0.063 | 0.408 | 0.211 | 0.237 | 0.114 | 0.661 | 0.293 | 0.419 | 0.310 |
| Manipur | 9 | 0.116 | 0.077 | 0.049 | 0.049 | 0.608 | 0.212 | 0.363 | 0.162 | 0.661 | 0.264 | 0.619 | 0.232 |
| Meghalaya | 7 | 0.189 | 0.182 | 0.101 | 0.067 | 0.454 | 0.202 | 0.362 | 0.144 | 0.900 | 0.300 | 0.335 | 0.304 |
| Mizoram | 8 | 0.408 | 0.263 | 0.066 | 0.044 | 0.547 | 0.272 | 0.446 | 0.171 | 0.972 | 0.276 | 0.848 | 0.371 |
| Nagaland | 11 | 0.229 | 0.151 | 0.211 | 0.105 | 0.386 | 0.137 | 0.484 | 0.153 | 0.729 | 0.509 | 0.762 | 0.266 |
| NCT Of Delhi | 9 | 0.155 | 0.074 | 0.104 | 0.035 | 0.146 | 0.071 | 0.222 | 0.089 | 0.263 | 0.197 | 0.515 | 0.258 |
| Odisha | 30 | 0.071 | 0.070 | 0.053 | 0.028 | 0.173 | 0.142 | 0.105 | 0.053 | 0.626 | 0.568 | 0.286 | 0.193 |
| Puducherry | 4 | 0.157 | 0.070 | 0.113 | 0.024 | 0.273 | 0.159 | 0.100 | 0.060 | 1.019 | 0.273 | 0.334 | 0.207 |
| Punjab | 20 | 0.150 | 0.096 | 0.090 | 0.051 | 0.363 | 0.228 | 0.296 | 0.098 | 0.693 | 0.340 | 0.461 | 0.204 |
| Rajasthan | 33 | 0.086 | 0.050 | 0.065 | 0.035 | 0.200 | 0.134 | 0.235 | 0.158 | 0.545 | 0.072 | 0.641 | 0.347 |
| Sikkim | 4 | 0.064 | 0.046 | 0.070 | 0.023 | 0.200 | 0.087 | 0.208 | 0.120 | 0.358 | 0.188 | 0.440 | 0.272 |
| Tamil Nadu | 32 | 0.139 | 0.039 | 0.035 | 0.017 | 0.068 | 0.062 | 0.110 | 0.046 | 0.301 | 0.179 | 0.262 | 0.190 |
| Telangana | 10 | 0.161 | 0.118 | 0.027 | 0.038 | 0.477 | 0.222 | 0.197 | 0.061 | 0.834 | 0.294 | 0.355 | 0.266 |
| Tripura | 4 | 0.168 | 0.085 | 0.021 | 0.038 | 0.356 | 0.171 | 0.129 | 0.071 | 0.622 | 0.191 | 0.175 | 0.083 |
| Uttar Pradesh | 71 | 0.199 | 0.169 | 0.103 | 0.048 | 0.235 | 0.126 | 0.324 | 0.126 | 0.448 | 0.287 | 0.583 | 0.204 |
| Uttarakhand | 13 | 0.124 | 0.065 | 0.071 | 0.042 | 0.150 | 0.114 | 0.295 | 0.089 | 0.454 | 0.302 | 0.449 | 0.208 |
| West Bengal | 19 | 0.199 | 0.171 | 0.106 | 0.053 | 0.235 | 0.126 | 0.232 | 0.100 | 0.448 | 0.253 | 0.425 | 0.224 |
| Sources: Author’s calculation based on the NFHS-4 and NFHS-5 datasets. | | | | | | | | | | | | | |
