# Supplementary material for: Spatial Heterogeneity in Women’s Financial Inclusion in India: An application of small area estimation
Source: PLoS One. 2026 Apr 28;21(4):e0347585. doi: 10.1371/journal.pone.0347585 (PMC13123943; doi:10.1371/journal.pone.0347585)
Supplement: S3 Table — (DOCX) [file pone.0347585.s011.docx]

| **S3 Table** : Model-based estimates of women's ownership of a bank/savings account and knowledge and use of microcredit programme in districts of India, NFHS-4 (2015-16) and NFHS-5 (2019-21) | | | | | | | | | | |
| --- | --- | --- | --- | --- | --- | --- | --- | --- | --- | --- |
| State name | District name | Ownership of bank/saving account that they themselves use | | | Knowledge of a microcredit programme | | | Use of a microcredit programme | | |
|  |  | NFHS-4 | NFHS-5 | Changes between NFHS-4 and NFHS-5 | NFHS-4 | NFHS-5 | Changes between NFHS-4 and NFHS-5 | NFHS-4 | NFHS-5 | Changes between NFHS-4 and NFHS-5 |
|  |  | Estimates (Lower bound, Upper bound) | Estimates (Lower bound, Upper bound) |  | Estimates (Lower bound, Upper bound) | Estimates (Lower bound, Upper bound) |  | Estimates (Lower bound, Upper bound) | Estimates (Lower bound, Upper bound) |  |
| Jammu & Kashmir | Kupwara | 52.5(51.7,53.3) | 81.6(80.9,82.2) | 29.1 | 31.7(31.0,32.5) | 21.4(20.7,22.1) | -10.3 | 1.7(1.4,1.9) | 3.5(3.2,3.8) | 1.9 |
| Jammu & Kashmir | Budgam | 60.2(59.3,61.0) | 85.0(84.4,85.6) | 24.9 | 41.1(40.3,41.9) | 22.3(21.6,23.0) | -18.8 | 1.5(1.3,1.7) | 3.6(3.3,3.9) | 2.0 |
| Jammu & Kashmir | Leh (Ladakh) | 78.4(76.8,80.0) | 90.7(89.6,91.8) | 12.3 | 50.8(48.8,52.7) | 31.5(29.8,33.3) | -19.2 | 2.4(1.8,3.0) | 5.5(4.6,6.3) | 3.0 |
| Jammu & Kashmir | Kargil | 63.3(61.5,65.2) | 86.1(84.8,87.4) | 22.8 | 27.7(26.0,29.4) | 18.2(16.7,19.6) | -9.6 | 0.6(0.3,0.9) | 1.7(1.3,2.2) | 1.1 |
| Jammu & Kashmir | Poonch | 51.9(50.9,52.9) | 78.9(78.1,79.7) | 27.0 | 29.0(28.0,29.9) | 14.2(13.5,14.9) | -14.8 | 1.5(1.3,1.8) | 2.3(2.0,2.6) | 0.8 |
| Jammu & Kashmir | Rajouri | 49.8(48.9,50.7) | 82.2(81.6,82.9) | 32.5 | 29.1(28.3,29.9) | 17.2(16.6,17.9) | -11.9 | 1.7(1.5,2.0) | 4.8(4.5,5.2) | 3.1 |
| Jammu & Kashmir | Kathua | 62.9(62.0,63.7) | 83.2(82.5,83.8) | 20.3 | 48.2(47.3,49.0) | 18.7(18.1,19.4) | -29.4 | 2.3(2.1,2.6) | 6.1(5.7,6.5) | 3.8 |
| Jammu & Kashmir | Baramulla | 57.1(56.4,57.8) | 85.0(84.5,85.5) | 27.9 | 30.5(29.9,31.1) | 26.4(25.8,27.0) | -4.1 | 1.3(1.2,1.5) | 4.2(3.9,4.5) | 2.9 |
| Jammu & Kashmir | Bandipora | 59.8(58.7,60.9) | 86.3(85.5,87.1) | 26.5 | 38.9(37.8,40.0) | 23.6(22.6,24.5) | -15.4 | 1.0(0.8,1.3) | 3.9(3.4,4.3) | 2.8 |
| Jammu & Kashmir | Srinagar | 73.2(72.7,73.8) | 90.1(89.7,90.4) | 16.8 | 51.8(51.3,52.4) | 18.4(17.9,18.9) | -33.5 | 2.2(2.0,2.3) | 1.5(1.4,1.6) | -0.7 |
| Jammu & Kashmir | Ganderbal | 59.5(58.2,60.8) | 86.3(85.4,87.2) | 26.8 | 33.7(32.5,35.0) | 21.9(20.9,23.0) | -11.8 | 1.5(1.2,1.8) | 2.5(2.1,2.9) | 1.0 |
| Jammu & Kashmir | Pulwama | 63.0(62.1,63.9) | 84.3(83.6,84.9) | 21.3 | 39.6(38.7,40.5) | 21.5(20.8,22.3) | -18.0 | 1.3(1.0,1.5) | 2.8(2.5,3.1) | 1.6 |
| Jammu & Kashmir | Shopian | 61.9(60.6,63.1) | 87.1(86.2,88.0) | 25.2 | 31.5(30.3,32.7) | 30.4(29.2,31.6) | -1.1 | 1.6(1.2,1.9) | 5.1(4.5,5.7) | 3.5 |
| Jammu & Kashmir | Anantnag | 66.6(66.0,67.3) | 82.1(81.6,82.6) | 15.5 | 39.7(39.0,40.3) | 15.3(14.8,15.8) | -24.3 | 0.9(0.8,1.0) | 2.0(1.8,2.2) | 1.1 |
| Jammu & Kashmir | Kulgam | 60.6(59.6,61.7) | 84.1(83.4,84.9) | 23.5 | 34.7(33.7,35.7) | 21.4(20.5,22.2) | -13.3 | 1.1(0.9,1.3) | 2.7(2.3,3.0) | 1.6 |
| Jammu & Kashmir | Doda | 54.0(52.9,55.1) | 86.7(85.9,87.4) | 32.6 | 28.3(27.3,29.3) | 17.4(16.6,18.3) | -10.9 | 1.4(1.1,1.6) | 2.2(1.9,2.5) | 0.8 |
| Jammu & Kashmir | Ramban | 44.9(43.6,46.3) | 83.8(82.8,84.8) | 38.9 | 40.0(38.6,41.3) | 18.0(16.9,19.0) | -22.0 | 2.2(1.8,2.6) | 2.4(2.0,2.8) | 0.2 |
| Jammu & Kashmir | Kishtwar | 55.2(53.7,56.6) | 78.6(77.4,79.8) | 23.4 | 32.1(30.8,33.5) | 18.7(17.5,19.8) | -13.4 | 2.1(1.7,2.5) | 2.3(1.9,2.8) | 0.3 |
| Jammu & Kashmir | Udhampur | 58.8(57.9,59.7) | 81.7(81.0,82.4) | 22.9 | 48.6(47.6,49.5) | 19.0(18.3,19.8) | -29.5 | 2.0(1.8,2.3) | 5.3(4.9,5.7) | 3.3 |
| Jammu & Kashmir | Reasi | 56.1(54.8,57.4) | 81.2(80.2,82.2) | 25.1 | 34.8(33.6,36.1) | 16.2(15.2,17.1) | -18.7 | 1.4(1.1,1.7) | 3.5(3.1,4.0) | 2.2 |
| Jammu & Kashmir | Jammu | 61.1(60.6,61.6) | 90.0(89.7,90.3) | 28.9 | 36.0(35.5,36.5) | 20.5(20.0,20.9) | -15.5 | 2.2(2.0,2.3) | 3.2(3.0,3.3) | 1.0 |
| Jammu & Kashmir | Samba | 63.0(61.9,64.1) | 85.0(84.1,85.8) | 22.0 | 38.5(37.4,39.6) | 21.8(20.9,22.8) | -16.7 | 2.1(1.8,2.4) | 5.3(4.8,5.8) | 3.2 |
| Himanchal Pradesh | Chamba | 68.9(68.1,69.8) | 81.4(80.7,82.1) | 12.4 | 30.9(30.0,31.7) | 42.3(41.4,43.2) | 11.5 | 1.8(1.6,2.0) | 4.3(3.9,4.7) | 2.5 |
| Himanchal Pradesh | Kangra | 71.1(70.6,71.5) | 83.1(82.7,83.5) | 12.0 | 39.1(38.6,39.6) | 49.9(49.4,50.4) | 10.8 | 1.9(1.7,2.0) | 4.8(4.6,5.1) | 3.0 |
| Himanchal Pradesh | Lahul and Spiti | 71.4(68.1,74.7) | 87.3(84.9,89.8) | 15.9 | 26.7(23.5,29.9) | 62.2(58.7,65.8) | 35.6 | 2.4(1.3,3.5) | 5.2(3.6,6.8) | 2.8 |
| Himanchal Pradesh | Kullu | 69.7(68.8,70.6) | 84.7(84.0,85.4) | 15.1 | 37.2(36.2,38.1) | 50.5(49.6,51.5) | 13.4 | 2.2(1.9,2.5) | 3.6(3.3,4.0) | 1.4 |
| Himanchal Pradesh | Mandi | 72.5(72.0,73.1) | 87.0(86.6,87.4) | 14.5 | 36.0(35.4,36.6) | 49.3(48.6,49.9) | 13.2 | 2.6(2.4,2.8) | 3.5(3.3,3.8) | 0.9 |
| Himanchal Pradesh | Hamirpur | 75.3(74.5,76.1) | 86.5(85.9,87.1) | 11.2 | 26.6(25.8,27.4) | 48.7(47.8,49.6) | 22.1 | 1.8(1.6,2.0) | 4.5(4.2,4.9) | 2.7 |
| Himanchal Pradesh | Una | 66.6(65.8,67.4) | 81.3(80.7,82.0) | 14.8 | 35.9(35.0,36.7) | 47.8(46.9,48.7) | 11.9 | 1.5(1.3,1.7) | 3.2(2.9,3.5) | 1.7 |
| Himanchal Pradesh | Bilaspur | 66.7(65.7,67.6) | 85.5(84.7,86.2) | 18.8 | 29.3(28.4,30.2) | 48.3(47.3,49.4) | 19.1 | 1.6(1.4,1.9) | 2.9(2.6,3.3) | 1.3 |
| Himanchal Pradesh | Solan | 68.7(67.9,69.5) | 85.3(84.7,85.9) | 16.7 | 32.9(32.1,33.7) | 52.2(51.3,53.0) | 19.3 | 3.3(3.0,3.6) | 4.1(3.8,4.4) | 0.8 |
| Himanchal Pradesh | Sirmaur | 62.1(61.3,63.0) | 83.4(82.7,84.1) | 21.3 | 25.6(24.8,26.4) | 49.0(48.1,50.0) | 23.5 | 1.9(1.6,2.1) | 4.0(3.6,4.3) | 2.1 |
| Himanchal Pradesh | Shimla | 72.6(72.0,73.2) | 83.1(82.5,83.6) | 10.5 | 28.2(27.6,28.9) | 51.0(50.3,51.7) | 22.8 | 2.0(1.8,2.2) | 4.2(3.9,4.5) | 2.2 |
| Himanchal Pradesh | Kinnaur | 74.2(72.2,76.2) | 88.1(86.6,89.6) | 13.9 | 31.3(29.2,33.5) | 53.0(50.7,55.3) | 21.7 | 2.9(2.2,3.7) | 4.0(3.1,4.9) | 1.1 |
| Punjab | Gurdaspur | 60.0(59.6,60.5) | 83.0(82.7,83.3) | 23.0 | 51.1(50.7,51.5) | 41.8(41.4,42.3) | -9.2 | 3.7(3.5,3.9) | 8.9(8.6,9.1) | 5.2 |
| Punjab | Kapurthala | 59.7(59.0,60.4) | 82.4(81.9,83.0) | 22.7 | 57.5(56.8,58.2) | 52.5(51.8,53.2) | -5.0 | 3.2(3.0,3.5) | 7.5(7.1,7.8) | 4.2 |
| Punjab | Jalandhar | 68.3(67.9,68.7) | 85.5(85.2,85.8) | 17.2 | 40.9(40.5,41.3) | 43.9(43.4,44.3) | 3.0 | 2.0(1.9,2.1) | 4.4(4.2,4.6) | 2.4 |
| Punjab | Hoshiarpur | 62.4(61.9,62.9) | 90.3(90.0,90.6) | 27.9 | 36.1(35.6,36.6) | 43.4(42.9,43.9) | 7.3 | 2.3(2.2,2.5) | 5.5(5.3,5.8) | 3.2 |
| Punjab | Sangrur | 66.6(66.1,67.1) | 79.8(79.4,80.2) | 13.2 | 47.1(46.6,47.6) | 44.6(44.1,45.1) | -2.5 | 2.0(1.8,2.1) | 9.0(8.7,9.3) | 7.0 |
| Punjab | Fatehgarh Sahib | 57.7(56.8,58.5) | 87.0(86.4,87.6) | 29.3 | 37.1(36.2,37.9) | 39.2(38.3,40.0) | 2.1 | 2.3(2.0,2.5) | 5.2(4.8,5.6) | 2.9 |
| Punjab | Ludhiana | 58.4(58.0,58.7) | 79.9(79.6,80.2) | 21.5 | 59.0(58.6,59.3) | 47.6(47.2,47.9) | -11.4 | 3.3(3.2,3.4) | 6.8(6.6,7.0) | 3.5 |
| Punjab | Moga | 56.9(56.2,57.5) | 82.0(81.5,82.5) | 25.1 | 56.0(55.4,56.6) | 39.5(38.9,40.1) | -16.5 | 2.9(2.6,3.1) | 9.2(8.8,9.6) | 6.3 |
| Punjab | Ferozepur | 55.5(55.1,56.0) | 80.7(80.3,81.1) | 25.2 | 43.7(43.3,44.2) | 42.2(41.7,42.7) | -1.5 | 2.9(2.7,3.0) | 9.5(9.3,9.8) | 6.7 |
| Punjab | Sri Muktsar Sahib | 54.2(53.5,54.9) | 79.0(78.5,79.6) | 24.9 | 43.6(42.9,44.2) | 41.5(40.9,42.2) | -2.0 | 2.0(1.8,2.1) | 6.6(6.3,7.0) | 4.7 |
| Punjab | Faridkot | 58.7(57.8,59.5) | 77.8(77.1,78.5) | 19.2 | 44.2(43.4,45.0) | 40.1(39.3,40.9) | -4.1 | 3.5(3.2,3.8) | 8.2(7.8,8.7) | 4.7 |
| Punjab | Bathinda | 59.2(58.6,59.7) | 75.2(74.7,75.7) | 16.0 | 41.2(40.7,41.8) | 38.0(37.5,38.5) | -3.2 | 1.9(1.8,2.1) | 8.3(8.0,8.6) | 6.3 |
| Punjab | Mansa | 54.6(53.8,55.3) | 79.0(78.4,79.6) | 24.5 | 47.5(46.8,48.2) | 42.3(41.6,43.0) | -5.2 | 2.5(2.3,2.7) | 7.6(7.3,8.0) | 5.2 |
| Punjab | Patiala | 56.4(56.0,56.9) | 86.0(85.6,86.3) | 29.5 | 39.0(38.6,39.5) | 39.3(38.9,39.8) | 0.3 | 2.2(2.1,2.3) | 5.4(5.2,5.6) | 3.2 |
| Punjab | Amritsar | 62.9(62.5,63.3) | 80.1(79.8,80.4) | 17.2 | 52.3(51.9,52.7) | 39.9(39.5,40.3) | -12.4 | 4.1(3.9,4.3) | 8.3(8.0,8.5) | 4.2 |
| Punjab | Taran Taran | 58.3(57.7,58.9) | 78.5(78.0,79.0) | 20.2 | 55.0(54.4,55.6) | 46.8(46.2,47.4) | -8.2 | 2.2(2.1,2.4) | 7.3(7.0,7.6) | 5.1 |
| Punjab | Rupnagar | 59.0(58.2,59.7) | 88.8(88.3,89.3) | 29.8 | 35.9(35.2,36.7) | 39.8(39.0,40.5) | 3.8 | 2.3(2.1,2.5) | 5.5(5.2,5.9) | 3.2 |
| Punjab | S.A.S Nagar | 68.1(67.5,68.7) | 87.0(86.6,87.5) | 18.9 | 51.4(50.8,52.1) | 37.0(36.4,37.6) | -14.4 | 4.2(3.9,4.4) | 7.4(7.0,7.7) | 3.2 |
| Punjab | Shahid Bhagat Singh Nagar | 56.6(55.8,57.4) | 78.8(78.1,79.5) | 22.2 | 38.8(38.0,39.6) | 33.9(33.1,34.7) | -4.9 | 2.2(1.9,2.4) | 6.8(6.4,7.2) | 4.7 |
| Punjab | Barnala | 57.3(56.4,58.1) | 77.5(76.8,78.2) | 20.2 | 43.8(42.9,44.6) | 40.2(39.4,41.1) | -3.5 | 3.4(3.1,3.7) | 8.5(8.0,9.0) | 5.1 |
| Chandigarh | Chandigarh | 79.6(79.1,80.1) | 87.1(86.7,87.5) | 7.5 | 48.0(47.4,48.7) | 23.6(23.0,24.1) | -24.5 | 2.3(2.1,2.5) | 0.7(0.6,0.8) | -1.6 |
| Uttarakhand | Uttar Kashi | 55.8(54.7,57.0) | 77.3(76.3,78.3) | 21.5 | 20.6(19.7,21.6) | 47.7(46.6,48.9) | 27.1 | 2.7(2.3,3.1) | 6.3(5.7,6.8) | 3.6 |
| Uttarakhand | Chamoli | 65.6(64.6,66.5) | 81.8(81.0,82.6) | 16.3 | 24.4(23.5,25.3) | 52.2(51.2,53.3) | 27.8 | 3.3(2.9,3.7) | 8.1(7.5,8.6) | 4.8 |
| Uttarakhand | Rudra Prayag | 64.7(63.5,65.9) | 86.8(85.9,87.7) | 22.1 | 20.1(19.0,21.1) | 55.9(54.6,57.1) | 35.8 | 2.8(2.4,3.3) | 7.5(6.8,8.2) | 4.6 |
| Uttarakhand | Tehri Garhwal | 67.2(66.4,68.0) | 81.8(81.2,82.5) | 14.7 | 20.0(19.3,20.6) | 44.9(44.1,45.7) | 24.9 | 2.1(1.9,2.4) | 8.5(8.0,9.0) | 6.4 |
| Uttarakhand | Dehradun | 60.5(60.0,61.0) | 88.2(87.9,88.5) | 27.7 | 25.7(25.2,26.1) | 45.8(45.3,46.3) | 20.2 | 3.8(3.6,4.0) | 4.0(3.8,4.2) | 0.2 |
| Uttarakhand | Pauri Garhwal | 66.6(65.8,67.3) | 85.1(84.5,85.6) | 18.5 | 19.2(18.6,19.8) | 51.7(50.9,52.4) | 32.4 | 2.8(2.5,3.0) | 7.4(7.0,7.8) | 4.6 |
| Uttarakhand | Pithoragarh | 61.3(60.4,62.2) | 79.8(79.1,80.6) | 18.5 | 19.4(18.7,20.1) | 48.0(47.0,48.9) | 28.6 | 2.5(2.2,2.8) | 5.7(5.3,6.1) | 3.2 |
| Uttarakhand | Bageshwar | 64.1(62.9,65.3) | 83.8(82.9,84.7) | 19.7 | 21.1(20.1,22.1) | 53.5(52.3,54.7) | 32.4 | 3.3(2.9,3.8) | 8.8(8.1,9.5) | 5.5 |
| Uttarakhand | Almora | 65.8(65.1,66.6) | 84.2(83.7,84.8) | 18.4 | 23.7(23.0,24.3) | 58.0(57.2,58.7) | 34.3 | 2.0(1.8,2.3) | 9.3(8.8,9.8) | 7.3 |
| Uttarakhand | Champawat | 58.1(56.8,59.4) | 80.7(79.7,81.7) | 22.6 | 22.2(21.1,23.3) | 51.3(50.0,52.6) | 29.1 | 3.3(2.8,3.7) | 7.4(6.7,8.1) | 4.2 |
| Uttarakhand | Nainital | 58.4(57.8,59.1) | 80.6(80.1,81.2) | 22.2 | 26.7(26.1,27.3) | 45.2(44.5,45.9) | 18.5 | 2.9(2.7,3.1) | 6.1(5.8,6.5) | 3.2 |
| Uttarakhand | Udam Singh Nagar | 57.2(56.7,57.8) | 79.9(79.5,80.4) | 22.7 | 31.9(31.4,32.4) | 57.5(57.0,58.1) | 25.6 | 3.0(2.8,3.2) | 7.6(7.3,7.9) | 4.6 |
| Uttarakhand | Haridwar | 49.7(49.2,50.2) | 76.5(76.0,76.9) | 26.8 | 17.7(17.4,18.1) | 49.2(48.7,49.7) | 31.5 | 2.0(1.9,2.2) | 5.7(5.5,5.9) | 3.7 |
| Haryana | Panchkula | 55.0(54.1,55.8) | 86.7(86.1,87.3) | 31.7 | 28.4(27.6,29.2) | 31.5(30.7,32.3) | 3.1 | 3.7(3.4,4.0) | 4.3(4.0,4.7) | 0.6 |
| Haryana | Ambala | 54.9(54.2,55.5) | 72.8(72.2,73.3) | 17.9 | 26.2(25.7,26.8) | 31.0(30.4,31.5) | 4.7 | 2.0(1.8,2.1) | 5.8(5.5,6.1) | 3.9 |
| Haryana | Yamunanagar | 52.8(52.2,53.4) | 68.9(68.3,69.4) | 16.1 | 30.2(29.6,30.7) | 32.3(31.7,32.8) | 2.1 | 1.7(1.6,1.9) | 6.6(6.3,6.9) | 4.9 |
| Haryana | Kurukshetra | 51.0(50.3,51.6) | 68.5(67.9,69.1) | 17.5 | 30.3(29.7,30.9) | 33.3(32.7,33.9) | 3.0 | 1.5(1.4,1.7) | 5.7(5.4,6.0) | 4.2 |
| Haryana | Kaithal | 49.2(48.6,49.9) | 78.3(77.7,78.8) | 29.0 | 36.9(36.3,37.5) | 45.5(44.9,46.1) | 8.6 | 2.6(2.4,2.8) | 8.7(8.4,9.1) | 6.2 |
| Haryana | Karnal | 52.1(51.6,52.7) | 72.6(72.1,73.1) | 20.5 | 21.3(20.9,21.8) | 34.8(34.3,35.3) | 13.4 | 1.6(1.4,1.7) | 7.1(6.8,7.3) | 5.5 |
| Haryana | Panipat | 40.1(39.5,40.7) | 72.5(71.9,73.1) | 32.4 | 30.0(29.5,30.6) | 29.4(28.9,30.0) | -0.6 | 2.4(2.2,2.5) | 5.7(5.4,5.9) | 3.3 |
| Haryana | Sonipat | 41.8(41.3,42.4) | 73.9(73.4,74.4) | 32.1 | 22.0(21.6,22.5) | 33.8(33.3,34.3) | 11.8 | 1.4(1.3,1.6) | 5.2(4.9,5.4) | 3.7 |
| Haryana | Jind | 47.2(46.6,47.8) | 70.4(69.8,70.9) | 23.2 | 25.7(25.2,26.2) | 34.5(34.0,35.1) | 8.8 | 1.1(0.9,1.2) | 5.8(5.5,6.1) | 4.7 |
| Haryana | Fatehabad | 47.0(46.3,47.7) | 72.9(72.3,73.5) | 25.9 | 25.6(25.0,26.2) | 37.3(36.6,37.9) | 11.6 | 1.6(1.4,1.8) | 5.0(4.7,5.3) | 3.4 |
| Haryana | Sirsa | 47.5(47.0,48.1) | 80.3(79.8,80.7) | 32.7 | 23.6(23.1,24.0) | 39.6(39.0,40.2) | 16.0 | 2.1(2.0,2.3) | 6.2(5.9,6.5) | 4.1 |
| Haryana | Hisar | 47.1(46.6,47.6) | 80.3(79.9,80.7) | 33.2 | 23.2(22.8,23.7) | 38.2(37.7,38.7) | 15.0 | 1.9(1.8,2.1) | 5.7(5.4,5.9) | 3.7 |
| Haryana | Bhiwani | 45.4(44.9,45.9) | 76.3(75.9,76.8) | 30.9 | 22.2(21.7,22.6) | 36.3(35.8,36.8) | 14.2 | 2.5(2.3,2.6) | 5.1(4.9,5.3) | 2.6 |
| Haryana | Rohtak | 47.5(46.8,48.1) | 76.7(76.2,77.2) | 29.3 | 25.3(24.8,25.9) | 34.6(34.0,35.2) | 9.3 | 2.1(1.9,2.3) | 6.0(5.7,6.3) | 3.9 |
| Haryana | Jhajjar | 42.2(41.5,42.9) | 76.9(76.4,77.5) | 34.7 | 27.5(26.9,28.1) | 31.2(30.6,31.9) | 3.7 | 2.2(2.0,2.4) | 6.3(5.9,6.6) | 4.1 |
| Haryana | Mahendragarh | 40.6(39.9,41.2) | 69.7(69.1,70.3) | 29.2 | 18.8(18.3,19.3) | 26.3(25.7,26.9) | 7.5 | 1.6(1.4,1.8) | 5.4(5.1,5.7) | 3.8 |
| Haryana | Rewari | 48.2(47.5,48.9) | 80.2(79.7,80.8) | 32.0 | 26.1(25.5,26.7) | 32.6(32.0,33.3) | 6.5 | 2.3(2.1,2.5) | 6.0(5.7,6.4) | 3.8 |
| Haryana | Gurugram | 52.8(52.3,53.3) | 85.6(85.3,86.0) | 32.8 | 28.6(28.1,29.1) | 30.9(30.4,31.4) | 2.3 | 3.7(3.5,3.9) | 3.8(3.6,4.0) | 0.1 |
| Haryana | Mewat | 21.9(21.3,22.5) | 57.2(56.5,57.9) | 35.3 | 8.4(8.0,8.8) | 17.9(17.3,18.4) | 9.4 | 0.9(0.8,1.1) | 2.9(2.6,3.1) | 2.0 |
| Haryana | Faridabad | 50.3(49.8,50.8) | 75.4(75.0,75.8) | 25.1 | 20.0(19.6,20.4) | 31.5(31.0,31.9) | 11.5 | 1.5(1.4,1.6) | 2.8(2.6,3.0) | 1.3 |
| Haryana | Palwal | 32.6(32.0,33.3) | 61.0(60.4,61.7) | 28.4 | 17.5(17.0,18.0) | 24.4(23.8,24.9) | 6.8 | 1.0(0.9,1.2) | 3.8(3.5,4.1) | 2.8 |
| NCT Of Delhi | North West Delhi | 55.3(55.0,55.7) | 71.7(71.3,72.0) | 16.3 | 22.3(22.0,22.6) | 36.3(35.9,36.6) | 13.9 | 2.0(1.9,2.1) | 8.5(8.3,8.7) | 6.6 |
| NCT Of Delhi | North Delhi District | 59.8(59.1,60.5) | 73.2(72.6,73.8) | 13.4 | 22.3(21.7,22.8) | 36.2(35.5,36.8) | 13.9 | 2.5(2.3,2.7) | 8.4(8.0,8.8) | 5.9 |
| NCT Of Delhi | North East Delhi | 44.7(44.3,45.2) | 73.5(73.1,73.9) | 28.8 | 21.9(21.5,22.3) | 30.6(30.2,31.1) | 8.7 | 1.9(1.8,2.0) | 4.4(4.2,4.6) | 2.5 |
| NCT Of Delhi | East Delhi | 64.7(64.2,65.1) | 68.8(68.3,69.2) | 4.1 | 18.5(18.2,18.9) | 32.0(31.5,32.5) | 13.5 | 2.6(2.5,2.8) | 6.2(6.0,6.5) | 3.6 |
| NCT Of Delhi | New Delhi | 72.0(70.5,73.6) | 70.8(69.2,72.4) | -1.3 | 18.7(17.4,20.1) | 37.0(35.3,38.7) | 18.3 | 2.5(1.9,3.0) | 5.0(4.2,5.7) | 2.5 |
| NCT Of Delhi | Central Delhi | 66.4(65.6,67.2) | 67.4(66.6,68.2) | 0.9 | 14.8(14.2,15.4) | 30.7(29.9,31.5) | 15.9 | 1.9(1.6,2.1) | 3.9(3.6,4.2) | 2.1 |
| NCT Of Delhi | West Delhi | 73.1(72.7,73.5) | 72.2(71.8,72.5) | -0.9 | 18.3(18.0,18.7) | 33.0(32.6,33.3) | 14.6 | 5.8(5.6,6.0) | 6.4(6.2,6.6) | 0.6 |
| NCT Of Delhi | South West Delhi | 75.5(75.1,75.8) | 75.1(74.7,75.5) | -0.4 | 19.2(18.8,19.5) | 36.5(36.1,36.9) | 17.4 | 5.3(5.1,5.5) | 6.5(6.2,6.7) | 1.2 |
| NCT Of Delhi | South Delhi | 64.4(64.0,64.8) | 74.2(73.9,74.6) | 9.8 | 23.4(23.1,23.8) | 39.1(38.7,39.5) | 15.7 | 2.2(2.1,2.3) | 6.6(6.4,6.9) | 4.5 |
| Rajasthan | Ganganagar | 65.6(65.2,66.1) | 83.4(83.0,83.8) | 17.8 | 31.7(31.3,32.2) | 45.8(45.4,46.3) | 14.1 | 2.3(2.1,2.4) | 6.7(6.4,6.9) | 4.4 |
| Rajasthan | Hanumangarh | 54.8(54.3,55.3) | 77.4(77.0,77.9) | 22.6 | 29.9(29.4,30.3) | 40.8(40.3,41.3) | 10.9 | 1.5(1.3,1.6) | 5.0(4.8,5.3) | 3.6 |
| Rajasthan | Bikaner | 62.9(62.4,63.3) | 81.2(80.8,81.5) | 18.3 | 24.5(24.1,24.9) | 33.4(33.0,33.8) | 8.9 | 0.9(0.8,1.0) | 3.5(3.3,3.7) | 2.6 |
| Rajasthan | Churu | 59.5(59.0,60.0) | 81.6(81.2,81.9) | 22.0 | 25.7(25.3,26.2) | 42.7(42.2,43.1) | 16.9 | 1.8(1.7,1.9) | 5.6(5.3,5.8) | 3.8 |
| Rajasthan | Jhunjhunu | 63.0(62.6,63.5) | 84.6(84.2,84.9) | 21.5 | 22.6(22.2,23.0) | 42.7(42.3,43.2) | 20.1 | 1.4(1.3,1.5) | 3.3(3.2,3.5) | 2.0 |
| Rajasthan | Alwar | 59.0(58.6,59.3) | 82.1(81.8,82.4) | 23.1 | 19.2(18.9,19.4) | 47.4(47.1,47.8) | 28.3 | 1.5(1.4,1.6) | 5.9(5.7,6.1) | 4.3 |
| Rajasthan | Bharatpur | 56.5(56.0,56.9) | 75.2(74.8,75.6) | 18.8 | 26.2(25.8,26.6) | 39.0(38.5,39.4) | 12.7 | 2.0(1.8,2.1) | 3.8(3.6,3.9) | 1.8 |
| Rajasthan | Dholpur | 54.7(54.0,55.3) | 77.7(77.1,78.2) | 23.0 | 24.9(24.3,25.5) | 47.5(46.9,48.2) | 22.6 | 1.7(1.5,1.8) | 3.3(3.0,3.5) | 1.6 |
| Rajasthan | Karauli | 53.9(53.4,54.5) | 79.4(78.9,79.9) | 25.4 | 19.3(18.8,19.8) | 23.6(23.1,24.1) | 4.3 | 1.3(1.2,1.5) | 1.9(1.7,2.1) | 0.6 |
| Rajasthan | Sawai Madhopur | 49.1(48.5,49.7) | 76.7(76.2,77.2) | 27.6 | 22.6(22.1,23.1) | 30.5(30.0,31.1) | 7.9 | 1.4(1.3,1.6) | 3.1(2.9,3.4) | 1.7 |
| Rajasthan | Dausa | 54.9(54.3,55.4) | 82.6(82.2,83.0) | 27.7 | 18.1(17.7,18.5) | 42.9(42.3,43.4) | 24.7 | 1.0(0.9,1.1) | 3.2(3.0,3.4) | 2.2 |
| Rajasthan | Jaipur | 72.0(71.8,72.3) | 80.2(80.0,80.4) | 8.2 | 37.1(36.8,37.3) | 46.5(46.2,46.8) | 9.4 | 1.5(1.5,1.6) | 4.1(4.0,4.2) | 2.6 |
| Rajasthan | Sikar | 61.1(60.7,61.5) | 79.1(78.8,79.4) | 18.0 | 23.6(23.3,24.0) | 38.6(38.2,39.0) | 14.9 | 0.7(0.7,0.8) | 3.0(2.9,3.2) | 2.3 |
| Rajasthan | Nagaur | 49.9(49.6,50.3) | 76.6(76.2,76.9) | 26.6 | 24.3(24.0,24.6) | 39.9(39.5,40.2) | 15.5 | 1.3(1.2,1.4) | 4.2(4.0,4.3) | 2.9 |
| Rajasthan | Jodhpur | 60.9(60.6,61.3) | 77.4(77.1,77.7) | 16.4 | 23.2(22.9,23.5) | 37.1(36.7,37.4) | 13.9 | 0.8(0.7,0.9) | 3.0(2.9,3.1) | 2.2 |
| Rajasthan | Jaisalmer | 45.7(44.8,46.6) | 75.4(74.6,76.2) | 29.7 | 17.1(16.5,17.8) | 37.7(36.8,38.6) | 20.6 | 0.9(0.7,1.1) | 2.6(2.3,2.9) | 1.7 |
| Rajasthan | Barmer | 48.4(48.0,48.9) | 80.1(79.7,80.4) | 31.6 | 17.9(17.5,18.2) | 34.2(33.8,34.7) | 16.3 | 0.7(0.6,0.7) | 2.8(2.6,2.9) | 2.1 |
| Rajasthan | Jalore | 53.6(53.1,54.1) | 76.6(76.1,77.0) | 23.0 | 20.4(20.0,20.9) | 42.4(41.9,42.9) | 22.0 | 2.0(1.9,2.1) | 5.2(5.0,5.5) | 3.2 |
| Rajasthan | Sirohi | 58.4(57.8,59.1) | 76.6(76.1,77.2) | 18.2 | 25.1(24.6,25.7) | 41.4(40.7,42.0) | 16.2 | 1.8(1.6,2.0) | 4.1(3.9,4.4) | 2.3 |
| Rajasthan | Pali | 58.4(58.0,58.9) | 83.2(82.8,83.5) | 24.8 | 24.9(24.5,25.3) | 45.7(45.2,46.2) | 20.9 | 2.1(2.0,2.3) | 5.9(5.7,6.1) | 3.8 |
| Rajasthan | Ajmer | 60.6(60.2,61.0) | 78.3(78.0,78.6) | 17.7 | 27.9(27.5,28.3) | 43.4(43.0,43.9) | 15.5 | 1.6(1.5,1.7) | 2.8(2.7,3.0) | 1.2 |
| Rajasthan | Tonk | 58.5(57.9,59.1) | 76.1(75.6,76.6) | 17.6 | 21.1(20.7,21.6) | 45.9(45.4,46.5) | 24.8 | 1.5(1.3,1.6) | 4.3(4.1,4.6) | 2.9 |
| Rajasthan | Bundi | 60.7(60.0,61.3) | 78.0(77.5,78.5) | 17.3 | 28.9(28.4,29.5) | 51.6(50.9,52.2) | 22.6 | 2.9(2.7,3.2) | 6.5(6.2,6.8) | 3.6 |
| Rajasthan | Bhilwara | 56.3(55.9,56.8) | 81.2(80.8,81.5) | 24.8 | 26.8(26.5,27.2) | 56.2(55.8,56.6) | 29.4 | 1.8(1.7,1.9) | 7.4(7.1,7.6) | 5.5 |
| Rajasthan | Rajsamand | 53.9(53.2,54.5) | 79.0(78.5,79.5) | 25.1 | 26.1(25.6,26.7) | 43.4(42.7,44.0) | 17.2 | 1.9(1.7,2.1) | 6.9(6.6,7.2) | 5.0 |
| Rajasthan | Dungarpur | 59.0(58.4,59.6) | 78.5(78.0,78.9) | 19.5 | 18.3(17.9,18.8) | 35.2(34.6,35.7) | 16.9 | 1.7(1.5,1.8) | 3.4(3.2,3.6) | 1.7 |
| Rajasthan | Banswara | 55.4(54.9,55.9) | 78.6(78.2,79.1) | 23.2 | 19.4(19.0,19.8) | 38.2(37.7,38.7) | 18.8 | 1.4(1.3,1.6) | 2.6(2.4,2.8) | 1.2 |
| Rajasthan | Chittorgarh | 57.1(56.6,57.6) | 82.5(82.1,82.9) | 25.4 | 28.1(27.6,28.6) | 50.6(50.1,51.1) | 22.5 | 1.6(1.5,1.8) | 7.0(6.8,7.3) | 5.4 |
| Rajasthan | Kota | 69.1(68.6,69.5) | 84.7(84.4,85.1) | 15.7 | 42.7(42.3,43.2) | 48.7(48.2,49.2) | 6.0 | 3.1(3.0,3.3) | 5.0(4.7,5.2) | 1.8 |
| Rajasthan | Baran | 58.6(58.0,59.3) | 78.6(78.0,79.1) | 19.9 | 31.3(30.7,31.9) | 40.4(39.8,41.0) | 9.1 | 1.9(1.7,2.0) | 4.3(4.0,4.5) | 2.4 |
| Rajasthan | Jhalawar | 57.4(56.9,58.0) | 76.1(75.6,76.6) | 18.7 | 31.1(30.6,31.6) | 43.6(43.0,44.1) | 12.5 | 1.7(1.6,1.9) | 4.2(3.9,4.4) | 2.4 |
| Rajasthan | Udaipur | 56.3(55.9,56.7) | 77.1(76.7,77.4) | 20.7 | 25.6(25.2,25.9) | 32.4(32.0,32.8) | 6.8 | 1.6(1.5,1.7) | 3.3(3.2,3.4) | 1.7 |
| Rajasthan | Pratapgarh | 63.3(62.5,64.0) | 76.0(75.4,76.6) | 12.7 | 21.0(20.4,21.6) | 38.5(37.8,39.2) | 17.4 | 1.5(1.3,1.6) | 3.3(3.0,3.6) | 1.8 |
| Uttar Pradesh | Saharanpur | 58.5(58.1,58.9) | 75.8(75.5,76.1) | 17.3 | 31.3(31.0,31.7) | 36.7(36.3,37.0) | 5.4 | 3.0(2.9,3.2) | 4.1(4.0,4.3) | 1.1 |
| Uttar Pradesh | Muzaffarnagar | 57.8(57.4,58.1) | 75.3(75.0,75.6) | 17.5 | 35.9(35.5,36.2) | 36.1(35.7,36.4) | 0.2 | 2.5(2.4,2.6) | 3.9(3.7,4.0) | 1.4 |
| Uttar Pradesh | Bijnor | 57.5(57.2,57.9) | 80.1(79.8,80.4) | 22.6 | 23.6(23.3,23.9) | 41.9(41.5,42.3) | 18.3 | 1.8(1.7,1.9) | 3.6(3.5,3.8) | 1.8 |
| Uttar Pradesh | Moradabad | 49.5(49.2,49.9) | 71.9(71.6,72.2) | 22.4 | 23.6(23.4,23.9) | 34.6(34.3,34.9) | 11.0 | 1.4(1.3,1.4) | 2.9(2.8,3.0) | 1.5 |
| Uttar Pradesh | Rampur | 46.3(45.8,46.8) | 70.2(69.8,70.7) | 23.9 | 24.2(23.8,24.6) | 34.6(34.1,35.0) | 10.3 | 1.8(1.7,1.9) | 4.9(4.7,5.1) | 3.1 |
| Uttar Pradesh | Amroha | 45.6(45.1,46.1) | 71.7(71.2,72.1) | 26.1 | 23.1(22.7,23.5) | 32.8(32.3,33.3) | 9.7 | 1.9(1.7,2.0) | 4.2(4.0,4.4) | 2.3 |
| Uttar Pradesh | Meerut | 60.3(59.9,60.7) | 77.2(76.9,77.5) | 16.9 | 32.0(31.7,32.3) | 38.4(38.1,38.8) | 6.4 | 3.5(3.4,3.6) | 3.2(3.0,3.3) | -0.3 |
| Uttar Pradesh | Baghpat | 58.6(58.0,59.2) | 76.8(76.3,77.3) | 18.2 | 37.2(36.6,37.8) | 46.2(45.6,46.8) | 9.0 | 1.8(1.6,2.0) | 4.8(4.5,5.0) | 3.0 |
| Uttar Pradesh | Ghaziabad | 64.9(64.6,65.2) | 79.1(78.8,79.4) | 14.2 | 32.8(32.5,33.1) | 36.2(35.9,36.5) | 3.4 | 4.2(4.1,4.3) | 3.9(3.8,4.0) | -0.3 |
| Uttar Pradesh | Gautam Buddha Nagar | 62.2(61.7,62.8) | 79.0(78.6,79.5) | 16.8 | 43.6(43.1,44.2) | 48.5(48.0,49.0) | 4.9 | 3.6(3.4,3.8) | 3.9(3.7,4.1) | 0.3 |
| Uttar Pradesh | Bulandshahr | 53.7(53.3,54.0) | 75.7(75.4,76.1) | 22.1 | 30.1(29.7,30.4) | 37.0(36.6,37.3) | 6.9 | 2.3(2.2,2.5) | 4.9(4.8,5.1) | 2.6 |
| Uttar Pradesh | Aligarh | 51.3(51.0,51.7) | 77.4(77.1,77.7) | 26.1 | 29.4(29.1,29.7) | 46.4(46.0,46.8) | 17.0 | 2.5(2.4,2.6) | 6.0(5.9,6.2) | 3.5 |
| Uttar Pradesh | Hathras | 49.8(49.3,50.4) | 74.6(74.1,75.1) | 24.8 | 32.9(32.3,33.4) | 38.3(37.7,38.8) | 5.4 | 2.5(2.3,2.7) | 5.7(5.5,6.0) | 3.2 |
| Uttar Pradesh | Mathura | 60.6(60.2,61.0) | 74.1(73.7,74.5) | 13.5 | 33.6(33.1,34.0) | 33.3(32.8,33.7) | -0.3 | 2.3(2.1,2.4) | 5.3(5.1,5.5) | 3.0 |
| Uttar Pradesh | Agra | 56.8(56.5,57.2) | 74.7(74.4,75.0) | 17.9 | 33.8(33.5,34.1) | 38.8(38.5,39.2) | 5.0 | 3.4(3.3,3.5) | 3.1(3.0,3.2) | -0.3 |
| Uttar Pradesh | Firozabad | 56.2(55.8,56.7) | 72.0(71.6,72.4) | 15.8 | 34.5(34.1,34.9) | 40.6(40.2,41.1) | 6.1 | 3.4(3.3,3.6) | 5.4(5.2,5.6) | 1.9 |
| Uttar Pradesh | Mainpuri | 50.9(50.4,51.4) | 72.5(72.1,73.0) | 21.6 | 24.3(23.8,24.7) | 42.1(41.5,42.6) | 17.8 | 1.8(1.7,1.9) | 5.6(5.4,5.9) | 3.8 |
| Uttar Pradesh | Budaun | 45.8(45.4,46.2) | 60.6(60.2,61.0) | 14.8 | 33.8(33.5,34.2) | 38.8(38.4,39.2) | 5.0 | 1.6(1.5,1.7) | 4.3(4.1,4.4) | 2.7 |
| Uttar Pradesh | Bareilly | 50.9(50.5,51.2) | 77.3(77.1,77.6) | 26.5 | 31.1(30.8,31.5) | 43.0(42.6,43.3) | 11.8 | 1.9(1.8,2.0) | 5.8(5.7,6.0) | 3.9 |
| Uttar Pradesh | Pilibhit | 56.8(56.3,57.3) | 73.8(73.4,74.2) | 17.0 | 38.3(37.8,38.8) | 46.1(45.6,46.6) | 7.8 | 2.8(2.6,3.0) | 4.8(4.6,5.0) | 2.0 |
| Uttar Pradesh | Shahjahanpur | 48.7(48.3,49.2) | 76.9(76.6,77.3) | 28.2 | 33.7(33.3,34.1) | 31.0(30.6,31.4) | -2.7 | 2.6(2.5,2.8) | 3.2(3.0,3.3) | 0.5 |
| Uttar Pradesh | Kheri | 54.4(54.0,54.7) | 74.5(74.1,74.8) | 20.1 | 26.6(26.3,26.9) | 36.9(36.5,37.2) | 10.3 | 3.3(3.1,3.4) | 3.9(3.7,4.0) | 0.6 |
| Uttar Pradesh | Sitapur | 42.1(41.8,42.5) | 74.2(73.9,74.5) | 32.1 | 19.1(18.8,19.3) | 39.6(39.3,39.9) | 20.5 | 1.7(1.6,1.8) | 3.6(3.4,3.7) | 1.9 |
| Uttar Pradesh | Hardoi | 52.0(51.6,52.3) | 71.7(71.3,72.0) | 19.7 | 23.3(23.0,23.6) | 38.9(38.5,39.2) | 15.5 | 2.3(2.2,2.4) | 4.2(4.1,4.4) | 1.9 |
| Uttar Pradesh | Unnao | 58.7(58.3,59.1) | 69.6(69.2,70.0) | 10.9 | 36.1(35.7,36.5) | 41.7(41.3,42.1) | 5.6 | 2.0(1.9,2.1) | 4.7(4.5,4.9) | 2.7 |
| Uttar Pradesh | Lucknow | 70.5(70.2,70.8) | 73.8(73.5,74.1) | 3.3 | 38.4(38.1,38.7) | 35.3(35.0,35.6) | -3.1 | 2.4(2.3,2.5) | 4.7(4.6,4.9) | 2.4 |
| Uttar Pradesh | Rae Bareli | 57.7(57.3,58.0) | 71.5(71.2,71.9) | 13.9 | 27.1(26.8,27.4) | 31.9(31.6,32.3) | 4.9 | 2.2(2.1,2.3) | 3.6(3.5,3.7) | 1.4 |
| Uttar Pradesh | Farrukhabad | 52.9(52.4,53.5) | 73.0(72.5,73.4) | 20.0 | 29.3(28.8,29.8) | 34.5(34.0,35.0) | 5.2 | 1.9(1.7,2.0) | 4.0(3.8,4.2) | 2.1 |
| Uttar Pradesh | Kannauj | 55.3(54.8,55.9) | 69.0(68.5,69.5) | 13.7 | 29.5(29.0,30.0) | 30.7(30.2,31.2) | 1.2 | 2.4(2.2,2.5) | 3.7(3.5,3.9) | 1.3 |
| Uttar Pradesh | Etawah | 48.1(47.6,48.7) | 76.0(75.5,76.5) | 27.9 | 27.3(26.8,27.8) | 42.9(42.3,43.4) | 15.6 | 2.2(2.1,2.4) | 4.9(4.6,5.1) | 2.7 |
| Uttar Pradesh | Auraiya | 64.7(64.1,65.3) | 76.6(76.0,77.1) | 11.8 | 29.6(29.0,30.1) | 39.8(39.2,40.4) | 10.3 | 3.9(3.7,4.1) | 5.9(5.7,6.2) | 2.0 |
| Uttar Pradesh | Kanpur Dehat | 60.0(59.5,60.5) | 82.5(82.1,82.9) | 22.5 | 39.1(38.6,39.6) | 38.6(38.1,39.1) | -0.5 | 3.7(3.5,3.9) | 4.5(4.3,4.7) | 0.8 |
| Uttar Pradesh | Kanpur Nagar | 65.8(65.5,66.1) | 73.2(72.9,73.5) | 7.4 | 34.2(33.9,34.5) | 30.6(30.3,30.9) | -3.6 | 3.2(3.1,3.3) | 4.3(4.2,4.4) | 1.1 |
| Uttar Pradesh | Jalaun | 53.2(52.6,53.7) | 75.0(74.6,75.5) | 21.9 | 28.7(28.2,29.2) | 31.6(31.2,32.1) | 2.9 | 2.6(2.4,2.8) | 3.4(3.2,3.6) | 0.8 |
| Uttar Pradesh | Jhansi | 64.2(63.8,64.7) | 75.7(75.3,76.1) | 11.5 | 30.7(30.3,31.2) | 34.1(33.7,34.6) | 3.4 | 2.6(2.5,2.8) | 4.5(4.3,4.7) | 1.9 |
| Uttar Pradesh | Lalitpur | 59.3(58.7,60.0) | 70.7(70.1,71.3) | 11.4 | 29.8(29.2,30.3) | 39.4(38.8,40.0) | 9.6 | 3.4(3.2,3.7) | 6.3(6.0,6.6) | 2.8 |
| Uttar Pradesh | Hamirpur | 62.1(61.5,62.7) | 73.5(72.9,74.1) | 11.4 | 27.1(26.5,27.7) | 36.5(35.8,37.1) | 9.4 | 3.5(3.3,3.8) | 5.0(4.7,5.3) | 1.5 |
| Uttar Pradesh | Mahoba | 51.7(51.0,52.5) | 74.7(74.0,75.3) | 23.0 | 22.9(22.3,23.5) | 33.5(32.8,34.2) | 10.6 | 1.8(1.6,2.0) | 5.0(4.6,5.3) | 3.2 |
| Uttar Pradesh | Banda | 53.6(53.1,54.1) | 77.4(76.9,77.8) | 23.8 | 23.0(22.5,23.4) | 37.5(37.0,38.0) | 14.5 | 2.0(1.9,2.2) | 5.4(5.1,5.6) | 3.4 |
| Uttar Pradesh | Chitrakoot | 54.6(53.9,55.3) | 75.0(74.4,75.6) | 20.4 | 27.9(27.2,28.5) | 27.9(27.2,28.6) | 0.0 | 1.9(1.7,2.1) | 3.4(3.1,3.7) | 1.5 |
| Uttar Pradesh | Fatehpur | 58.6(58.2,59.1) | 74.7(74.3,75.1) | 16.1 | 23.0(22.6,23.3) | 37.9(37.5,38.3) | 14.9 | 2.2(2.1,2.4) | 5.8(5.6,6.0) | 3.5 |
| Uttar Pradesh | Pratapgarh | 58.5(58.2,58.9) | 77.5(77.2,77.8) | 19.0 | 21.4(21.1,21.7) | 45.6(45.2,45.9) | 24.1 | 1.5(1.4,1.6) | 5.2(5.1,5.4) | 3.8 |
| Uttar Pradesh | Kaushambi | 59.6(59.0,60.1) | 76.4(75.9,76.9) | 16.8 | 25.3(24.8,25.8) | 32.9(32.4,33.5) | 7.7 | 2.7(2.6,2.9) | 3.4(3.2,3.6) | 0.7 |
| Uttar Pradesh | Prayagraj | 62.0(61.7,62.3) | 75.5(75.2,75.7) | 13.4 | 31.6(31.3,31.8) | 40.1(39.8,40.4) | 8.5 | 3.2(3.1,3.3) | 4.7(4.6,4.8) | 1.5 |
| Uttar Pradesh | Barabanki | 56.7(56.3,57.1) | 73.0(72.6,73.3) | 16.3 | 23.9(23.5,24.2) | 41.8(41.4,42.2) | 17.9 | 2.1(2.0,2.2) | 6.0(5.8,6.2) | 3.9 |
| Uttar Pradesh | Ayodhya | 61.2(60.8,61.6) | 82.5(82.1,82.8) | 21.3 | 23.0(22.7,23.4) | 37.8(37.4,38.2) | 14.8 | 1.6(1.5,1.7) | 3.2(3.1,3.4) | 1.6 |
| Uttar Pradesh | Ambedkar Nagar | 57.5(57.0,57.9) | 75.2(74.8,75.5) | 17.7 | 21.1(20.7,21.5) | 37.0(36.6,37.4) | 15.9 | 1.7(1.6,1.9) | 4.8(4.6,5.0) | 3.1 |
| Uttar Pradesh | Sultanpur | 52.6(52.3,53.0) | 78.4(78.1,78.7) | 25.8 | 20.2(20.0,20.5) | 35.9(35.6,36.3) | 15.7 | 1.5(1.4,1.6) | 5.1(5.0,5.3) | 3.6 |
| Uttar Pradesh | Bahraich | 38.3(37.9,38.7) | 70.5(70.1,70.8) | 32.2 | 22.0(21.7,22.3) | 33.5(33.2,33.9) | 11.5 | 2.0(1.9,2.1) | 4.7(4.5,4.8) | 2.7 |
| Uttar Pradesh | Shravasti | 38.8(38.1,39.5) | 67.0(66.4,67.7) | 28.2 | 21.0(20.4,21.6) | 38.2(37.5,38.8) | 17.2 | 1.7(1.5,1.9) | 3.2(2.9,3.4) | 1.5 |
| Uttar Pradesh | Balrampur | 43.1(42.6,43.6) | 72.3(71.9,72.8) | 29.2 | 22.6(22.2,23.0) | 37.1(36.6,37.6) | 14.5 | 1.8(1.7,1.9) | 4.5(4.3,4.7) | 2.7 |
| Uttar Pradesh | Gonda | 48.4(48.0,48.7) | 74.4(74.1,74.7) | 26.0 | 17.4(17.1,17.7) | 36.1(35.7,36.5) | 18.7 | 2.6(2.5,2.7) | 4.5(4.4,4.7) | 1.9 |
| Uttar Pradesh | Siddharth nagar | 45.3(44.9,45.7) | 73.6(73.2,73.9) | 28.3 | 16.8(16.5,17.2) | 30.0(29.6,30.4) | 13.1 | 1.2(1.1,1.3) | 4.2(4.0,4.4) | 3.0 |
| Uttar Pradesh | Basti | 55.9(55.5,56.3) | 81.5(81.2,81.9) | 25.6 | 20.2(19.8,20.5) | 33.6(33.2,34.0) | 13.4 | 1.3(1.2,1.4) | 2.8(2.7,3.0) | 1.5 |
| Uttar Pradesh | Sant Kabeer Nagar | 58.2(57.6,58.7) | 74.4(73.9,74.8) | 16.2 | 20.9(20.4,21.3) | 32.9(32.4,33.4) | 12.1 | 2.4(2.3,2.6) | 4.2(4.0,4.4) | 1.7 |
| Uttar Pradesh | Mahrajganj | 48.6(48.1,49.0) | 75.5(75.2,75.9) | 27.0 | 17.1(16.8,17.4) | 33.1(32.7,33.5) | 16.0 | 2.6(2.5,2.7) | 4.0(3.8,4.2) | 1.4 |
| Uttar Pradesh | Gorakhpur | 53.5(53.1,53.8) | 81.7(81.5,82.0) | 28.2 | 28.3(28.0,28.6) | 42.5(42.2,42.8) | 14.2 | 1.8(1.7,1.9) | 5.7(5.5,5.8) | 3.9 |
| Uttar Pradesh | Kushi Nagar | 48.5(48.1,48.9) | 77.1(76.8,77.4) | 28.6 | 16.5(16.2,16.8) | 32.4(32.1,32.7) | 15.9 | 2.7(2.6,2.8) | 3.9(3.7,4.0) | 1.2 |
| Uttar Pradesh | Deoria | 46.2(45.8,46.6) | 77.9(77.6,78.3) | 31.7 | 20.2(19.9,20.5) | 39.8(39.5,40.2) | 19.6 | 1.6(1.5,1.7) | 3.9(3.7,4.0) | 2.3 |
| Uttar Pradesh | Azamgarh | 57.5(57.2,57.9) | 80.5(80.3,80.8) | 23.0 | 23.6(23.4,23.9) | 45.1(44.8,45.4) | 21.5 | 2.1(2.0,2.1) | 3.5(3.4,3.6) | 1.4 |
| Uttar Pradesh | Mau | 53.6(53.1,54.1) | 80.7(80.3,81.0) | 27.1 | 22.2(21.8,22.6) | 32.2(31.8,32.6) | 10.0 | 1.1(1.1,1.2) | 2.8(2.7,3.0) | 1.7 |
| Uttar Pradesh | Ballia | 57.1(56.8,57.5) | 75.7(75.4,76.0) | 18.5 | 35.2(34.9,35.6) | 41.0(40.7,41.4) | 5.8 | 2.3(2.2,2.4) | 5.1(5.0,5.3) | 2.8 |
| Uttar Pradesh | Jaunpur | 55.3(55.0,55.6) | 78.6(78.3,78.9) | 23.3 | 24.8(24.5,25.0) | 32.5(32.2,32.8) | 7.7 | 1.8(1.7,1.9) | 3.3(3.2,3.5) | 1.6 |
| Uttar Pradesh | Ghazipur | 62.5(62.2,62.9) | 75.4(75.1,75.7) | 12.8 | 25.4(25.1,25.7) | 36.5(36.2,36.9) | 11.1 | 2.5(2.4,2.6) | 5.0(4.8,5.1) | 2.4 |
| Uttar Pradesh | Chandauli | 58.3(57.8,58.7) | 77.7(77.3,78.2) | 19.5 | 35.3(34.8,35.8) | 41.9(41.4,42.4) | 6.6 | 2.5(2.3,2.6) | 5.0(4.8,5.2) | 2.5 |
| Uttar Pradesh | Varanasi | 64.6(64.3,65.0) | 77.4(77.1,77.7) | 12.8 | 30.8(30.5,31.2) | 34.8(34.5,35.1) | 4.0 | 2.2(2.1,2.3) | 4.3(4.2,4.5) | 2.1 |
| Uttar Pradesh | Sant Ravidas Nagar (Bhadohi) | 55.9(55.3,56.4) | 75.4(74.9,75.8) | 19.5 | 27.2(26.7,27.6) | 44.9(44.3,45.4) | 17.7 | 2.6(2.4,2.7) | 5.2(5.0,5.5) | 2.7 |
| Uttar Pradesh | Mirzapur | 60.9(60.5,61.4) | 78.0(77.6,78.3) | 17.0 | 30.1(29.7,30.6) | 26.7(26.3,27.1) | -3.5 | 2.0(1.9,2.1) | 3.0(2.8,3.1) | 1.0 |
| Uttar Pradesh | Sonbhadra | 56.8(56.3,57.4) | 77.3(76.9,77.8) | 20.5 | 22.3(21.9,22.8) | 35.0(34.5,35.5) | 12.7 | 3.2(3.0,3.4) | 4.6(4.4,4.8) | 1.4 |
| Uttar Pradesh | Etah | 48.7(48.1,49.2) | 76.0(75.6,76.5) | 27.3 | 30.2(29.7,30.6) | 39.6(39.1,40.1) | 9.4 | 3.1(2.9,3.2) | 5.0(4.8,5.3) | 2.0 |
| Uttar Pradesh | Kasganj | 51.4(50.8,52.0) | 71.2(70.6,71.7) | 19.8 | 36.2(35.6,36.8) | 42.5(41.9,43.1) | 6.3 | 2.4(2.2,2.6) | 5.8(5.5,6.1) | 3.4 |
| Bihar | Pashchim Champaran | 25.0(24.7,25.3) | 77.3(77.0,77.6) | 52.3 | 22.5(22.2,22.9) | 63.3(62.9,63.7) | 40.8 | 4.9(4.7,5.0) | 13.6(13.3,13.8) | 8.7 |
| Bihar | Purba Champaran | 21.9(21.6,22.2) | 76.8(76.5,77.1) | 54.9 | 27.9(27.6,28.2) | 60.2(59.9,60.5) | 32.3 | 4.4(4.3,4.5) | 14.4(14.2,14.7) | 10.0 |
| Bihar | Sheohar | 26.6(25.8,27.4) | 78.7(78.0,79.5) | 52.1 | 30.3(29.4,31.1) | 66.2(65.3,67.1) | 35.9 | 5.1(4.7,5.5) | 15.3(14.6,15.9) | 10.2 |
| Bihar | Sitamarhi | 23.5(23.2,23.8) | 77.9(77.5,78.2) | 54.4 | 28.9(28.5,29.3) | 64.0(63.6,64.3) | 35.1 | 5.0(4.8,5.1) | 16.3(16.0,16.6) | 11.4 |
| Bihar | Madhubani | 24.6(24.3,24.9) | 77.6(77.3,77.9) | 53.0 | 26.6(26.3,26.9) | 65.7(65.4,66.0) | 39.1 | 4.4(4.3,4.6) | 12.0(11.8,12.2) | 7.5 |
| Bihar | Supaul | 27.0(26.5,27.4) | 76.6(76.1,77.0) | 49.6 | 31.2(30.8,31.7) | 60.1(59.6,60.5) | 28.8 | 6.9(6.7,7.2) | 11.6(11.3,11.9) | 4.7 |
| Bihar | Araria | 28.3(27.9,28.7) | 75.5(75.1,75.9) | 47.2 | 31.4(31.0,31.8) | 60.0(59.5,60.4) | 28.6 | 6.1(5.9,6.3) | 10.1(9.8,10.4) | 4.0 |
| Bihar | Kishanganj | 22.9(22.4,23.4) | 78.8(78.4,79.3) | 56.0 | 21.1(20.7,21.6) | 56.2(55.7,56.8) | 35.1 | 4.2(4.0,4.5) | 11.4(11.0,11.7) | 7.2 |
| Bihar | Purnia | 26.4(26.0,26.7) | 74.0(73.6,74.3) | 47.6 | 32.6(32.2,33.0) | 59.5(59.1,59.9) | 26.9 | 6.0(5.8,6.2) | 10.6(10.3,10.8) | 4.6 |
| Bihar | Katihar | 30.8(30.4,31.2) | 77.0(76.7,77.4) | 46.2 | 35.8(35.4,36.2) | 59.7(59.3,60.1) | 23.9 | 5.8(5.6,6.0) | 14.4(14.1,14.7) | 8.6 |
| Bihar | Madhepura | 25.1(24.6,25.5) | 75.5(75.0,75.9) | 50.4 | 34.5(34.0,35.0) | 62.9(62.4,63.4) | 28.4 | 6.1(5.8,6.3) | 12.1(11.7,12.4) | 6.0 |
| Bihar | Saharsa | 27.7(27.2,28.2) | 74.7(74.3,75.2) | 47.1 | 36.1(35.6,36.6) | 57.8(57.3,58.3) | 21.7 | 5.8(5.6,6.1) | 12.0(11.7,12.4) | 6.2 |
| Bihar | Darbhanga | 19.7(19.4,19.9) | 77.6(77.3,77.9) | 57.9 | 25.2(24.9,25.5) | 59.1(58.7,59.5) | 33.9 | 4.8(4.7,5.0) | 15.6(15.3,15.8) | 10.7 |
| Bihar | Muzaffarnagar | 22.8(22.5,23.0) | 78.9(78.7,79.2) | 56.2 | 26.6(26.3,26.9) | 66.6(66.3,66.9) | 40.0 | 4.3(4.2,4.5) | 16.3(16.0,16.5) | 12.0 |
| Bihar | Gopalganj | 28.8(28.4,29.2) | 82.3(82.0,82.6) | 53.5 | 19.1(18.8,19.5) | 61.4(60.9,61.8) | 42.2 | 5.8(5.6,6.0) | 15.9(15.6,16.2) | 10.1 |
| Bihar | Siwan | 31.8(31.4,32.2) | 77.5(77.1,77.8) | 45.6 | 24.3(24.0,24.6) | 59.1(58.8,59.5) | 34.9 | 4.8(4.6,4.9) | 11.3(11.1,11.6) | 6.6 |
| Bihar | Saran | 28.7(28.4,29.0) | 77.7(77.4,78.0) | 49.1 | 31.8(31.5,32.2) | 60.5(60.2,60.9) | 28.7 | 2.9(2.8,3.0) | 9.8(9.6,10.0) | 6.9 |
| Bihar | Vaishali | 32.4(32.1,32.8) | 78.6(78.3,78.9) | 46.2 | 32.0(31.6,32.3) | 62.7(62.3,63.1) | 30.7 | 3.6(3.5,3.8) | 16.5(16.3,16.8) | 12.9 |
| Bihar | Samastipur | 23.6(23.3,23.9) | 79.4(79.1,79.7) | 55.8 | 23.9(23.6,24.2) | 67.2(66.8,67.5) | 43.3 | 5.1(4.9,5.2) | 17.9(17.6,18.2) | 12.8 |
| Bihar | Begusarai | 29.7(29.3,30.1) | 76.8(76.4,77.1) | 47.1 | 31.7(31.3,32.1) | 59.5(59.1,59.9) | 27.8 | 4.4(4.2,4.5) | 16.6(16.3,17.0) | 12.3 |
| Bihar | Khagaria | 23.0(22.5,23.5) | 77.0(76.5,77.5) | 54.0 | 30.6(30.0,31.1) | 59.1(58.5,59.7) | 28.5 | 5.0(4.7,5.2) | 17.3(16.8,17.7) | 12.3 |
| Bihar | Bhagalpur | 30.9(30.5,31.3) | 77.2(76.9,77.6) | 46.4 | 27.8(27.4,28.2) | 59.6(59.2,60.1) | 31.8 | 5.7(5.5,5.8) | 13.6(13.4,13.9) | 8.0 |
| Bihar | Banka | 23.7(23.3,24.2) | 78.1(77.7,78.5) | 54.4 | 24.2(23.7,24.6) | 64.3(63.8,64.8) | 40.1 | 5.3(5.1,5.5) | 19.9(19.5,20.3) | 14.6 |
| Bihar | Munger | 32.7(32.2,33.3) | 72.0(71.4,72.5) | 39.2 | 40.4(39.8,41.0) | 56.2(55.5,56.8) | 15.8 | 5.8(5.5,6.0) | 14.4(13.9,14.8) | 8.6 |
| Bihar | Lakhisarai | 28.4(27.7,29.1) | 74.0(73.4,74.7) | 45.6 | 35.3(34.6,36.0) | 53.4(52.7,54.2) | 18.1 | 3.1(2.9,3.4) | 15.7(15.2,16.2) | 12.5 |
| Bihar | Sheikhpura | 28.2(27.3,29.0) | 77.9(77.1,78.6) | 49.7 | 28.2(27.4,29.0) | 62.6(61.7,63.5) | 34.4 | 3.5(3.2,3.9) | 18.2(17.5,18.9) | 14.7 |
| Bihar | Nalanda | 31.3(30.9,31.7) | 76.9(76.6,77.3) | 45.6 | 31.8(31.4,32.2) | 58.4(58.0,58.8) | 26.6 | 6.3(6.1,6.5) | 14.1(13.8,14.4) | 7.7 |
| Bihar | Patna | 39.4(39.1,39.7) | 77.5(77.2,77.7) | 38.1 | 40.7(40.4,41.0) | 60.3(60.0,60.6) | 19.7 | 4.1(4.0,4.2) | 13.1(12.9,13.2) | 9.0 |
| Bihar | Bhojpur | 29.6(29.2,30.0) | 71.3(70.9,71.7) | 41.7 | 38.8(38.4,39.3) | 63.7(63.3,64.1) | 24.9 | 3.5(3.4,3.7) | 14.2(13.9,14.5) | 10.7 |
| Bihar | Buxar | 31.4(30.9,31.9) | 71.6(71.1,72.1) | 40.3 | 39.5(38.9,40.0) | 64.6(64.1,65.1) | 25.2 | 3.2(3.0,3.4) | 13.4(13.0,13.7) | 10.2 |
| Bihar | Kaimur (Bhabua) | 34.2(33.6,34.7) | 78.0(77.5,78.5) | 43.8 | 33.5(32.9,34.0) | 61.5(61.0,62.1) | 28.0 | 4.8(4.5,5.0) | 11.4(11.1,11.8) | 6.7 |
| Bihar | Rohtas | 31.5(31.1,31.8) | 83.5(83.2,83.8) | 52.1 | 37.8(37.4,38.2) | 53.9(53.5,54.3) | 16.1 | 5.2(5.1,5.4) | 10.7(10.4,10.9) | 5.4 |
| Bihar | Aurangabad(BH) | 29.0(28.6,29.4) | 70.7(70.3,71.1) | 41.8 | 30.1(29.6,30.5) | 56.5(56.1,57.0) | 26.4 | 4.5(4.3,4.7) | 11.0(10.7,11.2) | 6.4 |
| Bihar | Gaya | 22.7(22.4,22.9) | 76.5(76.2,76.8) | 53.8 | 23.9(23.6,24.1) | 62.1(61.8,62.5) | 38.3 | 3.1(3.0,3.3) | 13.9(13.7,14.1) | 10.8 |
| Bihar | Nawada | 21.4(21.0,21.8) | 72.3(71.9,72.8) | 50.9 | 25.6(25.2,26.0) | 60.0(59.5,60.5) | 34.4 | 3.4(3.3,3.6) | 10.9(10.6,11.2) | 7.5 |
| Bihar | Jamui | 26.4(26.0,26.9) | 79.9(79.5,80.3) | 53.5 | 34.6(34.0,35.1) | 68.6(68.1,69.1) | 34.0 | 5.7(5.5,6.0) | 20.9(20.5,21.4) | 15.2 |
| Bihar | Jehanabad | 28.0(27.4,28.6) | 74.7(74.1,75.3) | 46.7 | 28.9(28.3,29.5) | 63.2(62.6,63.9) | 34.3 | 4.8(4.5,5.1) | 16.4(15.9,16.9) | 11.6 |
| Bihar | Arwal | 25.9(25.1,26.6) | 74.9(74.1,75.6) | 49.0 | 29.0(28.2,29.8) | 61.4(60.5,62.2) | 32.4 | 4.0(3.6,4.3) | 13.4(12.8,14.0) | 9.4 |
| Sikkim | North District | 63.8(60.6,67.1) | 81.0(78.3,83.7) | 17.2 | 35.6(32.4,38.9) | 41.4(38.0,44.7) | 5.7 | 4.0(2.6,5.3) | 10.6(8.5,12.7) | 6.7 |
| Sikkim | West District | 58.5(56.7,60.3) | 77.4(75.9,78.9) | 18.9 | 40.5(38.7,42.3) | 31.9(30.2,33.5) | -8.6 | 4.4(3.7,5.2) | 7.7(6.7,8.7) | 3.3 |
| Sikkim | South District | 60.5(58.8,62.2) | 77.0(75.6,78.5) | 16.5 | 49.2(47.4,50.9) | 40.8(39.1,42.5) | -8.4 | 4.7(4.0,5.4) | 13.4(12.2,14.6) | 8.7 |
| Sikkim | East District | 65.2(64.0,66.4) | 78.6(77.5,79.6) | 13.4 | 41.3(40.0,42.5) | 35.8(34.6,37.0) | -5.4 | 4.6(4.1,5.1) | 7.9(7.2,8.5) | 3.3 |
| Arunachal Pradesh | Tawang | 60.4(57.2,63.6) | 80.1(77.5,82.8) | 19.7 | 16.3(13.9,18.7) | 32.7(29.6,35.7) | 16.3 | 4.4(3.0,5.7) | 10.8(8.8,12.9) | 6.5 |
| Arunachal Pradesh | West Kameng | 65.9(63.6,68.2) | 80.8(78.8,82.7) | 14.9 | 20.0(18.1,22.0) | 32.2(29.9,34.5) | 12.2 | 3.7(2.8,4.6) | 11.6(10.0,13.1) | 7.9 |
| Arunachal Pradesh | East Kameng | 55.6(53.0,58.1) | 74.2(71.9,76.4) | 18.6 | 14.1(12.3,15.9) | 22.1(20.0,24.2) | 8.0 | 3.2(2.3,4.1) | 4.5(3.4,5.5) | 1.3 |
| Arunachal Pradesh | Papum Pare | 64.1(62.5,65.6) | 85.4(84.2,86.5) | 21.3 | 19.1(17.9,20.4) | 29.9(28.4,31.4) | 10.7 | 6.1(5.4,6.9) | 9.4(8.5,10.3) | 3.3 |
| Arunachal Pradesh | Upper Subansiri | 56.0(53.6,58.3) | 80.2(78.3,82.1) | 24.3 | 12.2(10.6,13.7) | 26.2(24.1,28.3) | 14.1 | 3.3(2.4,4.1) | 6.2(5.1,7.4) | 3.0 |
| Arunachal Pradesh | West Siang | 59.9(57.9,61.9) | 86.2(84.8,87.6) | 26.3 | 21.5(19.8,23.2) | 35.9(34.0,37.9) | 14.4 | 3.3(2.6,4.1) | 14.8(13.4,16.3) | 11.5 |
| Arunachal Pradesh | East Siang | 68.9(67.0,70.9) | 82.7(81.1,84.3) | 13.7 | 19.8(18.1,21.5) | 29.5(27.6,31.4) | 9.7 | 3.2(2.5,4.0) | 7.8(6.7,9.0) | 4.6 |
| Arunachal Pradesh | Upper Siang | 60.8(57.2,64.5) | 73.3(70.0,76.6) | 12.5 | 22.7(19.5,25.8) | 28.7(25.3,32.0) | 6.0 | 4.1(2.6,5.6) | 9.3(7.1,11.4) | 5.1 |
| Arunachal Pradesh | Changlang | 49.6(47.7,51.4) | 75.1(73.6,76.7) | 25.6 | 22.3(20.8,23.8) | 28.8(27.1,30.4) | 6.5 | 3.2(2.5,3.8) | 8.6(7.5,9.6) | 5.4 |
| Arunachal Pradesh | Tirap | 50.8(48.7,52.9) | 78.9(77.1,80.6) | 28.1 | 15.0(13.5,16.5) | 31.5(29.6,33.5) | 16.6 | 3.7(2.9,4.5) | 5.5(4.5,6.5) | 1.8 |
| Arunachal Pradesh | Lower Subansiri | 66.2(64.0,68.4) | 84.9(83.3,86.6) | 18.7 | 19.5(17.6,21.3) | 28.2(26.1,30.2) | 8.7 | 2.8(2.0,3.5) | 8.0(6.7,9.2) | 5.2 |
| Arunachal Pradesh | Kurung Kumey | 44.5(42.1,46.8) | 79.0(77.0,80.9) | 34.5 | 9.5(8.1,10.9) | 22.9(20.9,24.9) | 13.4 | 2.6(1.9,3.4) | 7.9(6.7,9.2) | 5.3 |
| Arunachal Pradesh | Dibang Valley | 57.2(49.3,65.1) | 80.4(74.0,86.7) | 23.2 | 22.2(15.6,28.9) | 30.0(22.7,37.3) | 7.8 | 4.6(1.3,8.0) | 11.8(6.6,16.9) | 7.2 |
| Arunachal Pradesh | Lower Dibang Valley | 53.6(50.6,56.6) | 79.7(77.3,82.1) | 26.0 | 23.1(20.6,25.7) | 27.8(25.1,30.5) | 4.7 | 4.6(3.4,5.9) | 12.9(10.9,14.9) | 8.2 |
| Arunachal Pradesh | Lohit | 58.8(56.9,60.6) | 71.4(69.7,73.1) | 12.7 | 26.4(24.8,28.0) | 28.3(26.7,30.0) | 1.9 | 3.8(3.1,4.5) | 8.5(7.5,9.5) | 4.7 |
| Arunachal Pradesh | Anjaw | 50.2(45.1,55.2) | 76.8(72.6,81.1) | 26.6 | 14.0(10.5,17.5) | 23.9(19.6,28.2) | 9.9 | 4.7(2.6,6.8) | 6.8(4.3,9.3) | 2.1 |
| Nagaland | Mon | 29.4(28.2,30.7) | 56.5(55.1,57.9) | 27.0 | 19.1(18.0,20.2) | 23.8(22.6,25.0) | 4.7 | 3.7(3.2,4.2) | 4.0(3.4,4.6) | 0.3 |
| Nagaland | Mokokchung | 49.1(47.6,50.6) | 59.4(58.0,60.9) | 10.3 | 29.6(28.2,30.9) | 28.4(27.0,29.7) | -1.2 | 6.9(6.2,7.7) | 4.1(3.5,4.7) | -2.8 |
| Nagaland | Zunheboto | 40.4(38.6,42.1) | 70.2(68.5,71.8) | 29.8 | 17.3(15.9,18.7) | 23.1(21.6,24.6) | 5.8 | 2.9(2.3,3.5) | 2.8(2.2,3.4) | -0.1 |
| Nagaland | Wokha | 46.8(45.2,48.4) | 73.1(71.6,74.5) | 26.3 | 17.5(16.2,18.7) | 22.7(21.3,24.1) | 5.2 | 3.1(2.5,3.7) | 2.0(1.6,2.5) | -1.1 |
| Nagaland | Dimapur | 41.9(40.8,43.0) | 62.8(61.7,63.8) | 20.9 | 27.0(26.0,28.0) | 27.2(26.2,28.1) | 0.1 | 4.9(4.5,5.4) | 2.3(2.0,2.6) | -2.6 |
| Nagaland | Phek | 33.6(31.9,35.2) | 65.4(63.8,67.1) | 31.8 | 12.9(11.8,14.1) | 20.2(18.8,21.6) | 7.3 | 3.4(2.8,4.0) | 3.0(2.5,3.6) | -0.4 |
| Nagaland | Tuensang | 32.8(31.2,34.3) | 56.7(55.1,58.3) | 24.0 | 23.8(22.4,25.1) | 22.2(20.8,23.5) | -1.6 | 3.6(3.0,4.2) | 3.6(3.0,4.2) | 0.0 |
| Nagaland | Longleng | 25.8(23.0,28.6) | 57.4(54.3,60.6) | 31.7 | 14.5(12.3,16.8) | 18.2(15.7,20.6) | 3.6 | 4.9(3.5,6.3) | 3.9(2.7,5.1) | -1.0 |
| Nagaland | Kiphire | 21.1(19.0,23.3) | 56.1(53.4,58.7) | 35.0 | 14.3(12.4,16.2) | 16.6(14.7,18.6) | 2.3 | 3.8(2.7,4.8) | 2.6(1.8,3.4) | -1.2 |
| Nagaland | Kohima | 49.7(48.4,51.1) | 73.2(72.0,74.3) | 23.4 | 21.7(20.6,22.8) | 22.8(21.7,23.9) | 1.1 | 4.0(3.4,4.5) | 2.8(2.4,3.3) | -1.1 |
| Nagaland | Peren | 32.4(30.3,34.6) | 60.9(58.7,63.1) | 28.5 | 18.2(16.5,20.0) | 24.4(22.5,26.4) | 6.2 | 4.8(3.9,5.8) | 4.8(3.9,5.8) | 0.0 |
| Manipur | Senapati | 29.9(29.0,30.8) | 70.2(69.3,71.1) | 40.3 | 32.6(31.7,33.5) | 33.4(32.5,34.3) | 0.8 | 4.4(4.0,4.8) | 5.6(5.2,6.1) | 1.2 |
| Manipur | Tamenglong | 32.6(30.9,34.3) | 69.1(67.4,70.7) | 36.5 | 31.4(29.7,33.1) | 32.5(30.9,34.2) | 1.1 | 2.4(1.8,2.9) | 4.4(3.7,5.2) | 2.1 |
| Manipur | Churachandpur | 34.3(33.1,35.4) | 79.3(78.3,80.3) | 45.1 | 30.8(29.6,31.9) | 37.6(36.4,38.9) | 6.9 | 2.6(2.2,3.0) | 4.6(4.1,5.1) | 2.0 |
| Manipur | Bishnupur | 34.7(33.4,36.0) | 71.7(70.5,72.9) | 37.0 | 47.5(46.2,48.9) | 48.0(46.7,49.4) | 0.5 | 4.7(4.2,5.3) | 9.2(8.4,9.9) | 4.4 |
| Manipur | Thoubal | 28.4(27.5,29.3) | 70.9(70.0,71.9) | 42.5 | 37.1(36.1,38.1) | 51.4(50.4,52.4) | 14.3 | 3.3(2.9,3.6) | 6.0(5.5,6.4) | 2.7 |
| Manipur | Imphal West | 39.8(39.0,40.7) | 77.3(76.5,78.0) | 37.5 | 51.6(50.7,52.5) | 46.4(45.5,47.2) | -5.3 | 6.0(5.6,6.4) | 6.4(6.0,6.8) | 0.4 |
| Manipur | Imphal East | 39.1(38.2,40.0) | 70.8(70.0,71.7) | 31.7 | 42.8(41.9,43.7) | 42.6(41.6,43.5) | -0.2 | 4.2(3.8,4.6) | 7.5(7.0,8.0) | 3.3 |
| Manipur | Ukhrul | 32.8(31.4,34.3) | 72.6(71.2,74.0) | 39.8 | 30.5(29.1,32.0) | 38.0(36.5,39.5) | 7.5 | 4.5(3.9,5.2) | 6.9(6.1,7.7) | 2.4 |
| Manipur | Chandel | 31.8(30.1,33.4) | 76.4(74.9,77.9) | 44.6 | 45.2(43.5,47.0) | 37.7(36.0,39.4) | -7.5 | 5.5(4.7,6.3) | 6.0(5.2,6.8) | 0.5 |
| Mizoram | Mamit | 39.7(37.3,42.0) | 78.6(76.7,80.6) | 39.0 | 8.1(6.8,9.4) | 15.1(13.3,16.8) | 7.0 | 1.3(0.7,1.8) | 3.4(2.6,4.3) | 2.1 |
| Mizoram | Kolasib | 52.9(50.5,55.2) | 81.4(79.6,83.3) | 28.6 | 9.9(8.5,11.3) | 13.1(11.5,14.7) | 3.2 | 1.2(0.7,1.8) | 2.3(1.6,3.1) | 1.1 |
| Mizoram | Aizawl | 67.7(66.7,68.6) | 86.8(86.1,87.5) | 19.1 | 11.1(10.5,11.7) | 17.2(16.4,17.9) | 6.1 | 2.3(2.0,2.6) | 2.5(2.1,2.8) | 0.1 |
| Mizoram | Champhai | 55.9(54.0,57.8) | 83.3(81.9,84.7) | 27.4 | 13.6(12.3,14.9) | 15.2(13.9,16.6) | 1.6 | 2.0(1.5,2.6) | 3.4(2.7,4.1) | 1.3 |
| Mizoram | Serchhip | 57.5(54.9,60.1) | 85.4(83.5,87.2) | 27.9 | 8.3(6.8,9.7) | 20.2(18.1,22.3) | 12.0 | 2.0(1.2,2.7) | 4.8(3.7,6.0) | 2.9 |
| Mizoram | Lunglei | 57.6(55.9,59.3) | 81.8(80.5,83.2) | 24.2 | 11.4(10.3,12.5) | 17.1(15.8,18.4) | 5.7 | 1.9(1.4,2.3) | 3.0(2.4,3.5) | 1.1 |
| Mizoram | Lawngtlai | 39.1(37.1,41.1) | 75.3(73.5,77.1) | 36.2 | 9.3(8.1,10.5) | 11.8(10.5,13.1) | 2.5 | 0.9(0.5,1.2) | 1.3(0.8,1.8) | 0.4 |
| Mizoram | Saiha | 50.4(47.6,53.3) | 82.2(80.0,84.4) | 31.7 | 13.6(11.6,15.6) | 18.1(15.9,20.3) | 4.5 | 1.6(0.9,2.3) | 2.2(1.3,3.0) | 0.6 |
| Tripura | West Tripura | 56.2(55.7,56.6) | 77.9(77.5,78.3) | 21.7 | 81.8(81.4,82.2) | 54.5(54.0,55.0) | -27.3 | 15.9(15.5,16.2) | 21.6(21.2,22.0) | 5.8 |
| Tripura | South Tripura | 57.3(56.6,58.0) | 73.8(73.2,74.4) | 16.5 | 82.1(81.5,82.6) | 63.1(62.4,63.8) | -19.0 | 11.4(11.0,11.9) | 20.5(19.9,21.1) | 9.1 |
| Tripura | Dhalai | 56.1(55.0,57.2) | 79.4(78.6,80.3) | 23.4 | 71.6(70.6,72.6) | 54.8(53.7,55.9) | -16.8 | 10.8(10.1,11.5) | 17.7(16.9,18.6) | 6.9 |
| Tripura | North Tripura | 52.3(51.5,53.1) | 73.8(73.1,74.5) | 21.5 | 78.5(77.9,79.2) | 59.1(58.3,59.9) | -19.4 | 12.7(12.1,13.2) | 20.5(19.9,21.1) | 7.8 |
| Meghalaya | West Garo Hills | 51.7(50.9,52.6) | 73.5(72.7,74.3) | 21.8 | 30.2(29.4,31.0) | 18.8(18.1,19.4) | -11.5 | 5.0(4.6,5.4) | 4.9(4.5,5.3) | -0.1 |
| Meghalaya | East Garo Hills | 53.6(52.4,54.9) | 60.8(59.6,62.1) | 7.2 | 24.2(23.1,25.3) | 16.6(15.7,17.6) | -7.6 | 4.1(3.6,4.6) | 3.9(3.4,4.4) | -0.2 |
| Meghalaya | South Garo Hills | 50.3(48.4,52.2) | 71.7(70.0,73.4) | 21.4 | 25.1(23.5,26.8) | 23.3(21.7,25.0) | -1.8 | 3.6(2.9,4.3) | 6.2(5.3,7.2) | 2.7 |
| Meghalaya | West Khasi Hills | 55.0(53.8,56.2) | 72.9(71.8,73.9) | 17.9 | 17.5(16.5,18.4) | 19.7(18.8,20.7) | 2.3 | 2.6(2.2,2.9) | 7.5(6.9,8.1) | 4.9 |
| Meghalaya | Ribhoi | 55.6(54.2,57.1) | 68.0(66.6,69.3) | 12.3 | 19.9(18.8,21.1) | 24.1(22.9,25.4) | 4.2 | 2.5(2.1,3.0) | 8.4(7.6,9.2) | 5.9 |
| Meghalaya | East Khasi Hills | 63.2(62.5,63.9) | 74.3(73.7,75.0) | 11.1 | 20.9(20.3,21.6) | 19.0(18.4,19.6) | -1.9 | 2.8(2.6,3.1) | 5.1(4.8,5.5) | 2.3 |
| Meghalaya | Jaintia Hills | 53.1(51.9,54.2) | 68.6(67.5,69.6) | 15.5 | 16.7(15.9,17.6) | 24.1(23.1,25.1) | 7.3 | 3.7(3.3,4.2) | 9.1(8.5,9.8) | 5.4 |
| Assam | Kokrajhar | 36.3(35.6,37.0) | 72.2(71.5,72.8) | 35.9 | 32.8(32.1,33.5) | 59.4(58.7,60.1) | 26.6 | 4.5(4.2,4.8) | 10.5(10.1,11.0) | 6.0 |
| Assam | Dhubri | 33.0(32.5,33.4) | 71.9(71.5,72.4) | 38.9 | 31.4(30.9,31.9) | 60.3(59.8,60.8) | 28.9 | 6.7(6.4,6.9) | 9.0(8.7,9.3) | 2.4 |
| Assam | Goalpara | 34.5(33.9,35.2) | 77.5(76.9,78.1) | 43.0 | 26.5(25.9,27.1) | 65.0(64.4,65.7) | 38.5 | 3.8(3.5,4.1) | 12.0(11.5,12.4) | 8.2 |
| Assam | Barpeta | 30.7(30.2,31.2) | 76.5(76.0,76.9) | 45.8 | 27.2(26.7,27.7) | 59.1(58.6,59.6) | 31.9 | 4.9(4.7,5.1) | 11.7(11.4,12.0) | 6.8 |
| Assam | Marigaon | 46.0(45.3,46.7) | 73.9(73.3,74.5) | 27.9 | 42.5(41.8,43.2) | 67.5(66.9,68.2) | 25.0 | 7.6(7.2,8.0) | 11.2(10.7,11.6) | 3.6 |
| Assam | Nagaon | 43.7(43.3,44.1) | 78.3(78.0,78.6) | 34.6 | 35.3(34.9,35.7) | 56.8(56.4,57.2) | 21.4 | 5.0(4.9,5.2) | 10.2(10.0,10.5) | 5.2 |
| Assam | Sonitpur | 49.4(48.9,49.9) | 78.4(78.0,78.8) | 29.0 | 39.5(39.0,40.0) | 64.4(63.9,64.9) | 24.9 | 8.4(8.1,8.7) | 14.3(13.9,14.6) | 5.9 |
| Assam | Lakhimpur | 50.5(49.9,51.2) | 80.0(79.5,80.5) | 29.4 | 37.9(37.3,38.6) | 57.6(56.9,58.2) | 19.7 | 4.9(4.6,5.2) | 14.4(14.0,14.9) | 9.5 |
| Assam | Dhemaji | 42.7(41.9,43.5) | 79.9(79.2,80.6) | 37.2 | 33.8(33.0,34.6) | 59.0(58.2,59.8) | 25.2 | 5.4(5.0,5.8) | 13.4(12.8,13.9) | 8.0 |
| Assam | Tinsukia | 46.4(45.8,46.9) | 74.5(74.0,75.0) | 28.1 | 38.7(38.2,39.3) | 70.4(69.9,71.0) | 31.7 | 5.1(4.8,5.3) | 17.4(17.0,17.8) | 12.3 |
| Assam | Dibrugarh | 45.6(45.0,46.1) | 80.4(80.0,80.9) | 34.9 | 41.7(41.2,42.3) | 73.2(72.7,73.7) | 31.5 | 5.8(5.5,6.0) | 16.5(16.1,16.9) | 10.7 |
| Assam | Sivasagar | 51.7(51.1,52.3) | 81.8(81.4,82.3) | 30.1 | 47.3(46.7,47.9) | 70.2(69.6,70.7) | 22.9 | 8.7(8.4,9.1) | 18.6(18.2,19.1) | 9.9 |
| Assam | Jorhat | 49.6(48.9,50.2) | 81.0(80.5,81.5) | 31.4 | 44.2(43.6,44.8) | 61.5(60.9,62.1) | 17.3 | 9.3(8.9,9.6) | 20.3(19.8,20.8) | 11.0 |
| Assam | Golaghat | 53.8(53.2,54.5) | 85.1(84.6,85.5) | 31.2 | 53.8(53.1,54.4) | 67.4(66.8,68.0) | 13.7 | 10.9(10.5,11.3) | 18.1(17.6,18.6) | 7.2 |
| Assam | Karbi Anglong | 42.7(42.0,43.4) | 79.8(79.2,80.4) | 37.1 | 33.7(33.1,34.4) | 66.8(66.1,67.5) | 33.1 | 4.1(3.8,4.4) | 11.8(11.4,12.3) | 7.7 |
| Assam | Dima Hasao | 51.7(50.2,53.2) | 85.3(84.3,86.4) | 33.6 | 34.9(33.5,36.3) | 62.7(61.3,64.2) | 27.8 | 4.0(3.4,4.6) | 8.9(8.1,9.8) | 4.9 |
| Assam | Cachar | 45.6(45.1,46.1) | 74.0(73.5,74.4) | 28.4 | 36.4(35.9,36.9) | 57.2(56.7,57.7) | 20.9 | 6.6(6.4,6.9) | 10.3(10.0,10.6) | 3.7 |
| Assam | Karimganj | 36.4(35.8,37.0) | 77.1(76.5,77.6) | 40.7 | 25.2(24.6,25.7) | 60.9(60.2,61.5) | 35.7 | 3.1(2.9,3.4) | 13.3(12.9,13.7) | 10.1 |
| Assam | Hailakandi | 36.2(35.4,37.0) | 77.8(77.0,78.5) | 41.6 | 24.5(23.8,25.2) | 62.0(61.1,62.8) | 37.5 | 3.6(3.3,3.9) | 12.6(12.1,13.2) | 9.1 |
| Assam | Bongaigaon | 41.3(40.5,42.1) | 76.7(76.0,77.3) | 35.4 | 36.0(35.3,36.8) | 61.2(60.5,62.0) | 25.2 | 4.8(4.4,5.1) | 10.3(9.8,10.7) | 5.5 |
| Assam | Chirang | 38.7(37.7,39.6) | 73.2(72.4,74.1) | 34.5 | 32.6(31.7,33.5) | 59.3(58.3,60.2) | 26.6 | 4.4(4.0,4.8) | 10.5(9.9,11.1) | 6.1 |
| Assam | Kamrup | 51.5(50.9,52.0) | 85.7(85.3,86.1) | 34.2 | 42.1(41.6,42.6) | 59.4(58.8,59.9) | 17.3 | 7.2(6.9,7.5) | 10.4(10.1,10.7) | 3.2 |
| Assam | Kamrup Metro | 69.8(69.2,70.3) | 81.9(81.5,82.3) | 12.1 | 55.1(54.6,55.7) | 72.5(72.0,73.0) | 17.4 | 10.0(9.7,10.4) | 14.5(14.1,14.9) | 4.5 |
| Assam | Nalbari | 47.9(47.1,48.6) | 82.3(81.7,82.9) | 34.4 | 30.8(30.1,31.5) | 67.0(66.3,67.7) | 36.2 | 4.6(4.3,4.9) | 14.5(14.0,15.1) | 10.0 |
| Assam | Baksa | 46.0(45.3,46.6) | 76.5(75.9,77.1) | 30.6 | 34.9(34.3,35.6) | 64.2(63.5,64.8) | 29.3 | 4.5(4.2,4.8) | 11.0(10.5,11.4) | 6.5 |
| Assam | Darrang | 42.2(41.4,42.9) | 74.5(73.8,75.1) | 32.3 | 42.0(41.3,42.7) | 69.2(68.6,69.9) | 27.2 | 8.7(8.3,9.1) | 14.2(13.7,14.7) | 5.6 |
| Assam | Udalguri | 49.8(49.0,50.5) | 79.5(78.9,80.1) | 29.8 | 37.9(37.2,38.6) | 65.0(64.3,65.7) | 27.1 | 4.7(4.4,5.0) | 17.8(17.3,18.4) | 13.1 |
| West Bengal | Darjeeling | 56.5(56.0,57.0) | 80.4(80.0,80.7) | 23.9 | 52.0(51.6,52.5) | 50.4(49.9,50.9) | -1.6 | 7.3(7.0,7.5) | 11.9(11.6,12.2) | 4.7 |
| West Bengal | Jalpaiguri | 43.3(42.9,43.6) | 77.7(77.4,77.9) | 34.4 | 34.0(33.7,34.3) | 47.6(47.2,47.9) | 13.6 | 10.0(9.8,10.2) | 11.1(10.9,11.3) | 1.1 |
| West Bengal | Coochbehar | 34.5(34.2,34.9) | 78.2(77.8,78.5) | 43.6 | 35.0(34.7,35.4) | 47.9(47.5,48.3) | 12.9 | 8.1(7.8,8.3) | 14.0(13.7,14.3) | 6.0 |
| West Bengal | Dinajpur Uttar | 34.6(34.2,34.9) | 78.6(78.3,79.0) | 44.1 | 41.5(41.1,41.9) | 55.7(55.3,56.1) | 14.2 | 8.4(8.2,8.7) | 15.7(15.4,16.0) | 7.3 |
| West Bengal | Dinajpur Dakshin | 43.7(43.2,44.2) | 73.5(73.1,74.0) | 29.8 | 53.0(52.5,53.5) | 57.2(56.7,57.7) | 4.2 | 12.9(12.5,13.2) | 14.9(14.5,15.2) | 2.0 |
| West Bengal | Maldah | 37.4(37.1,37.7) | 69.7(69.4,70.1) | 32.3 | 47.7(47.3,48.0) | 51.7(51.3,52.0) | 4.0 | 10.6(10.3,10.8) | 12.8(12.6,13.1) | 2.3 |
| West Bengal | Murshidabad | 32.9(32.7,33.1) | 73.4(73.1,73.6) | 40.5 | 37.7(37.5,38.0) | 53.8(53.5,54.0) | 16.0 | 7.1(6.9,7.2) | 12.4(12.3,12.6) | 5.4 |
| West Bengal | Birbhum | 40.1(39.8,40.5) | 76.4(76.1,76.7) | 36.2 | 50.4(50.0,50.7) | 57.9(57.6,58.3) | 7.6 | 7.4(7.2,7.5) | 18.3(18.0,18.5) | 10.9 |
| West Bengal | Bardhaman | 41.5(41.3,41.7) | 77.3(77.1,77.5) | 35.8 | 45.8(45.6,46.0) | 49.5(49.3,49.8) | 3.7 | 7.6(7.5,7.7) | 11.1(10.9,11.2) | 3.5 |
| West Bengal | Nadia | 45.1(44.8,45.4) | 72.0(71.8,72.3) | 26.9 | 45.6(45.3,45.8) | 52.3(52.0,52.6) | 6.7 | 9.8(9.6,10.0) | 13.7(13.5,13.9) | 3.9 |
| West Bengal | 24 Parganas North | 56.0(55.8,56.2) | 78.5(78.3,78.7) | 22.5 | 53.0(52.8,53.2) | 52.6(52.4,52.8) | -0.5 | 8.3(8.2,8.4) | 13.4(13.3,13.6) | 5.2 |
| West Bengal | Hooghly | 50.8(50.5,51.1) | 80.3(80.1,80.5) | 29.5 | 51.6(51.3,51.9) | 58.5(58.3,58.8) | 6.9 | 14.8(14.6,15.0) | 19.5(19.3,19.8) | 4.7 |
| West Bengal | Bankura | 39.2(38.8,39.5) | 76.7(76.5,77.0) | 37.6 | 40.9(40.5,41.2) | 43.1(42.7,43.4) | 2.2 | 6.0(5.9,6.2) | 10.7(10.5,10.9) | 4.6 |
| West Bengal | Purulia | 34.7(34.4,35.1) | 68.6(68.2,69.0) | 33.9 | 43.6(43.2,44.0) | 48.2(47.8,48.6) | 4.6 | 9.0(8.8,9.3) | 8.8(8.6,9.1) | -0.2 |
| West Bengal | Howrah | 52.2(51.9,52.5) | 81.4(81.2,81.6) | 29.2 | 50.1(49.9,50.4) | 58.9(58.6,59.2) | 8.8 | 12.1(11.9,12.2) | 15.5(15.3,15.7) | 3.4 |
| West Bengal | Kolkata | 53.3(53.0,53.5) | 83.5(83.3,83.8) | 30.3 | 45.1(44.8,45.4) | 55.2(54.9,55.5) | 10.1 | 3.6(3.5,3.8) | 7.3(7.2,7.5) | 3.7 |
| West Bengal | 24 Parganas South | 43.6(43.4,43.8) | 79.8(79.6,80.0) | 36.2 | 44.7(44.5,44.9) | 64.8(64.5,65.0) | 20.1 | 10.4(10.2,10.5) | 13.9(13.7,14.0) | 3.5 |
| West Bengal | Medinipur West | 45.7(45.4,45.9) | 78.6(78.4,78.9) | 33.0 | 45.9(45.6,46.1) | 46.4(46.2,46.7) | 0.6 | 7.1(7.0,7.3) | 12.9(12.7,13.1) | 5.8 |
| West Bengal | Medinipur East | 43.0(42.7,43.3) | 71.6(71.4,71.9) | 28.6 | 48.4(48.1,48.7) | 51.8(51.5,52.1) | 3.4 | 10.0(9.9,10.2) | 16.4(16.1,16.6) | 6.3 |
| Jharkhand | Garhwa | 36.8(36.2,37.4) | 80.1(79.6,80.6) | 43.3 | 44.2(43.6,44.9) | 66.8(66.2,67.3) | 22.5 | 5.8(5.5,6.1) | 16.4(15.9,16.9) | 10.6 |
| Jharkhand | Chatra | 36.5(35.8,37.1) | 80.7(80.1,81.2) | 44.2 | 27.8(27.2,28.5) | 62.0(61.3,62.7) | 34.1 | 4.6(4.3,4.9) | 16.8(16.3,17.3) | 12.2 |
| Jharkhand | Koderma | 35.7(34.8,36.5) | 81.9(81.3,82.6) | 46.3 | 33.3(32.5,34.1) | 57.2(56.4,58.1) | 23.9 | 2.6(2.4,2.9) | 12.3(11.8,12.9) | 9.7 |
| Jharkhand | Giridih | 36.5(36.1,37.0) | 76.4(76.0,76.8) | 39.8 | 35.2(34.8,35.7) | 52.0(51.6,52.5) | 16.8 | 3.3(3.1,3.5) | 11.1(10.8,11.4) | 7.8 |
| Jharkhand | Deoghar | 35.8(35.2,36.3) | 80.3(79.8,80.8) | 44.5 | 40.7(40.1,41.3) | 64.5(64.0,65.1) | 23.8 | 5.7(5.5,6.0) | 18.8(18.3,19.2) | 13.0 |
| Jharkhand | Godda | 41.5(40.9,42.1) | 78.4(77.9,78.9) | 36.9 | 39.6(39.0,40.2) | 55.6(54.9,56.2) | 16.0 | 6.1(5.8,6.4) | 13.8(13.4,14.2) | 7.7 |
| Jharkhand | Sahebganj | 40.4(39.7,41.0) | 77.8(77.2,78.3) | 37.4 | 38.6(37.9,39.2) | 60.1(59.4,60.7) | 21.5 | 5.6(5.3,5.9) | 10.4(10.0,10.8) | 4.9 |
| Jharkhand | Pakur | 37.6(36.9,38.3) | 78.7(78.1,79.3) | 41.1 | 43.6(42.9,44.3) | 67.4(66.7,68.1) | 23.8 | 5.1(4.8,5.4) | 18.0(17.4,18.6) | 12.9 |
| Jharkhand | Dhanbad | 45.0(44.6,45.4) | 76.7(76.3,77.0) | 31.7 | 42.4(42.0,42.8) | 54.6(54.2,55.0) | 12.1 | 5.8(5.6,6.0) | 8.1(7.8,8.3) | 2.3 |
| Jharkhand | Bokaro | 51.0(50.5,51.4) | 80.5(80.1,80.9) | 29.5 | 44.9(44.4,45.4) | 56.6(56.1,57.1) | 11.7 | 4.5(4.3,4.7) | 10.9(10.6,11.1) | 6.4 |
| Jharkhand | Lohardaga | 49.7(48.7,50.7) | 86.6(85.9,87.3) | 36.9 | 38.2(37.2,39.2) | 66.5(65.5,67.5) | 28.3 | 6.5(6.0,7.0) | 18.8(18.0,19.6) | 12.3 |
| Jharkhand | East Singhbhum | 51.0(50.6,51.5) | 82.2(81.8,82.5) | 31.1 | 43.4(42.9,43.8) | 56.2(55.7,56.6) | 12.8 | 4.9(4.7,5.0) | 14.1(13.8,14.4) | 9.2 |
| Jharkhand | Palamu | 37.4(36.9,37.9) | 81.6(81.2,82.0) | 44.1 | 28.5(28.0,29.0) | 62.2(61.7,62.7) | 33.7 | 5.6(5.3,5.8) | 14.7(14.3,15.1) | 9.2 |
| Jharkhand | Latehar | 38.9(38.0,39.7) | 80.3(79.6,81.0) | 41.4 | 43.5(42.7,44.4) | 66.3(65.5,67.1) | 22.7 | 4.7(4.3,5.0) | 15.2(14.6,15.8) | 10.6 |
| Jharkhand | Hazaribagh | 44.0(43.5,44.6) | 83.0(82.6,83.4) | 38.9 | 41.7(41.2,42.2) | 61.6(61.1,62.2) | 20.0 | 6.6(6.3,6.9) | 17.2(16.8,17.6) | 10.6 |
| Jharkhand | Ramgarh | 54.2(53.5,54.9) | 81.5(80.9,82.0) | 27.3 | 43.6(42.9,44.3) | 57.8(57.1,58.5) | 14.2 | 5.3(5.0,5.6) | 12.8(12.3,13.2) | 7.5 |
| Jharkhand | Dumka | 38.7(38.1,39.3) | 79.7(79.2,80.2) | 41.0 | 37.3(36.8,37.9) | 61.9(61.4,62.5) | 24.6 | 6.9(6.6,7.2) | 13.3(12.9,13.7) | 6.4 |
| Jharkhand | Jamtara | 38.6(37.9,39.4) | 75.4(74.7,76.0) | 36.7 | 36.4(35.7,37.2) | 58.3(57.5,59.0) | 21.9 | 3.9(3.6,4.2) | 11.3(10.8,11.8) | 7.3 |
| Jharkhand | Ranchi | 55.2(54.8,55.6) | 81.8(81.5,82.1) | 26.6 | 45.7(45.3,46.1) | 62.5(62.1,62.9) | 16.8 | 9.7(9.5,10.0) | 16.7(16.4,17.0) | 7.0 |
| Jharkhand | Khunti | 44.5(43.5,45.4) | 82.0(81.2,82.7) | 37.5 | 43.5(42.5,44.4) | 66.5(65.6,67.4) | 23.1 | 5.1(4.7,5.5) | 15.6(14.9,16.3) | 10.5 |
| Jharkhand | Gumla | 47.6(46.9,48.3) | 82.9(82.3,83.4) | 35.3 | 36.5(35.8,37.1) | 59.8(59.1,60.4) | 23.3 | 5.9(5.6,6.3) | 10.6(10.2,11.0) | 4.7 |
| Jharkhand | Simdega | 45.5(44.7,46.4) | 83.8(83.2,84.5) | 38.3 | 42.0(41.1,42.9) | 65.2(64.3,66.0) | 23.2 | 6.1(5.7,6.5) | 17.3(16.6,18.0) | 11.2 |
| Jharkhand | West Singhbhum | 47.6(47.0,48.2) | 77.3(76.9,77.8) | 29.7 | 39.1(38.5,39.6) | 60.1(59.5,60.6) | 21.0 | 6.6(6.3,6.8) | 9.3(9.0,9.6) | 2.7 |
| Jharkhand | Saraikela Kharsawan | 48.1(47.5,48.8) | 78.7(78.1,79.2) | 30.5 | 35.0(34.4,35.6) | 62.5(61.8,63.1) | 27.5 | 5.1(4.8,5.4) | 15.0(14.6,15.5) | 9.9 |
| Odisha | Bargarh | 59.3(58.8,59.9) | 86.8(86.4,87.1) | 27.4 | 70.2(69.8,70.7) | 69.3(68.8,69.8) | -0.9 | 13.5(13.2,13.9) | 23.8(23.3,24.2) | 10.2 |
| Odisha | Jharsuguda | 53.4(52.6,54.3) | 86.8(86.3,87.4) | 33.4 | 68.2(67.4,69.0) | 78.2(77.5,78.9) | 10.1 | 14.4(13.8,15.0) | 26.5(25.7,27.2) | 12.0 |
| Odisha | Sambalpur | 61.4(60.8,62.1) | 86.9(86.5,87.4) | 25.5 | 69.0(68.5,69.6) | 70.8(70.3,71.4) | 1.8 | 13.9(13.4,14.3) | 23.4(22.9,24.0) | 9.6 |
| Odisha | Deogarh | 58.8(57.6,59.9) | 90.0(89.3,90.7) | 31.3 | 68.9(67.8,70.0) | 79.1(78.2,80.1) | 10.2 | 17.7(16.8,18.6) | 32.3(31.2,33.3) | 14.5 |
| Odisha | Sundargarh | 58.2(57.7,58.6) | 86.7(86.4,87.0) | 28.5 | 70.8(70.4,71.2) | 74.1(73.7,74.5) | 3.3 | 15.9(15.5,16.2) | 23.7(23.3,24.1) | 7.9 |
| Odisha | Kendujhar | 53.7(53.3,54.2) | 81.1(80.7,81.5) | 27.4 | 64.9(64.4,65.3) | 70.7(70.3,71.2) | 5.9 | 13.9(13.6,14.3) | 20.2(19.8,20.6) | 6.3 |
| Odisha | Mayurbhanj | 57.8(57.4,58.2) | 85.9(85.6,86.2) | 28.1 | 67.7(67.3,68.1) | 70.8(70.4,71.2) | 3.1 | 14.2(13.9,14.5) | 21.5(21.1,21.8) | 7.3 |
| Odisha | Baleshwar | 58.2(57.8,58.6) | 87.1(86.8,87.4) | 28.9 | 62.4(62.0,62.8) | 75.2(74.8,75.6) | 12.8 | 16.0(15.6,16.3) | 24.8(24.5,25.2) | 8.9 |
| Odisha | Bhadrak | 54.4(53.9,54.9) | 88.9(88.6,89.2) | 34.5 | 71.2(70.7,71.7) | 80.3(79.9,80.8) | 9.1 | 15.3(14.9,15.7) | 29.7(29.2,30.1) | 14.4 |
| Odisha | Kendrapara | 58.8(58.3,59.3) | 91.8(91.5,92.1) | 33.0 | 72.3(71.9,72.8) | 71.4(70.9,71.9) | -1.0 | 18.0(17.6,18.4) | 21.2(20.8,21.6) | 3.1 |
| Odisha | Jagatsinghapur | 58.7(58.2,59.3) | 89.8(89.4,90.1) | 31.0 | 68.3(67.7,68.8) | 74.2(73.6,74.7) | 5.9 | 18.2(17.7,18.6) | 23.5(23.0,24.0) | 5.3 |
| Odisha | Cuttack | 58.8(58.4,59.2) | 84.6(84.3,84.9) | 25.8 | 68.4(68.1,68.8) | 70.7(70.4,71.1) | 2.3 | 16.8(16.5,17.1) | 29.3(29.0,29.7) | 12.5 |
| Odisha | Jajapur | 57.5(57.0,57.9) | 90.7(90.4,91.0) | 33.2 | 76.2(75.8,76.6) | 76.1(75.7,76.5) | -0.2 | 19.5(19.1,19.8) | 22.3(21.9,22.7) | 2.8 |
| Odisha | Dhenkanal | 55.6(55.0,56.2) | 89.0(88.6,89.3) | 33.4 | 65.5(64.9,66.0) | 79.4(78.9,79.9) | 14.0 | 13.4(13.0,13.8) | 35.4(34.9,36.0) | 22.0 |
| Odisha | Anugul | 56.5(55.9,57.0) | 88.5(88.1,88.8) | 32.0 | 68.1(67.5,68.6) | 82.6(82.2,83.1) | 14.6 | 17.5(17.1,18.0) | 24.8(24.3,25.3) | 7.3 |
| Odisha | Nayagarh | 53.7(53.0,54.3) | 88.4(87.9,88.8) | 34.7 | 66.5(65.8,67.1) | 79.5(78.9,80.0) | 13.0 | 12.8(12.4,13.3) | 36.3(35.7,37.0) | 23.5 |
| Odisha | Khordha | 64.0(63.6,64.5) | 88.0(87.7,88.2) | 23.9 | 74.7(74.3,75.0) | 74.7(74.3,75.1) | 0.0 | 12.3(12.0,12.6) | 26.4(26.1,26.8) | 14.2 |
| Odisha | Puri | 64.1(63.6,64.6) | 88.0(87.6,88.3) | 23.9 | 79.1(78.7,79.5) | 81.7(81.3,82.1) | 2.5 | 19.3(18.9,19.7) | 32.3(31.9,32.8) | 13.0 |
| Odisha | Ganjam | 51.3(50.9,51.6) | 85.9(85.7,86.2) | 34.7 | 62.6(62.3,62.9) | 73.7(73.4,74.0) | 11.1 | 8.4(8.2,8.6) | 26.8(26.5,27.1) | 18.4 |
| Odisha | Gajapati | 49.2(48.3,50.1) | 86.3(85.7,86.9) | 37.1 | 62.5(61.7,63.4) | 73.7(72.9,74.5) | 11.2 | 12.3(11.7,12.9) | 28.7(27.9,29.5) | 16.4 |
| Odisha | Kandhamal | 59.1(58.3,59.8) | 87.2(86.6,87.7) | 28.1 | 65.8(65.1,66.5) | 70.3(69.6,71.0) | 4.5 | 15.3(14.8,15.9) | 19.4(18.8,20.0) | 4.1 |
| Odisha | Boudh | 54.2(53.2,55.2) | 85.5(84.8,86.2) | 31.3 | 67.2(66.3,68.2) | 72.7(71.8,73.6) | 5.5 | 17.4(16.6,18.2) | 19.7(18.9,20.5) | 2.3 |
| Odisha | Sonepur | 52.2(51.4,53.0) | 86.3(85.7,86.9) | 34.1 | 65.2(64.4,66.0) | 76.9(76.2,77.6) | 11.7 | 13.8(13.2,14.3) | 22.0(21.3,22.7) | 8.3 |
| Odisha | Balangir | 52.2(51.7,52.7) | 84.9(84.5,85.3) | 32.7 | 72.6(72.2,73.1) | 77.2(76.8,77.6) | 4.6 | 13.3(13.0,13.7) | 27.7(27.3,28.2) | 14.4 |
| Odisha | Nuapada | 55.2(54.4,56.0) | 85.5(84.9,86.1) | 30.3 | 67.6(66.9,68.4) | 70.3(69.6,71.1) | 2.7 | 16.5(15.9,17.1) | 22.2(21.5,22.9) | 5.7 |
| Odisha | Kalahandi | 47.7(47.2,48.2) | 85.6(85.2,86.0) | 37.9 | 59.0(58.5,59.5) | 71.7(71.2,72.2) | 12.7 | 11.8(11.5,12.1) | 19.7(19.3,20.1) | 7.9 |
| Odisha | Rayagada | 51.4(50.8,52.1) | 86.1(85.7,86.6) | 34.7 | 56.6(55.9,57.2) | 73.5(73.0,74.1) | 17.0 | 9.6(9.2,10.0) | 27.8(27.2,28.4) | 18.2 |
| Odisha | Nabarangpur | 49.9(49.3,50.5) | 83.3(82.9,83.8) | 33.4 | 65.2(64.6,65.8) | 75.2(74.7,75.7) | 10.0 | 12.6(12.2,13.1) | 19.0(18.5,19.5) | 6.4 |
| Odisha | Koraput | 53.1(52.5,53.6) | 83.1(82.6,83.5) | 30.0 | 63.5(62.9,64.0) | 66.8(66.3,67.3) | 3.3 | 9.1(8.8,9.5) | 23.7(23.2,24.2) | 14.6 |
| Odisha | Malkangiri | 52.2(51.3,53.1) | 82.8(82.2,83.5) | 30.6 | 64.2(63.4,65.1) | 71.8(71.0,72.6) | 7.6 | 12.8(12.2,13.4) | 17.7(17.0,18.4) | 4.9 |
| Chhattisgarh | Korea | 51.9(51.0,52.7) | 75.1(74.4,75.8) | 23.3 | 51.7(50.9,52.5) | 40.8(40.0,41.6) | -10.9 | 5.0(4.6,5.3) | 5.5(5.1,5.8) | 0.5 |
| Chhattisgarh | Surguja | 43.9(43.5,44.4) | 81.4(81.1,81.8) | 37.5 | 38.9(38.4,39.3) | 50.7(50.2,51.1) | 11.8 | 4.9(4.7,5.1) | 7.8(7.6,8.1) | 2.9 |
| Chhattisgarh | Jashpur | 50.0(49.3,50.7) | 81.3(80.7,81.8) | 31.3 | 48.3(47.6,49.0) | 48.5(47.8,49.2) | 0.2 | 6.0(5.7,6.3) | 5.2(4.9,5.5) | -0.8 |
| Chhattisgarh | Raigarh | 51.7(51.1,52.2) | 80.3(79.9,80.8) | 28.7 | 47.1(46.6,47.6) | 50.1(49.6,50.7) | 3.0 | 5.8(5.5,6.0) | 7.1(6.8,7.4) | 1.3 |
| Chhattisgarh | Korba | 53.9(53.3,54.5) | 83.3(82.8,83.7) | 29.4 | 50.5(49.9,51.1) | 35.0(34.4,35.6) | -15.5 | 5.4(5.2,5.7) | 4.3(4.1,4.6) | -1.1 |
| Chhattisgarh | Janjgir - Champa | 54.9(54.4,55.4) | 81.3(80.9,81.7) | 26.4 | 53.9(53.4,54.4) | 41.7(41.2,42.2) | -12.2 | 4.0(3.8,4.2) | 7.8(7.5,8.0) | 3.8 |
| Chhattisgarh | Bilaspur | 56.3(55.9,56.7) | 81.4(81.1,81.7) | 25.1 | 63.5(63.1,63.9) | 56.6(56.2,57.0) | -6.9 | 6.0(5.8,6.2) | 8.4(8.2,8.6) | 2.4 |
| Chhattisgarh | Kabirdham | 36.4(35.7,37.1) | 78.8(78.2,79.4) | 42.4 | 41.0(40.2,41.7) | 50.5(49.7,51.3) | 9.5 | 3.3(3.0,3.6) | 6.7(6.3,7.1) | 3.4 |
| Chhattisgarh | Rajnandgaon | 55.3(54.7,55.8) | 86.4(86.1,86.8) | 31.2 | 58.2(57.7,58.7) | 40.0(39.5,40.6) | -18.1 | 5.5(5.3,5.8) | 6.0(5.7,6.2) | 0.4 |
| Chhattisgarh | Durg | 57.9(57.6,58.3) | 85.2(85.0,85.5) | 27.3 | 52.9(52.6,53.3) | 56.6(56.2,56.9) | 3.7 | 4.2(4.0,4.3) | 8.0(7.8,8.2) | 3.8 |
| Chhattisgarh | Raipur | 53.3(53.0,53.6) | 82.5(82.2,82.7) | 29.2 | 55.7(55.3,56.0) | 46.9(46.6,47.3) | -8.7 | 4.4(4.2,4.5) | 5.0(4.9,5.1) | 0.6 |
| Chhattisgarh | Mahasamund | 48.7(48.1,49.3) | 81.0(80.5,81.5) | 32.3 | 45.9(45.2,46.5) | 49.9(49.2,50.5) | 4.0 | 3.0(2.8,3.2) | 9.2(8.8,9.6) | 6.2 |
| Chhattisgarh | Dhamtari | 56.4(55.7,57.1) | 81.3(80.7,81.9) | 24.9 | 50.2(49.4,50.9) | 54.4(53.7,55.1) | 4.2 | 3.7(3.5,4.0) | 9.9(9.5,10.4) | 6.2 |
| Chhattisgarh | Uttar Bastar Kanker | 54.6(53.9,55.4) | 82.6(82.1,83.2) | 28.0 | 56.3(55.6,57.1) | 50.9(50.2,51.7) | -5.4 | 4.5(4.2,4.8) | 9.1(8.7,9.5) | 4.6 |
| Chhattisgarh | Bastar | 46.5(45.9,47.0) | 73.4(72.9,73.9) | 27.0 | 44.4(43.9,45.0) | 41.6(41.0,42.1) | -2.9 | 4.8(4.6,5.1) | 6.3(6.1,6.6) | 1.5 |
| Chhattisgarh | Narayanpur | 44.9(43.1,46.8) | 78.7(77.2,80.2) | 33.8 | 56.3(54.4,58.1) | 54.1(52.3,56.0) | -2.1 | 3.8(3.1,4.5) | 7.7(6.7,8.7) | 3.9 |
| Chhattisgarh | Dakshin Bastar Dantewada | 49.4(48.5,50.4) | 77.6(76.9,78.4) | 28.2 | 40.9(40.0,41.8) | 40.1(39.2,41.0) | -0.8 | 3.0(2.6,3.3) | 6.0(5.6,6.4) | 3.1 |
| Chhattisgarh | Bijapur | 41.1(39.8,42.5) | 77.5(76.4,78.7) | 36.4 | 52.8(51.4,54.2) | 52.9(51.5,54.3) | 0.1 | 3.8(3.3,4.3) | 7.5(6.8,8.3) | 3.7 |
| Madhya Pradesh | Sheopur | 29.5(28.7,30.3) | 71.2(70.5,72.0) | 41.7 | 30.0(29.2,30.8) | 40.3(39.5,41.2) | 10.3 | 2.9(2.6,3.2) | 6.8(6.4,7.2) | 3.9 |
| Madhya Pradesh | Morena | 27.1(26.6,27.5) | 76.8(76.4,77.3) | 49.8 | 23.3(22.9,23.8) | 41.9(41.4,42.4) | 18.6 | 2.7(2.5,2.8) | 4.1(3.9,4.3) | 1.5 |
| Madhya Pradesh | Bhind | 28.0(27.5,28.5) | 74.2(73.8,74.7) | 46.2 | 27.3(26.8,27.8) | 33.3(32.8,33.8) | 6.0 | 3.5(3.3,3.7) | 4.2(4.0,4.4) | 0.7 |
| Madhya Pradesh | Gwalior | 36.5(36.0,37.0) | 77.5(77.1,77.9) | 41.0 | 30.4(30.0,30.9) | 48.0(47.6,48.5) | 17.6 | 4.5(4.3,4.7) | 7.0(6.8,7.3) | 2.5 |
| Madhya Pradesh | Datia | 30.5(29.8,31.3) | 75.2(74.5,75.8) | 44.6 | 31.6(30.9,32.4) | 44.4(43.6,45.2) | 12.8 | 4.5(4.2,4.9) | 6.0(5.6,6.3) | 1.4 |
| Madhya Pradesh | Shivpuri | 32.4(31.9,32.9) | 67.8(67.3,68.4) | 35.5 | 27.3(26.8,27.7) | 44.2(43.7,44.8) | 17.0 | 4.1(3.9,4.3) | 5.2(5.0,5.5) | 1.1 |
| Madhya Pradesh | Tikamgarh | 32.4(31.9,33.0) | 73.0(72.5,73.5) | 40.6 | 23.9(23.4,24.4) | 46.7(46.1,47.3) | 22.8 | 4.5(4.3,4.8) | 6.8(6.5,7.1) | 2.3 |
| Madhya Pradesh | Chhatarpur | 32.1(31.6,32.7) | 72.2(71.7,72.7) | 40.0 | 34.7(34.2,35.2) | 45.4(44.9,46.0) | 10.8 | 3.5(3.3,3.7) | 6.6(6.4,6.9) | 3.2 |
| Madhya Pradesh | Panna | 31.8(31.1,32.4) | 72.5(71.9,73.1) | 40.7 | 29.1(28.4,29.7) | 47.3(46.6,48.0) | 18.3 | 3.3(3.1,3.6) | 6.6(6.2,6.9) | 3.2 |
| Madhya Pradesh | Sagar | 39.3(38.9,39.7) | 79.5(79.1,79.8) | 40.2 | 34.2(33.8,34.6) | 61.2(60.7,61.6) | 27.0 | 5.7(5.5,5.9) | 7.6(7.3,7.8) | 1.9 |
| Madhya Pradesh | Damoh | 33.4(32.8,34.0) | 74.7(74.2,75.3) | 41.3 | 27.6(27.1,28.2) | 50.7(50.1,51.3) | 23.1 | 2.8(2.6,3.0) | 11.0(10.6,11.4) | 8.2 |
| Madhya Pradesh | Satna | 34.8(34.4,35.3) | 82.2(81.8,82.5) | 47.3 | 35.9(35.4,36.3) | 40.6(40.1,41.0) | 4.7 | 5.5(5.3,5.7) | 6.2(6.0,6.4) | 0.7 |
| Madhya Pradesh | Rewa | 38.4(38.0,38.8) | 77.7(77.3,78.0) | 39.3 | 31.8(31.4,32.3) | 42.8(42.4,43.3) | 11.0 | 3.6(3.4,3.7) | 8.5(8.3,8.8) | 5.0 |
| Madhya Pradesh | Umaria | 34.4(33.6,35.2) | 69.3(68.5,70.1) | 34.8 | 28.7(27.9,29.5) | 44.1(43.3,45.0) | 15.4 | 4.4(4.1,4.8) | 6.2(5.7,6.6) | 1.7 |
| Madhya Pradesh | Neemuch | 33.7(33.0,34.3) | 75.2(74.6,75.8) | 41.6 | 29.5(28.9,30.2) | 51.9(51.1,52.6) | 22.3 | 3.2(2.9,3.4) | 7.8(7.4,8.2) | 4.6 |
| Madhya Pradesh | Mandsaur | 30.6(30.1,31.2) | 73.7(73.2,74.2) | 43.1 | 29.7(29.1,30.2) | 50.7(50.1,51.2) | 21.0 | 4.0(3.8,4.3) | 11.1(10.7,11.4) | 7.0 |
| Madhya Pradesh | Ratlam | 30.5(30.0,31.0) | 72.3(71.8,72.8) | 41.8 | 28.2(27.7,28.7) | 46.9(46.4,47.5) | 18.7 | 3.6(3.4,3.8) | 9.3(9.0,9.6) | 5.7 |
| Madhya Pradesh | Ujjain | 35.5(35.1,36.0) | 75.2(74.8,75.7) | 39.7 | 36.7(36.3,37.2) | 54.1(53.6,54.6) | 17.4 | 3.6(3.4,3.8) | 8.8(8.5,9.1) | 5.2 |
| Madhya Pradesh | Shajapur | 33.4(32.8,33.9) | 74.2(73.7,74.7) | 40.9 | 31.0(30.5,31.5) | 46.5(45.9,47.0) | 15.5 | 3.4(3.2,3.6) | 7.7(7.5,8.0) | 4.4 |
| Madhya Pradesh | Dewas | 38.3(37.8,38.8) | 66.9(66.4,67.4) | 28.6 | 40.2(39.7,40.8) | 49.8(49.3,50.4) | 9.6 | 4.3(4.1,4.5) | 8.5(8.2,8.8) | 4.2 |
| Madhya Pradesh | Dhar | 31.3(30.9,31.7) | 75.0(74.6,75.4) | 43.7 | 27.5(27.1,27.9) | 39.5(39.0,39.9) | 12.0 | 3.9(3.7,4.1) | 6.1(5.9,6.3) | 2.2 |
| Madhya Pradesh | Indore | 53.0(52.6,53.3) | 76.5(76.1,76.8) | 23.5 | 39.7(39.3,40.0) | 47.8(47.4,48.1) | 8.1 | 3.7(3.5,3.8) | 7.6(7.4,7.8) | 3.9 |
| Madhya Pradesh | West Nimar | 39.4(38.9,39.9) | 78.5(78.1,78.9) | 39.1 | 29.0(28.5,29.4) | 33.0(32.6,33.5) | 4.1 | 3.9(3.7,4.1) | 5.5(5.3,5.7) | 1.5 |
| Madhya Pradesh | Barwani | 34.8(34.2,35.4) | 72.3(71.7,72.8) | 37.5 | 22.0(21.5,22.5) | 42.0(41.4,42.6) | 20.0 | 3.2(3.0,3.4) | 6.6(6.3,6.9) | 3.4 |
| Madhya Pradesh | Rajgarh | 31.1(30.6,31.6) | 75.1(74.7,75.6) | 44.0 | 23.9(23.4,24.4) | 48.8(48.2,49.3) | 24.9 | 5.9(5.7,6.2) | 9.9(9.6,10.2) | 4.0 |
| Madhya Pradesh | Vidisha | 31.6(31.1,32.2) | 75.4(74.9,75.9) | 43.8 | 28.2(27.7,28.8) | 47.2(46.6,47.8) | 19.0 | 3.1(2.9,3.3) | 8.9(8.6,9.3) | 5.9 |
| Madhya Pradesh | Bhopal | 53.3(52.9,53.8) | 77.7(77.3,78.0) | 24.3 | 45.7(45.2,46.1) | 53.1(52.7,53.5) | 7.4 | 5.4(5.2,5.6) | 8.5(8.2,8.7) | 3.0 |
| Madhya Pradesh | Sehore | 30.6(30.1,31.2) | 73.7(73.2,74.3) | 43.1 | 30.9(30.3,31.4) | 47.9(47.3,48.5) | 17.0 | 3.7(3.5,3.9) | 10.2(9.9,10.6) | 6.5 |
| Madhya Pradesh | Raisen | 40.7(40.1,41.3) | 76.5(76.0,77.0) | 35.8 | 45.3(44.7,45.9) | 56.4(55.8,57.0) | 11.1 | 5.4(5.2,5.7) | 10.5(10.2,10.9) | 5.1 |
| Madhya Pradesh | Betul | 48.9(48.3,49.4) | 76.8(76.4,77.3) | 28.0 | 30.7(30.2,31.2) | 47.4(46.9,47.9) | 16.7 | 5.9(5.6,6.1) | 10.4(10.1,10.7) | 4.6 |
| Madhya Pradesh | Harda | 38.8(37.9,39.6) | 69.7(68.8,70.5) | 30.9 | 40.9(40.0,41.7) | 50.0(49.1,50.9) | 9.1 | 4.5(4.1,4.9) | 8.7(8.2,9.3) | 4.2 |
| Madhya Pradesh | Hoshangabad | 37.0(36.4,37.5) | 72.4(71.9,73.0) | 35.5 | 31.2(30.6,31.7) | 40.6(40.0,41.2) | 9.5 | 4.8(4.6,5.1) | 8.5(8.2,8.9) | 3.7 |
| Madhya Pradesh | Katni | 38.0(37.4,38.6) | 71.4(70.9,72.0) | 33.4 | 40.0(39.4,40.6) | 52.5(51.9,53.1) | 12.6 | 3.9(3.6,4.1) | 8.5(8.2,8.9) | 4.7 |
| Madhya Pradesh | Jabalpur | 46.8(46.4,47.2) | 79.3(79.0,79.7) | 32.5 | 38.3(37.9,38.8) | 47.7(47.3,48.1) | 9.3 | 3.7(3.6,3.9) | 8.2(7.9,8.4) | 4.5 |
| Madhya Pradesh | Narsimhapur | 41.7(41.1,42.3) | 73.7(73.1,74.3) | 32.0 | 32.8(32.2,33.4) | 48.6(48.0,49.2) | 15.8 | 5.5(5.2,5.8) | 7.4(7.1,7.8) | 1.9 |
| Madhya Pradesh | Dindori | 37.4(36.6,38.2) | 77.6(77.0,78.3) | 40.2 | 25.7(25.0,26.4) | 45.4(44.6,46.2) | 19.7 | 5.4(5.1,5.8) | 5.5(5.2,5.9) | 0.1 |
| Madhya Pradesh | Mandla | 42.3(41.7,42.9) | 78.9(78.4,79.4) | 36.6 | 24.4(23.9,25.0) | 43.7(43.1,44.4) | 19.3 | 3.3(3.1,3.5) | 7.4(7.1,7.7) | 4.1 |
| Madhya Pradesh | Chhindwara | 39.6(39.2,40.0) | 83.2(82.8,83.5) | 43.6 | 28.2(27.8,28.6) | 48.4(48.0,48.9) | 20.2 | 2.7(2.6,2.9) | 6.7(6.5,7.0) | 4.0 |
| Madhya Pradesh | Seoni | 40.3(39.8,40.9) | 77.6(77.1,78.0) | 37.2 | 25.9(25.4,26.4) | 46.6(46.1,47.2) | 20.8 | 5.0(4.7,5.2) | 9.4(9.1,9.8) | 4.4 |
| Madhya Pradesh | Balaghat | 45.6(45.1,46.1) | 80.7(80.3,81.1) | 35.1 | 39.1(38.6,39.6) | 52.8(52.3,53.3) | 13.7 | 5.0(4.8,5.2) | 12.0(11.7,12.3) | 7.0 |
| Madhya Pradesh | Guna | 35.1(34.5,35.7) | 74.7(74.1,75.2) | 39.6 | 32.9(32.4,33.5) | 36.0(35.4,36.6) | 3.0 | 3.8(3.5,4.0) | 4.9(4.6,5.2) | 1.1 |
| Madhya Pradesh | Ashoknagar | 28.8(28.1,29.5) | 72.2(71.5,72.9) | 43.4 | 32.7(31.9,33.4) | 46.7(45.9,47.4) | 14.0 | 3.4(3.1,3.7) | 5.6(5.2,6.0) | 2.2 |
| Madhya Pradesh | Shahdol | 36.2(35.6,36.9) | 75.9(75.3,76.4) | 39.6 | 26.1(25.6,26.7) | 50.6(49.9,51.2) | 24.4 | 2.4(2.2,2.6) | 9.6(9.2,10.0) | 7.2 |
| Madhya Pradesh | Anuppur | 41.1(40.4,41.9) | 76.1(75.4,76.7) | 35.0 | 25.8(25.1,26.5) | 50.8(50.1,51.6) | 25.0 | 3.8(3.5,4.1) | 9.1(8.7,9.6) | 5.3 |
| Madhya Pradesh | Sidhi | 31.3(30.7,31.9) | 72.2(71.6,72.8) | 40.9 | 27.9(27.3,28.5) | 47.8(47.2,48.5) | 19.9 | 2.9(2.6,3.1) | 6.3(6.0,6.6) | 3.5 |
| Madhya Pradesh | Singrauli | 29.6(29.0,30.2) | 72.1(71.6,72.7) | 42.5 | 25.1(24.5,25.7) | 47.3(46.6,48.0) | 22.2 | 2.0(1.8,2.2) | 8.7(8.3,9.1) | 6.7 |
| Madhya Pradesh | Jhabua | 32.0(31.3,32.7) | 71.1(70.5,71.8) | 39.1 | 31.3(30.6,31.9) | 45.6(44.9,46.4) | 14.4 | 2.4(2.2,2.6) | 6.9(6.6,7.3) | 4.5 |
| Madhya Pradesh | Alirajpur | 32.9(32.1,33.7) | 76.3(75.6,77.0) | 43.4 | 28.7(28.0,29.5) | 49.2(48.3,50.0) | 20.4 | 2.9(2.7,3.2) | 9.2(8.7,9.7) | 6.2 |
| Madhya Pradesh | East Nimar | 40.6(40.0,41.2) | 78.8(78.3,79.3) | 38.2 | 28.5(28.0,29.0) | 33.1(32.5,33.7) | 4.6 | 3.6(3.4,3.8) | 4.7(4.4,4.9) | 1.1 |
| Madhya Pradesh | Burhanpur | 37.8(37.0,38.5) | 75.0(74.3,75.7) | 37.2 | 37.7(36.9,38.4) | 48.7(47.9,49.5) | 11.0 | 4.1(3.8,4.4) | 7.4(7.0,7.9) | 3.3 |
| Gujarat | Kachchh | 52.9(52.4,53.4) | 75.1(74.7,75.5) | 22.2 | 38.2(37.7,38.7) | 40.2(39.7,40.6) | 2.0 | 3.4(3.2,3.6) | 3.1(3.0,3.3) | -0.3 |
| Gujarat | Banas Kantha | 34.0(33.7,34.4) | 65.6(65.2,66.0) | 31.6 | 29.2(28.8,29.5) | 32.5(32.1,32.8) | 3.3 | 1.8(1.7,1.9) | 2.3(2.2,2.4) | 0.5 |
| Gujarat | Patan | 41.5(40.9,42.1) | 72.6(72.0,73.1) | 31.0 | 35.7(35.1,36.2) | 51.4(50.9,52.0) | 15.7 | 3.1(2.9,3.3) | 6.2(5.9,6.5) | 3.1 |
| Gujarat | Mahesana | 48.4(48.0,48.9) | 71.3(70.9,71.7) | 22.8 | 42.1(41.7,42.6) | 45.1(44.6,45.5) | 2.9 | 3.1(3.0,3.3) | 3.6(3.5,3.8) | 0.5 |
| Gujarat | Sabar Kantha | 44.9(44.5,45.3) | 71.4(71.0,71.8) | 26.5 | 34.5(34.0,34.9) | 39.1(38.6,39.5) | 4.6 | 4.1(4.0,4.3) | 4.2(4.0,4.4) | 0.1 |
| Gujarat | Gandhinagar | 43.2(42.7,43.8) | 71.2(70.7,71.7) | 28.0 | 44.5(43.9,45.0) | 39.4(38.8,39.9) | -5.1 | 3.9(3.7,4.1) | 4.5(4.3,4.7) | 0.6 |
| Gujarat | Ahmadabad | 51.8(51.6,52.1) | 69.3(69.0,69.5) | 17.4 | 52.7(52.5,53.0) | 50.7(50.4,50.9) | -2.1 | 3.6(3.5,3.7) | 3.2(3.2,3.3) | -0.4 |
| Gujarat | Surendranagar | 46.2(45.7,46.7) | 68.9(68.4,69.4) | 22.7 | 37.8(37.3,38.3) | 40.8(40.3,41.3) | 3.1 | 2.7(2.5,2.8) | 3.9(3.7,4.1) | 1.2 |
| Gujarat | Rajkot | 58.5(58.2,58.9) | 68.5(68.2,68.8) | 10.0 | 43.7(43.3,44.0) | 39.0(38.7,39.3) | -4.6 | 2.8(2.7,2.9) | 4.3(4.2,4.5) | 1.5 |
| Gujarat | Jamnagar | 53.3(52.9,53.8) | 65.4(65.0,65.8) | 12.0 | 36.5(36.1,36.9) | 42.6(42.2,43.1) | 6.1 | 2.4(2.3,2.5) | 3.3(3.1,3.5) | 0.9 |
| Gujarat | Porbandar | 50.2(49.4,51.1) | 73.0(72.2,73.7) | 22.7 | 36.3(35.5,37.1) | 43.1(42.3,44.0) | 6.9 | 3.4(3.1,3.7) | 5.0(4.6,5.4) | 1.6 |
| Gujarat | Junagadh | 52.2(51.8,52.6) | 71.7(71.3,72.1) | 19.5 | 36.7(36.4,37.1) | 37.1(36.7,37.5) | 0.4 | 3.2(3.1,3.4) | 3.7(3.6,3.8) | 0.5 |
| Gujarat | Amreli | 50.3(49.8,50.8) | 70.9(70.4,71.3) | 20.6 | 37.1(36.6,37.6) | 38.6(38.1,39.1) | 1.5 | 2.9(2.8,3.1) | 4.3(4.1,4.6) | 1.4 |
| Gujarat | Bhavnagar | 44.9(44.5,45.3) | 61.9(61.5,62.2) | 17.0 | 36.5(36.1,36.8) | 31.1(30.7,31.5) | -5.4 | 2.5(2.4,2.7) | 3.6(3.5,3.8) | 1.1 |
| Gujarat | Anand | 46.8(46.4,47.3) | 73.9(73.5,74.2) | 27.0 | 40.1(39.6,40.5) | 48.8(48.4,49.3) | 8.8 | 4.6(4.4,4.7) | 7.3(7.1,7.5) | 2.7 |
| Gujarat | kheda | 40.5(40.1,40.9) | 61.3(60.9,61.7) | 20.8 | 32.6(32.2,33.0) | 35.1(34.7,35.5) | 2.5 | 3.5(3.3,3.6) | 5.1(4.9,5.3) | 1.6 |
| Gujarat | Panch Mahals | 41.3(40.9,41.7) | 70.5(70.1,70.9) | 29.2 | 31.8(31.4,32.2) | 39.4(39.0,39.9) | 7.7 | 3.0(2.9,3.2) | 3.8(3.7,4.0) | 0.8 |
| Gujarat | Dohad | 42.3(41.8,42.8) | 66.1(65.7,66.6) | 23.8 | 19.3(18.9,19.6) | 28.4(28.0,28.8) | 9.1 | 1.9(1.8,2.1) | 2.1(2.0,2.2) | 0.2 |
| Gujarat | Vadodara | 57.3(57.0,57.6) | 68.1(67.8,68.4) | 10.8 | 35.1(34.8,35.4) | 44.0(43.7,44.3) | 8.9 | 2.4(2.3,2.5) | 3.2(3.1,3.3) | 0.8 |
| Gujarat | Narmada | 55.2(54.3,56.0) | 73.1(72.4,73.9) | 18.0 | 40.8(40.0,41.7) | 44.2(43.3,45.0) | 3.4 | 3.5(3.2,3.8) | 4.8(4.4,5.1) | 1.3 |
| Gujarat | Bharuch | 49.2(48.7,49.8) | 66.8(66.3,67.3) | 17.5 | 32.2(31.8,32.7) | 33.7(33.2,34.2) | 1.5 | 2.4(2.2,2.6) | 3.8(3.6,4.0) | 1.4 |
| Gujarat | Dang | 46.3(44.9,47.8) | 74.9(73.6,76.1) | 28.6 | 25.3(24.1,26.6) | 39.3(37.9,40.7) | 13.9 | 1.9(1.5,2.3) | 3.7(3.2,4.3) | 1.8 |
| Gujarat | Navsari | 59.7(59.2,60.2) | 81.7(81.3,82.2) | 22.1 | 46.1(45.6,46.7) | 50.4(49.8,50.9) | 4.2 | 4.7(4.5,4.9) | 6.6(6.3,6.9) | 1.9 |
| Gujarat | Valsad | 55.3(54.8,55.8) | 78.8(78.3,79.2) | 23.5 | 45.8(45.3,46.3) | 47.2(46.7,47.7) | 1.4 | 4.6(4.4,4.8) | 5.6(5.4,5.8) | 1.0 |
| Gujarat | Surat | 54.4(54.1,54.7) | 65.9(65.6,66.2) | 11.5 | 47.4(47.1,47.6) | 43.8(43.5,44.1) | -3.6 | 2.9(2.8,3.0) | 5.2(5.1,5.3) | 2.3 |
| Gujarat | Tapi | 51.4(50.7,52.1) | 76.3(75.7,76.9) | 24.9 | 30.5(29.8,31.1) | 34.0(33.3,34.7) | 3.5 | 3.9(3.6,4.1) | 5.4(5.1,5.7) | 1.5 |
| Daman & Diu | Diu | 63.4(60.7,66.2) | 88.0(86.1,89.8) | 24.5 | 31.4(28.8,34.1) | 43.9(41.1,46.7) | 12.5 | 1.6(0.9,2.3) | 6.2(4.8,7.5) | 4.6 |
| Daman & Diu | Daman | 52.7(50.9,54.4) | 80.9(79.5,82.2) | 28.2 | 23.2(21.8,24.7) | 40.2(38.5,41.9) | 16.9 | 3.4(2.8,4.1) | 4.9(4.2,5.7) | 1.5 |
| Dadra & Nagar Haveli | Dadra and Nagar Haveli | 51.7(50.5,53.0) | 86.1(85.2,86.9) | 34.3 | 12.4(11.5,13.2) | 32.4(31.3,33.6) | 20.1 | 0.0(0.0,0.0) | 3.5(3.0,3.9) | 3.5 |
| Maharashtra | Nandurbar | 39.0(38.5,39.5) | 61.9(61.4,62.4) | 23.0 | 28.8(28.4,29.3) | 41.6(41.1,42.2) | 12.8 | 4.2(4.0,4.4) | 5.4(5.2,5.7) | 1.3 |
| Maharashtra | Dhule | 39.6(39.1,40.0) | 65.7(65.3,66.2) | 26.1 | 35.7(35.2,36.1) | 49.1(48.6,49.6) | 13.4 | 7.1(6.9,7.4) | 10.0(9.7,10.3) | 2.9 |
| Maharashtra | Jalgaon | 38.0(37.7,38.4) | 67.8(67.5,68.1) | 29.8 | 36.9(36.6,37.2) | 46.0(45.7,46.3) | 9.1 | 6.6(6.4,6.7) | 10.0(9.8,10.2) | 3.4 |
| Maharashtra | Buldhana | 39.8(39.4,40.2) | 72.4(72.1,72.8) | 32.6 | 36.4(36.0,36.8) | 44.1(43.7,44.5) | 7.7 | 4.8(4.6,5.0) | 7.2(7.0,7.5) | 2.4 |
| Maharashtra | Akola | 43.5(43.0,43.9) | 74.0(73.6,74.4) | 30.5 | 40.7(40.2,41.2) | 49.3(48.8,49.8) | 8.6 | 7.5(7.2,7.7) | 14.2(13.9,14.5) | 6.7 |
| Maharashtra | Washim | 42.1(41.5,42.7) | 74.4(73.9,75.0) | 32.3 | 40.3(39.7,40.9) | 51.0(50.4,51.6) | 10.7 | 8.0(7.7,8.4) | 11.6(11.2,11.9) | 3.5 |
| Maharashtra | Amravati | 44.2(43.8,44.5) | 75.2(74.9,75.5) | 31.0 | 40.1(39.7,40.4) | 50.0(49.6,50.4) | 9.9 | 8.5(8.3,8.7) | 13.8(13.6,14.1) | 5.3 |
| Maharashtra | Wardha | 48.5(47.9,49.0) | 72.7(72.2,73.2) | 24.2 | 37.0(36.5,37.5) | 44.8(44.3,45.4) | 7.8 | 7.0(6.7,7.3) | 7.6(7.3,7.9) | 0.6 |
| Maharashtra | Nagpur | 55.9(55.6,56.2) | 69.3(69.0,69.5) | 13.4 | 44.2(43.9,44.5) | 48.6(48.3,48.9) | 4.4 | 5.1(5.0,5.3) | 7.0(6.8,7.1) | 1.8 |
| Maharashtra | Bhandara | 44.0(43.4,44.6) | 76.4(75.9,76.9) | 32.4 | 34.3(33.7,34.8) | 46.4(45.8,47.0) | 12.1 | 6.7(6.4,7.0) | 7.9(7.6,8.3) | 1.2 |
| Maharashtra | Gondia | 49.2(48.6,49.7) | 80.3(79.9,80.7) | 31.1 | 30.3(29.8,30.8) | 46.5(45.9,47.0) | 16.2 | 5.0(4.8,5.2) | 7.5(7.2,7.8) | 2.5 |
| Maharashtra | Gadchiroli | 43.7(43.1,44.3) | 70.4(69.9,71.0) | 26.7 | 27.2(26.7,27.8) | 48.9(48.3,49.5) | 21.7 | 5.4(5.1,5.7) | 12.0(11.5,12.4) | 6.6 |
| Maharashtra | Chandrapur | 44.4(44.0,44.8) | 69.9(69.5,70.3) | 25.5 | 36.7(36.3,37.1) | 49.7(49.3,50.1) | 13.0 | 9.9(9.6,10.1) | 10.0(9.8,10.3) | 0.1 |
| Maharashtra | Yavatmal | 41.8(41.4,42.2) | 73.0(72.6,73.3) | 31.1 | 35.8(35.4,36.2) | 43.9(43.5,44.2) | 8.1 | 6.5(6.3,6.7) | 7.8(7.6,8.1) | 1.4 |
| Maharashtra | Nanded | 35.9(35.5,36.2) | 67.0(66.7,67.4) | 31.2 | 36.6(36.2,36.9) | 46.7(46.3,47.1) | 10.1 | 5.1(5.0,5.3) | 7.9(7.7,8.1) | 2.8 |
| Maharashtra | Hingoli | 40.8(40.2,41.4) | 69.0(68.4,69.5) | 28.2 | 35.3(34.7,35.9) | 44.9(44.3,45.5) | 9.5 | 3.9(3.7,4.1) | 7.9(7.6,8.3) | 4.1 |
| Maharashtra | Parbhani | 34.4(33.9,34.8) | 64.4(63.9,64.8) | 30.0 | 37.6(37.1,38.0) | 40.5(40.0,40.9) | 2.9 | 4.5(4.3,4.7) | 8.1(7.8,8.4) | 3.6 |
| Maharashtra | Jalna | 38.9(38.4,39.3) | 65.0(64.5,65.4) | 26.1 | 30.6(30.1,31.0) | 54.1(53.6,54.5) | 23.5 | 4.3(4.1,4.5) | 9.5(9.3,9.8) | 5.2 |
| Maharashtra | Aurangabad | 44.8(44.5,45.2) | 73.8(73.5,74.1) | 28.9 | 36.9(36.6,37.2) | 42.0(41.7,42.4) | 5.1 | 4.1(3.9,4.2) | 7.2(7.1,7.4) | 3.2 |
| Maharashtra | Nashik | 46.2(45.9,46.4) | 75.2(75.0,75.4) | 29.0 | 34.2(33.9,34.4) | 49.9(49.6,50.1) | 15.7 | 5.2(5.1,5.3) | 7.4(7.3,7.5) | 2.2 |
| Maharashtra | Thane | 50.1(49.9,50.3) | 73.3(73.1,73.5) | 23.2 | 38.7(38.5,38.9) | 50.4(50.2,50.6) | 11.7 | 8.7(8.5,8.8) | 7.6(7.5,7.7) | -1.1 |
| Maharashtra | Mumbai Suburban | 48.5(48.3,48.7) | 72.6(72.4,72.8) | 24.1 | 34.4(34.2,34.6) | 52.5(52.3,52.8) | 18.1 | 2.2(2.1,2.2) | 3.7(3.6,3.7) | 1.5 |
| Maharashtra | Mumbai | 49.0(48.6,49.4) | 72.7(72.4,73.1) | 23.7 | 31.7(31.4,32.1) | 52.2(51.8,52.6) | 20.5 | 2.0(1.9,2.1) | 3.6(3.4,3.7) | 1.6 |
| Maharashtra | Raigad | 47.1(46.7,47.5) | 69.1(68.7,69.5) | 22.0 | 33.3(32.9,33.6) | 54.0(53.6,54.4) | 20.7 | 4.4(4.3,4.6) | 6.6(6.4,6.8) | 2.2 |
| Maharashtra | Pune | 48.9(48.7,49.2) | 73.9(73.7,74.1) | 25.0 | 49.1(48.9,49.3) | 60.0(59.8,60.2) | 10.9 | 6.5(6.4,6.6) | 7.6(7.5,7.8) | 1.1 |
| Maharashtra | Ahmednagar | 44.0(43.7,44.3) | 71.1(70.8,71.4) | 27.1 | 38.5(38.2,38.8) | 46.3(46.0,46.6) | 7.7 | 3.2(3.1,3.3) | 9.3(9.1,9.4) | 6.0 |
| Maharashtra | Beed | 36.5(36.1,36.9) | 68.9(68.5,69.2) | 32.4 | 35.8(35.4,36.2) | 43.0(42.6,43.4) | 7.2 | 4.2(4.0,4.4) | 6.4(6.2,6.6) | 2.2 |
| Maharashtra | Latur | 34.9(34.5,35.3) | 61.0(60.5,61.4) | 26.1 | 31.3(30.9,31.7) | 39.1(38.7,39.5) | 7.8 | 4.4(4.3,4.6) | 8.3(8.1,8.6) | 3.9 |
| Maharashtra | Osmanabad | 40.1(39.6,40.6) | 70.3(69.9,70.8) | 30.2 | 36.6(36.1,37.1) | 56.7(56.2,57.2) | 20.2 | 8.6(8.3,8.9) | 14.8(14.4,15.1) | 6.2 |
| Maharashtra | Solapur | 41.2(40.9,41.5) | 74.3(74.0,74.6) | 33.1 | 38.1(37.8,38.4) | 60.1(59.8,60.4) | 22.0 | 5.2(5.1,5.4) | 14.4(14.1,14.6) | 9.1 |
| Maharashtra | Satara | 47.0(46.7,47.4) | 73.1(72.7,73.4) | 26.0 | 36.5(36.2,36.9) | 52.8(52.4,53.2) | 16.3 | 3.4(3.2,3.5) | 9.8(9.6,10.0) | 6.4 |
| Maharashtra | Ratnagiri | 46.8(46.4,47.3) | 75.6(75.2,76.0) | 28.7 | 31.9(31.5,32.4) | 53.6(53.1,54.1) | 21.6 | 7.5(7.3,7.8) | 10.3(10.0,10.6) | 2.8 |
| Maharashtra | Sindhudurg | 40.9(40.2,41.5) | 81.0(80.5,81.6) | 40.2 | 35.6(34.9,36.2) | 61.9(61.3,62.6) | 26.4 | 5.5(5.2,5.8) | 13.1(12.7,13.6) | 7.6 |
| Maharashtra | Kolhapur | 47.5(47.2,47.8) | 74.5(74.2,74.8) | 27.0 | 38.9(38.6,39.2) | 56.3(56.0,56.6) | 17.4 | 7.9(7.7,8.0) | 8.1(7.9,8.3) | 0.2 |
| Maharashtra | Sangli | 45.7(45.3,46.1) | 73.6(73.3,73.9) | 27.9 | 33.9(33.5,34.2) | 50.1(49.7,50.5) | 16.2 | 5.1(5.0,5.3) | 9.5(9.2,9.7) | 4.3 |
| Telangana | Adilabad | 55.5(55.1,55.8) | 79.3(79.0,79.6) | 23.8 | 61.9(61.5,62.2) | 59.1(58.7,59.5) | -2.8 | 23.9(23.5,24.2) | 21.8(21.5,22.2) | -2.0 |
| Telangana | Nizamabad | 55.4(55.0,55.8) | 88.6(88.4,88.9) | 33.3 | 54.3(53.9,54.7) | 69.2(68.9,69.6) | 14.9 | 23.0(22.6,23.3) | 30.4(30.0,30.7) | 7.4 |
| Telangana | Karimnagar | 59.8(59.4,60.1) | 87.1(86.9,87.3) | 27.4 | 66.0(65.7,66.3) | 60.4(60.1,60.7) | -5.6 | 28.5(28.2,28.8) | 25.9(25.6,26.2) | -2.6 |
| Telangana | Medak | 55.5(55.1,55.8) | 83.1(82.9,83.4) | 27.7 | 58.6(58.2,59.0) | 60.8(60.5,61.2) | 2.2 | 27.8(27.5,28.1) | 25.9(25.6,26.2) | -1.9 |
| Telangana | Hyderabad | 58.9(58.5,59.2) | 86.9(86.7,87.1) | 28.0 | 50.9(50.5,51.2) | 58.1(57.8,58.4) | 7.3 | 9.0(8.8,9.2) | 9.5(9.3,9.7) | 0.5 |
| Telangana | Ranga Reddy | 64.5(64.3,64.8) | 82.2(82.0,82.4) | 17.7 | 65.1(64.8,65.3) | 56.6(56.3,56.8) | -8.5 | 18.9(18.7,19.1) | 18.2(17.9,18.4) | -0.8 |
| Telangana | Mahabubnagar | 51.8(51.5,52.2) | 82.5(82.2,82.7) | 30.7 | 46.3(45.9,46.6) | 61.5(61.2,61.8) | 15.2 | 18.4(18.2,18.7) | 26.2(25.9,26.5) | 7.8 |
| Telangana | Nalgonda | 60.1(59.7,60.4) | 85.7(85.5,85.9) | 25.6 | 62.4(62.1,62.8) | 66.0(65.6,66.3) | 3.5 | 29.9(29.6,30.2) | 31.1(30.8,31.4) | 1.2 |
| Telangana | Warangal | 60.9(60.6,61.2) | 81.5(81.2,81.8) | 20.6 | 66.4(66.0,66.7) | 62.4(62.1,62.8) | -3.9 | 28.0(27.7,28.3) | 24.2(23.9,24.4) | -3.8 |
| Telangana | Khammam | 61.9(61.5,62.2) | 88.2(88.0,88.5) | 26.4 | 60.0(59.7,60.4) | 64.1(63.7,64.4) | 4.0 | 34.5(34.1,34.8) | 28.4(28.0,28.7) | -6.1 |
| Andhra Pradesh | Srikakulam | 64.7(64.3,65.0) | 80.7(80.4,81.0) | 16.0 | 67.8(67.4,68.2) | 61.8(61.5,62.2) | -6.0 | 32.6(32.2,33.0) | 32.9(32.6,33.3) | 0.3 |
| Andhra Pradesh | Vizianagaram | 62.5(62.1,62.9) | 81.8(81.5,82.2) | 19.4 | 67.1(66.8,67.5) | 59.8(59.4,60.2) | -7.4 | 32.6(32.2,32.9) | 29.9(29.6,30.3) | -2.6 |
| Andhra Pradesh | Visakhapatanam | 67.5(67.2,67.8) | 85.9(85.7,86.1) | 18.4 | 76.8(76.5,77.0) | 66.9(66.6,67.2) | -9.9 | 32.7(32.4,33.0) | 31.8(31.5,32.1) | -0.9 |
| Andhra Pradesh | East Godavari | 65.7(65.5,66.0) | 80.9(80.7,81.1) | 15.2 | 69.9(69.7,70.2) | 61.0(60.8,61.3) | -8.9 | 26.7(26.5,27.0) | 26.5(26.3,26.7) | -0.2 |
| Andhra Pradesh | West Godavari | 70.3(70.0,70.6) | 87.5(87.3,87.7) | 17.2 | 72.9(72.6,73.2) | 69.2(68.9,69.5) | -3.8 | 33.0(32.7,33.3) | 35.9(35.6,36.2) | 2.9 |
| Andhra Pradesh | Krishna | 72.4(72.2,72.7) | 86.6(86.4,86.8) | 14.2 | 77.3(77.0,77.5) | 69.8(69.5,70.1) | -7.5 | 38.9(38.6,39.2) | 41.8(41.5,42.1) | 2.9 |
| Andhra Pradesh | Guntur | 70.4(70.1,70.7) | 82.8(82.6,83.0) | 12.4 | 78.9(78.7,79.2) | 61.9(61.6,62.2) | -17.1 | 31.3(31.0,31.6) | 31.8(31.5,32.0) | 0.5 |
| Andhra Pradesh | Prakasam | 68.1(67.8,68.4) | 80.8(80.5,81.0) | 12.7 | 73.0(72.7,73.4) | 64.7(64.4,65.0) | -8.3 | 33.1(32.8,33.4) | 33.6(33.3,34.0) | 0.5 |
| Andhra Pradesh | SPR Nellore | 65.7(65.4,66.1) | 80.8(80.6,81.1) | 15.1 | 67.9(67.6,68.2) | 57.0(56.7,57.4) | -10.9 | 27.2(26.8,27.5) | 20.9(20.6,21.2) | -6.3 |
| Andhra Pradesh | Y.S.R. | 69.3(69.0,69.7) | 81.5(81.2,81.8) | 12.2 | 66.9(66.6,67.3) | 55.8(55.4,56.2) | -11.1 | 22.5(22.2,22.8) | 21.4(21.1,21.7) | -1.1 |
| Andhra Pradesh | Kurnool | 63.1(62.8,63.4) | 74.1(73.9,74.4) | 11.1 | 67.5(67.2,67.8) | 50.4(50.1,50.8) | -17.1 | 24.0(23.7,24.2) | 26.4(26.1,26.7) | 2.4 |
| Andhra Pradesh | Anantapur | 66.0(65.7,66.3) | 84.5(84.3,84.8) | 18.5 | 70.9(70.7,71.2) | 70.0(69.7,70.3) | -0.9 | 26.8(26.5,27.1) | 35.8(35.5,36.1) | 9.0 |
| Andhra Pradesh | Chittoor | 67.9(67.6,68.2) | 84.4(84.1,84.6) | 16.5 | 64.1(63.8,64.4) | 56.1(55.8,56.4) | -8.0 | 28.4(28.1,28.6) | 25.0(24.7,25.3) | -3.4 |
| Karnataka | Belagavi | 55.7(55.4,56.0) | 87.6(87.4,87.8) | 31.9 | 40.2(39.9,40.5) | 53.0(52.7,53.3) | 12.8 | 8.8(8.7,9.0) | 16.4(16.2,16.6) | 7.5 |
| Karnataka | Bagalkote | 49.3(48.8,49.8) | 84.9(84.6,85.3) | 35.6 | 32.0(31.5,32.4) | 59.2(58.7,59.7) | 27.2 | 8.8(8.5,9.0) | 17.7(17.3,18.1) | 8.9 |
| Karnataka | Bijapur | 48.2(47.8,48.7) | 85.3(84.9,85.6) | 37.0 | 30.8(30.4,31.2) | 57.6(57.2,58.1) | 26.9 | 9.5(9.2,9.8) | 17.6(17.2,17.9) | 8.1 |
| Karnataka | Bidar | 47.6(47.1,48.1) | 86.6(86.2,86.9) | 39.0 | 34.4(34.0,34.9) | 47.4(46.9,47.9) | 13.0 | 8.8(8.5,9.1) | 12.6(12.3,13.0) | 3.8 |
| Karnataka | Raichur | 44.4(44.0,44.9) | 88.7(88.4,89.0) | 44.3 | 34.3(33.8,34.7) | 56.5(56.0,57.0) | 22.2 | 7.5(7.2,7.8) | 18.8(18.4,19.1) | 11.3 |
| Karnataka | Koppal | 50.3(49.8,50.9) | 86.1(85.7,86.5) | 35.8 | 29.4(28.9,29.9) | 58.6(58.0,59.1) | 29.2 | 8.9(8.6,9.3) | 18.1(17.7,18.6) | 9.2 |
| Karnataka | Gadag | 53.7(53.1,54.3) | 82.9(82.4,83.3) | 29.2 | 45.2(44.6,45.8) | 55.6(55.0,56.2) | 10.4 | 13.1(12.7,13.6) | 19.1(18.7,19.6) | 6.0 |
| Karnataka | Dharwad | 59.0(58.6,59.5) | 90.1(89.8,90.4) | 31.1 | 38.5(38.0,38.9) | 58.3(57.9,58.8) | 19.9 | 11.4(11.1,11.7) | 13.9(13.6,14.2) | 2.5 |
| Karnataka | Uttara Kannada | 61.9(61.4,62.5) | 89.8(89.5,90.1) | 27.9 | 37.1(36.6,37.6) | 55.5(54.9,56.0) | 18.4 | 13.1(12.7,13.4) | 16.6(16.2,17.0) | 3.5 |
| Karnataka | Haveri | 51.6(51.1,52.2) | 88.6(88.2,88.9) | 36.9 | 34.8(34.3,35.3) | 55.8(55.3,56.4) | 21.0 | 8.6(8.3,8.8) | 17.0(16.6,17.4) | 8.4 |
| Karnataka | Ballari | 49.9(49.5,50.3) | 84.7(84.4,85.0) | 34.8 | 34.3(33.9,34.7) | 48.1(47.6,48.5) | 13.8 | 10.3(10.0,10.5) | 10.6(10.4,10.9) | 0.4 |
| Karnataka | Chitradurga | 55.5(55.0,56.0) | 90.8(90.5,91.1) | 35.2 | 32.2(31.7,32.7) | 61.2(60.7,61.7) | 29.0 | 10.9(10.6,11.2) | 20.1(19.7,20.4) | 9.2 |
| Karnataka | Davangere | 53.9(53.5,54.4) | 90.2(90.0,90.5) | 36.3 | 39.7(39.2,40.1) | 61.3(60.9,61.8) | 21.7 | 12.4(12.1,12.7) | 16.9(16.6,17.3) | 4.5 |
| Karnataka | Shivamogga | 57.1(56.7,57.6) | 86.9(86.6,87.2) | 29.7 | 38.1(37.6,38.5) | 50.8(50.3,51.3) | 12.7 | 13.7(13.3,14.0) | 19.4(19.0,19.8) | 5.7 |
| Karnataka | Udupi | 68.6(68.1,69.1) | 92.7(92.4,93.0) | 24.1 | 36.8(36.3,37.4) | 65.3(64.8,65.8) | 28.5 | 14.4(14.0,14.8) | 14.6(14.2,15.0) | 0.2 |
| Karnataka | Chikkamagaluru | 63.4(62.9,64.0) | 91.2(90.9,91.6) | 27.8 | 41.6(41.0,42.2) | 62.4(61.8,63.0) | 20.8 | 14.7(14.3,15.2) | 20.7(20.3,21.2) | 6.0 |
| Karnataka | Tumkur | 54.9(54.6,55.3) | 90.0(89.8,90.2) | 35.0 | 42.7(42.3,43.1) | 62.6(62.3,63.0) | 19.9 | 12.7(12.5,13.0) | 24.3(24.0,24.7) | 11.6 |
| Karnataka | Bangalore Urban | 74.9(74.7,75.1) | 93.7(93.6,93.8) | 18.8 | 49.6(49.4,49.8) | 58.3(58.1,58.5) | 8.7 | 21.6(21.5,21.8) | 18.4(18.2,18.5) | -3.3 |
| Karnataka | Mandya | 54.7(54.3,55.2) | 90.8(90.6,91.1) | 36.1 | 37.9(37.5,38.4) | 64.9(64.4,65.3) | 27.0 | 14.0(13.7,14.4) | 21.4(21.0,21.8) | 7.4 |
| Karnataka | Hassan | 67.6(67.2,68.1) | 90.3(90.0,90.6) | 22.7 | 45.2(44.8,45.7) | 57.4(56.9,57.8) | 12.1 | 13.6(13.3,13.9) | 25.7(25.3,26.2) | 12.1 |
| Karnataka | Dakshina Kannada | 69.1(68.7,69.5) | 94.4(94.2,94.6) | 25.3 | 37.6(37.1,38.0) | 62.2(61.8,62.7) | 24.7 | 17.7(17.4,18.0) | 16.2(15.9,16.5) | -1.5 |
| Karnataka | Kodagu | 66.4(65.6,67.2) | 90.9(90.4,91.4) | 24.5 | 39.6(38.7,40.4) | 57.3(56.5,58.1) | 17.7 | 14.2(13.6,14.8) | 23.3(22.6,24.0) | 9.1 |
| Karnataka | Mysuru | 61.6(61.3,62.0) | 92.9(92.7,93.1) | 31.3 | 40.7(40.3,41.0) | 64.0(63.7,64.4) | 23.4 | 15.7(15.4,16.0) | 18.5(18.3,18.8) | 2.8 |
| Karnataka | Chamarajanagara | 50.5(49.9,51.1) | 88.2(87.8,88.6) | 37.7 | 40.3(39.7,40.9) | 59.6(59.0,60.2) | 19.3 | 13.4(13.0,13.9) | 30.0(29.4,30.6) | 16.6 |
| Karnataka | Gulbarga | 47.5(47.1,47.9) | 87.9(87.7,88.2) | 40.4 | 30.6(30.2,31.0) | 56.1(55.6,56.5) | 25.4 | 7.8(7.6,8.0) | 13.9(13.6,14.2) | 6.1 |
| Karnataka | Yadgir | 39.0(38.4,39.6) | 81.1(80.6,81.6) | 42.1 | 24.6(24.1,25.1) | 51.2(50.6,51.8) | 26.6 | 5.8(5.5,6.1) | 17.7(17.2,18.2) | 11.9 |
| Karnataka | Kolar | 59.1(58.6,59.6) | 88.7(88.3,89.0) | 29.6 | 38.9(38.4,39.4) | 55.5(55.0,56.0) | 16.5 | 8.1(7.8,8.4) | 15.4(15.1,15.8) | 7.4 |
| Karnataka | Chikkaballapura | 54.7(54.2,55.3) | 89.1(88.7,89.4) | 34.3 | 35.9(35.3,36.4) | 55.5(54.9,56.1) | 19.6 | 12.0(11.7,12.4) | 17.6(17.1,18.0) | 5.5 |
| Karnataka | Bangalore Rural | 62.1(61.5,62.7) | 93.6(93.2,93.9) | 31.4 | 42.5(41.9,43.1) | 66.1(65.5,66.7) | 23.6 | 14.5(14.1,15.0) | 20.5(19.9,21.0) | 5.9 |
| Karnataka | Ramanagara | 53.0(52.4,53.6) | 90.6(90.2,90.9) | 37.5 | 40.3(39.7,40.9) | 63.4(62.8,64.0) | 23.0 | 15.7(15.2,16.1) | 31.0(30.4,31.5) | 15.3 |
| Goa | North Goa | 84.1(83.6,84.6) | 89.2(88.8,89.6) | 5.1 | 54.4(53.7,55.1) | 76.0(75.4,76.5) | 21.5 | 5.6(5.3,5.9) | 11.9(11.5,12.4) | 6.3 |
| Goa | South Goa | 81.0(80.4,81.7) | 87.5(86.9,88.0) | 6.4 | 52.2(51.4,53.0) | 74.4(73.7,75.1) | 22.2 | 2.6(2.3,2.8) | 8.0(7.6,8.4) | 5.4 |
| Lakshadweep | Lakshadweep | 74.4(72.2,76.6) | 66.9(64.5,69.3) | -7.5 | 62.3(59.8,64.7) | 45.2(42.6,47.7) | -17.1 | 0.5(0.1,0.8) | 1.5(0.9,2.1) | 1.0 |
| Kerala | Kasaragod | 68.1(67.6,68.6) | 77.4(76.9,77.9) | 9.3 | 63.0(62.5,63.6) | 52.2(51.7,52.8) | -10.8 | 7.3(7.0,7.6) | 9.0(8.7,9.3) | 1.7 |
| Kerala | Kannur | 73.4(73.0,73.7) | 73.1(72.8,73.5) | -0.2 | 71.2(70.9,71.6) | 53.1(52.8,53.5) | -18.1 | 7.4(7.2,7.5) | 9.4(9.2,9.6) | 2.1 |
| Kerala | Wayanad | 65.5(64.8,66.1) | 78.8(78.2,79.3) | 13.3 | 67.1(66.4,67.7) | 59.8(59.2,60.5) | -7.3 | 7.0(6.7,7.4) | 12.2(11.7,12.6) | 5.1 |
| Kerala | Kozhikode | 71.1(70.8,71.4) | 78.4(78.1,78.7) | 7.3 | 71.0(70.7,71.3) | 60.3(59.9,60.6) | -10.7 | 11.1(10.9,11.3) | 7.8(7.7,8.0) | -3.3 |
| Kerala | Malappuram | 61.5(61.2,61.8) | 69.3(69.0,69.6) | 7.8 | 60.5(60.2,60.8) | 45.2(44.8,45.5) | -15.3 | 4.3(4.1,4.4) | 8.2(8.0,8.3) | 3.9 |
| Kerala | Palakkad | 66.9(66.6,67.3) | 78.9(78.6,79.2) | 12.0 | 69.5(69.1,69.8) | 62.3(62.0,62.7) | -7.1 | 9.5(9.3,9.7) | 10.5(10.3,10.7) | 1.0 |
| Kerala | Thrissur | 73.6(73.3,73.9) | 79.6(79.3,79.8) | 6.0 | 74.2(73.9,74.5) | 59.8(59.5,60.1) | -14.4 | 10.6(10.3,10.8) | 10.7(10.5,10.9) | 0.1 |
| Kerala | Ernakulam | 74.8(74.5,75.1) | 80.9(80.7,81.2) | 6.2 | 67.8(67.5,68.1) | 58.4(58.1,58.8) | -9.4 | 7.1(7.0,7.3) | 5.8(5.6,6.0) | -1.3 |
| Kerala | Idukki | 71.1(70.5,71.6) | 81.9(81.4,82.4) | 10.8 | 74.7(74.2,75.2) | 66.0(65.5,66.6) | -8.7 | 10.8(10.4,11.1) | 13.3(12.9,13.7) | 2.5 |
| Kerala | Kottayam | 72.2(71.8,72.6) | 81.9(81.6,82.2) | 9.7 | 76.2(75.9,76.6) | 62.5(62.0,62.9) | -13.8 | 13.5(13.2,13.8) | 12.3(12.0,12.6) | -1.2 |
| Kerala | Alappuzha | 73.7(73.3,74.1) | 78.6(78.2,78.9) | 4.9 | 68.5(68.1,68.9) | 58.1(57.7,58.5) | -10.3 | 13.8(13.5,14.1) | 10.6(10.3,10.8) | -3.3 |
| Kerala | Pathanamthitta | 74.8(74.3,75.3) | 78.9(78.5,79.4) | 4.1 | 77.1(76.7,77.6) | 63.4(62.9,64.0) | -13.7 | 8.9(8.6,9.2) | 14.4(14.0,14.8) | 5.5 |
| Kerala | Kollam | 73.2(72.9,73.5) | 81.5(81.2,81.8) | 8.3 | 67.6(67.3,68.0) | 58.6(58.2,59.0) | -9.0 | 15.4(15.2,15.7) | 8.8(8.6,9.1) | -6.6 |
| Kerala | Thiruvananthapuram | 80.7(80.4,80.9) | 79.2(78.9,79.5) | -1.5 | 67.1(66.7,67.4) | 58.8(58.5,59.1) | -8.3 | 14.7(14.5,14.9) | 12.2(12.0,12.4) | -2.5 |
| Tamil Nadu | Thiruvallur | 79.4(79.1,79.6) | 92.1(92.0,92.3) | 12.8 | 58.3(58.0,58.7) | 77.3(77.0,77.6) | 18.9 | 19.3(19.0,19.5) | 20.3(20.1,20.6) | 1.1 |
| Tamil Nadu | Chennai | 76.2(75.9,76.4) | 94.2(94.0,94.3) | 18.0 | 51.8(51.5,52.1) | 70.5(70.2,70.8) | 18.7 | 11.8(11.7,12.0) | 14.7(14.5,14.9) | 2.9 |
| Tamil Nadu | Kanchipuram | 78.8(78.6,79.1) | 92.0(91.8,92.2) | 13.2 | 49.1(48.7,49.4) | 69.8(69.6,70.1) | 20.8 | 15.9(15.7,16.1) | 16.0(15.7,16.2) | 0.1 |
| Tamil Nadu | Vellore | 74.6(74.3,74.9) | 95.0(94.9,95.2) | 20.4 | 47.7(47.4,48.0) | 76.8(76.5,77.1) | 29.1 | 18.6(18.4,18.9) | 16.7(16.4,16.9) | -2.0 |
| Tamil Nadu | Tiruvannamalai | 76.3(76.0,76.7) | 94.1(93.9,94.3) | 17.8 | 49.9(49.5,50.3) | 71.2(70.8,71.6) | 21.3 | 12.9(12.6,13.2) | 20.0(19.7,20.3) | 7.1 |
| Tamil Nadu | Villuppuram | 71.4(71.1,71.7) | 92.3(92.1,92.5) | 20.9 | 49.0(48.6,49.3) | 74.4(74.1,74.7) | 25.4 | 12.4(12.2,12.6) | 19.1(18.9,19.4) | 6.8 |
| Tamil Nadu | Salem | 73.5(73.2,73.8) | 89.5(89.3,89.7) | 16.0 | 45.5(45.2,45.8) | 73.5(73.2,73.8) | 28.0 | 12.2(11.9,12.4) | 15.8(15.6,16.1) | 3.6 |
| Tamil Nadu | Namakkal | 72.1(71.6,72.5) | 93.2(93.0,93.5) | 21.2 | 47.5(47.0,47.9) | 74.2(73.8,74.6) | 26.8 | 14.2(13.8,14.5) | 19.4(19.1,19.8) | 5.3 |
| Tamil Nadu | Erode | 73.4(73.0,73.8) | 93.0(92.8,93.2) | 19.6 | 45.9(45.5,46.3) | 73.9(73.5,74.3) | 28.0 | 17.5(17.2,17.9) | 19.5(19.2,19.8) | 1.9 |
| Tamil Nadu | The Nilgiris | 82.2(81.7,82.8) | 94.9(94.6,95.2) | 12.6 | 52.1(51.3,52.8) | 73.4(72.7,74.0) | 21.3 | 19.9(19.4,20.5) | 14.1(13.6,14.6) | -5.8 |
| Tamil Nadu | Dindigul | 74.9(74.6,75.3) | 94.0(93.8,94.2) | 19.0 | 42.6(42.2,43.0) | 76.5(76.2,76.9) | 33.9 | 16.8(16.5,17.1) | 23.1(22.7,23.4) | 6.3 |
| Tamil Nadu | Karur | 77.6(77.1,78.1) | 90.9(90.5,91.2) | 13.3 | 52.1(51.5,52.7) | 72.2(71.7,72.8) | 20.1 | 11.9(11.5,12.2) | 20.4(19.9,20.9) | 8.5 |
| Tamil Nadu | Tiruchirappalli | 80.3(80.0,80.6) | 91.8(91.6,92.0) | 11.5 | 54.5(54.1,54.9) | 73.7(73.4,74.0) | 19.2 | 13.1(12.8,13.3) | 20.6(20.3,20.9) | 7.5 |
| Tamil Nadu | Perambalur | 80.9(80.3,81.6) | 95.4(95.0,95.7) | 14.4 | 48.7(47.8,49.5) | 73.5(72.8,74.2) | 24.8 | 13.5(12.9,14.0) | 20.3(19.6,20.9) | 6.8 |
| Tamil Nadu | Ariyalur | 77.3(76.7,77.9) | 93.5(93.2,93.9) | 16.2 | 53.7(53.0,54.4) | 78.5(78.0,79.1) | 24.8 | 18.4(17.8,19.0) | 21.6(21.0,22.2) | 3.2 |
| Tamil Nadu | Cuddalore | 73.4(73.0,73.7) | 91.0(90.7,91.2) | 17.6 | 48.1(47.8,48.5) | 67.5(67.1,67.9) | 19.3 | 14.9(14.6,15.2) | 16.5(16.2,16.8) | 1.6 |
| Tamil Nadu | Nagapattinam | 83.5(83.1,83.9) | 94.4(94.2,94.7) | 10.9 | 54.1(53.6,54.6) | 76.5(76.1,76.9) | 22.4 | 17.1(16.7,17.5) | 25.2(24.8,25.6) | 8.1 |
| Tamil Nadu | Thiruvarur | 77.7(77.3,78.2) | 95.0(94.8,95.2) | 17.3 | 43.8(43.2,44.3) | 84.9(84.5,85.3) | 41.2 | 18.8(18.3,19.2) | 28.0(27.5,28.5) | 9.2 |
| Tamil Nadu | Thanjavur | 74.5(74.1,74.8) | 91.3(91.0,91.5) | 16.8 | 46.8(46.4,47.2) | 72.1(71.7,72.4) | 25.3 | 14.2(13.9,14.4) | 17.1(16.8,17.4) | 3.0 |
| Tamil Nadu | Pudukkottai | 75.9(75.5,76.3) | 93.0(92.8,93.3) | 17.1 | 46.3(45.8,46.8) | 74.8(74.4,75.3) | 28.5 | 9.4(9.1,9.7) | 17.3(16.9,17.7) | 7.9 |
| Tamil Nadu | Sivaganga | 80.0(79.6,80.4) | 95.0(94.8,95.2) | 15.0 | 52.2(51.6,52.7) | 76.7(76.3,77.2) | 24.5 | 17.0(16.6,17.4) | 17.6(17.2,18.0) | 0.6 |
| Tamil Nadu | Madurai | 78.1(77.8,78.4) | 90.8(90.6,91.0) | 12.8 | 54.1(53.8,54.5) | 72.6(72.3,72.9) | 18.5 | 13.3(13.1,13.6) | 17.1(16.8,17.4) | 3.8 |
| Tamil Nadu | Theni | 76.1(75.6,76.6) | 94.9(94.7,95.2) | 18.8 | 49.6(49.0,50.2) | 76.9(76.4,77.4) | 27.3 | 19.9(19.5,20.4) | 17.3(16.9,17.7) | -2.6 |
| Tamil Nadu | Virudhunagar | 72.9(72.5,73.3) | 93.3(93.1,93.5) | 20.3 | 43.1(42.6,43.5) | 73.8(73.4,74.2) | 30.7 | 13.7(13.4,14.0) | 14.9(14.6,15.2) | 1.2 |
| Tamil Nadu | Ramanathapuram | 72.2(71.7,72.6) | 90.4(90.1,90.8) | 18.3 | 46.0(45.4,46.5) | 69.4(68.9,69.9) | 23.4 | 12.2(11.8,12.6) | 17.6(17.2,18.0) | 5.4 |
| Tamil Nadu | Thoothukkudi | 77.0(76.7,77.4) | 93.7(93.4,93.9) | 16.6 | 42.5(42.1,43.0) | 74.7(74.2,75.1) | 32.1 | 18.9(18.5,19.2) | 19.5(19.2,19.9) | 0.7 |
| Tamil Nadu | Tirunelveli | 79.9(79.6,80.2) | 92.4(92.2,92.6) | 12.5 | 44.7(44.4,45.1) | 75.3(75.0,75.6) | 30.5 | 14.3(14.0,14.5) | 14.9(14.6,15.1) | 0.6 |
| Tamil Nadu | Kanniyakumari | 80.5(80.1,80.8) | 91.4(91.2,91.7) | 11.0 | 51.0(50.6,51.5) | 76.0(75.6,76.4) | 24.9 | 17.9(17.6,18.2) | 14.1(13.8,14.5) | -3.8 |
| Tamil Nadu | Dharmapuri | 76.0(75.6,76.5) | 91.9(91.6,92.2) | 15.9 | 53.5(53.0,54.1) | 70.9(70.5,71.4) | 17.4 | 13.3(13.0,13.7) | 26.8(26.3,27.2) | 13.4 |
| Tamil Nadu | Krishnagiri | 67.8(67.3,68.2) | 92.8(92.5,93.0) | 25.0 | 43.3(42.8,43.7) | 75.2(74.8,75.6) | 32.0 | 13.5(13.1,13.8) | 23.8(23.4,24.2) | 10.4 |
| Tamil Nadu | Coimbatore | 72.6(72.3,72.9) | 92.5(92.4,92.7) | 19.9 | 56.7(56.4,57.0) | 80.0(79.7,80.3) | 23.3 | 15.2(15.0,15.4) | 15.2(14.9,15.4) | 0.0 |
| Tamil Nadu | Tiruppur | 78.0(77.7,78.4) | 91.4(91.2,91.6) | 13.4 | 47.3(46.9,47.7) | 70.4(70.1,70.8) | 23.1 | 13.8(13.5,14.1) | 17.8(17.5,18.1) | 4.0 |
| Puducherry | Yanam | 66.9(64.3,69.4) | 86.4(84.5,88.2) | 19.5 | 37.9(35.3,40.5) | 68.5(66.0,71.0) | 30.6 | 11.6(9.9,13.3) | 11.7(10.0,13.4) | 0.1 |
| Puducherry | Pondicherry | 72.5(72.0,73.1) | 90.6(90.3,91.0) | 18.1 | 54.6(54.0,55.2) | 79.0(78.5,79.5) | 24.4 | 22.9(22.4,23.5) | 14.3(13.9,14.8) | -8.6 |
| Puducherry | Mahe | 78.9(76.5,81.2) | 86.3(84.3,88.3) | 7.4 | 47.3(44.4,50.2) | 71.6(69.0,74.2) | 24.3 | 4.6(3.4,5.9) | 8.9(7.2,10.5) | 4.2 |
| Puducherry | Karaikal | 75.7(74.5,76.9) | 88.6(87.7,89.5) | 12.9 | 46.8(45.5,48.2) | 73.3(72.1,74.5) | 26.5 | 14.0(13.0,14.9) | 14.0(13.1,15.0) | 0.1 |
| Andaman & Nicobar Islands | Nicobars | 67.6(64.3,70.9) | 92.4(90.5,94.3) | 24.8 | 21.8(18.9,24.8) | 37.7(34.3,41.1) | 15.8 | 1.9(0.9,2.8) | 4.0(2.6,5.3) | 2.1 |
| Andaman & Nicobar Islands | North & Middle Andaman | 70.7(68.9,72.6) | 90.7(89.5,91.8) | 19.9 | 48.6(46.6,50.6) | 34.8(32.8,36.7) | -13.9 | 2.1(1.6,2.7) | 4.2(3.4,5.0) | 2.1 |
| Andaman & Nicobar Islands | South Andamans | 79.0(77.9,80.1) | 89.2(88.3,90.0) | 10.2 | 48.9(47.6,50.2) | 35.9(34.6,37.2) | -13.0 | 2.1(1.7,2.5) | 3.2(2.8,3.7) | 1.2 |
